# Numbering-Up Flow Electroreduction of α,β-Unsaturated Carbonyls from Gram to Pilot-Relevant Scale

**DOI:** 10.1021/acs.oprd.6c00175

**Published:** 2026-06-24

**Authors:** Tribani Boruah, Sagar Arepally, Rebecca L. Melen, Thomas Wirth

**Affiliations:** † School of Chemistry, 2112 Cardiff University, Main Building, Park Place, Cardiff CF10 3AT, Cymru/Wales, U.K.; ‡ Cardiff Catalysis Institute, School of Chemistry, Cardiff University, Translational Research Hub, Maindy Road, Cardiff CF24 4HQ, Cymru/Wales, U.K.; § School of Chemistry, Cardiff University, Main Building, Park Place, Cardiff CF10 3AT, Cymru/Wales, U.K.; ∥ Cardiff Catalysis Institute, School of Chemistry, Cardiff University, Translational Research Hub, Maindy Road, Cardiff CF24 4HQ, Cymru/Wales, U.K.

**Keywords:** flow electroreduction, dual-sided cathode, scalable numbering-up, single-pass reduction

## Abstract

A scalable flow electrochemical platform achieves up to 9.2 g/h productivity at a 100 g scale using an innovative three-electrode reactor design. Dual-sided cathodes enable 16 parallel channels across eight modules, maintaining 1 min residence time without reoptimization. The methodology processes diverse α,β-unsaturated carbonyl substrates (chalcones, heterocyclic enones, esters, ketones, carboxylic acids), explored 18 examples with broad functional group tolerance. The average productivity improvement across the substrates is 75-fold than the batch method with consistent performance from gram to pilot scale, establishing a cost-effective pathway for the electrochemical reduction reaction.

## Graphical abstract

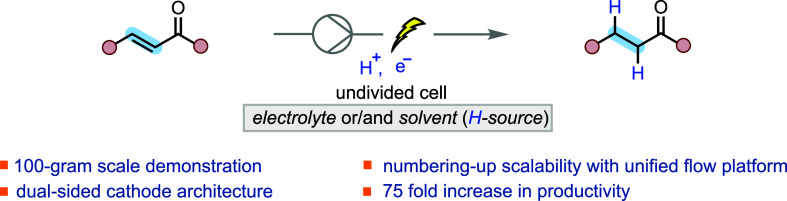

## Introduction

The transition from laboratory-scale electrochemical transformations to industrial production represents one of the most significant challenges in modern synthetic chemistry.^1,2^ Despite the renaissance in organic electrosynthesis since 2000, with numerous elegant methodologies demonstrating remarkable selectivity and functional group tolerance, the practical implementation of these reactions at meaningful scales remains limited.^3^ This gap between academic discovery and industrial application stems primarily from the inherent limitations of batch electrochemical reactors when scaling beyond gram quantities.^1^ The specific case of electrochemical hydrogenation illustrates this scale-up paradox.^4^ Compared to conventional hydrogenation, electrochemical reduction avoids external hydrogen and pressurized systems, providing safer, more controllable, and readily scalable processes. Although batch electrochemical hydrogenation has demonstrated chemical feasibility across various substrate classes,^5–12^ its process scalability remains largely unexplored. Recent surveys indicate that among approximately 60 reported large-scale (≥20 g) electrosynthesis, flow electrochemical hydrogenations account for less than 5%, with single-pass implementations essentially absent from the literature.^13^ This scarcity reflects not a lack of chemical interest but rather the absence of validated scale-up protocols and engineering solutions that address the practical challenges of implementing electrochemical hydrogenation at production scales.^4^ Traditional batch systems face fundamental challenges in scale-up, including inefficient electrode scaling, mass-transport limitations due to large interelectrode distances, safety concerns associated with hydrogen evolution in reduction reactions, excessive solvent and electrolyte consumption, and difficulties in maintaining consistent process control during scale transitions.^14^ These limitations have constrained electrochemical methods to niche applications despite their potential for sustainable synthesis. Flow electrochemical reactors directly address these limitations through reactor architectures that minimize diffusion lengths, increase surface-to-volume ratio, and enable precise control of residence time, mass transport, and current density.^15–18^ The reduced interelectrode distance minimizes *iR* (ohmic), which is critical for maintaining consistent performance during scale-up.^19,20^ At the same time, continuous operation and numbering-up strategies (parallelization of identical modules) offer a predictable path from gram scale demonstrations to kilogram/pilot throughput without the unpredictable engineering leaps required for single large batch cells.^21^ These attributes also improve safety (continuous removal/management of gaseous byproducts),^22^ reproducibility, and process intensification which are the key considerations for industry adoption.^23,24^ Industrial precedents demonstrate the viability of flow electrosynthesis at production scales.^24^ Thoughtful electrochemical reactor design can convert fundamental reductions into scalable and process-relevant operations.^13^ Sawant and co-workers reported an elegant multigram benchmark through the electrochemical reduction of maleic acid in a parallel-plate flow reactor, achieving high efficiency while enabling a recycling of the sulfuric acid electrolyte.^17^ In a complementary advance, Baran and co-workers established the feasibility of large-scale flow electroreduction by performing a 100 g electrochemical Birch reduction.^18^ These successes highlight the economic and technical feasibility of electrochemical flow processing, yet significant practical challenges persist. Electrode and cell designs that translate laboratory performance into pilot throughput while preserving mass-transfer limited reaction kinetics, energy efficiency, and product quality are still underdeveloped. Process-development practices for electrochemical hydrogenation, systematic mapping of critical process parameters (CPPs),^25^ scalable current and flow control, modular hardware for simple numbering-up, and robust validation protocols are not yet standardized across the field. Despite the limited availability of fully GMP (Good Manufacturing Practice) compliant electrochemical systems, ongoing developments in modular flow technologies and industrial collaborations indicate strong progress toward their implementation.^26^ The numbering-up strategy presented here aligns well with these trends, offering a scalable and adaptable approach with clear potential for future integration into GMP manufacturing. Continued advancements in reactor design, automation, and process control are expected to further accelerate the development of robust GMP ready electrochemical platforms.

In this work, we present a process development framework and modular flow reactor platform tailored for scalable electrochemical hydrogenation (Scheme 1). We address the engineering challenges necessary to translate well-established reductions from the laboratory to pilot-relevant throughput. Our approach combines (i) a modular cost-effective dual cathode flow cell architecture designed for straightforward numbering-up and efficient electrode utilization to enhance the productivity; (ii) systematic control and scaling rules for CPPs (current density, residence time, mixing/mass transfer metrics, electrolyte concentration, and temperature) that preserve faradaic efficiency during scale escalation; and (iii) a validation protocol including NMR monitoring and stepwise scale demonstration from gram to pilot scale. The results bridge a critical gap between academic electrochemical hydrogenation reports and industrial process needs, enabling wider, safer, and more energy-efficient adoption of flow electrochemical reductions in process chemistry and manufacturing. This study connects academic research with real-world industrial use by demonstrating how flow electrochemical reductions can be made to be safe, efficient, and scalable. The reactor design, scale-up strategy, and pilot-scale results provide a clear guide for applying these reactions in practice.

**Scheme 1:**
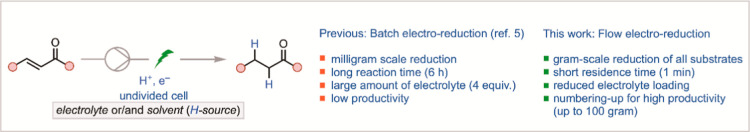
Scalable Electro-Reduction from Gram to Pilot Scale

The following sections detail our modular reactor design, scale-up methodology, validation procedures, and pilot-relevant scale demonstration, providing a comprehensive roadmap for implementing scalable flow electrochemical reduction processes in both academic and industrial settings.

## Results and Discussion

The development of a scalable continuous-flow electrochemical hydrogenation methodology commenced with optimization of chalcone (**1a**) as a model substrate. Initial conditions were adapted from the reported batch electrochemical method,^5^ employing ammonium acetate as both a supporting electrolyte and potential hydrogen source in a DMSO/EtOH (4:1) solvent system. The initial investigation centered on physical reactor parameters, where interelectrode distance emerged as a critical factor governing residence time and mass transfer efficiency. Evaluation of gasket thickness revealed that a 0.25 mm electrode distance provided optimal performance, achieving complete reduction in a short residence time (Table 1A, entry 2). In contrast, a 0.10 mm gasket resulted in an insufficient residence time and incomplete conversion (Table 1A, entry 2). Throughput optimization was achieved through systematic variation of flow rate with proportional current adjustment to maintain a constant charge-to-substrate ratio (Table 1C, see the Supporting Information for full details). The inverse relationship between the flow rate and residence time, coupled with proportional current adjustment, provides a powerful scaling strategy (Table 1C). By increasing the flow rate from 0.05 to 0.3 mL/min, the residence time decreased 6-fold while maintaining yields above 75% under optimal conditions (Table 1C). This demonstrates that productivity can be enhanced without losing conversion efficiency, which is a critical consideration for industrial implementation where throughput must be maximized while maintaining product quality.

**Table 1:**
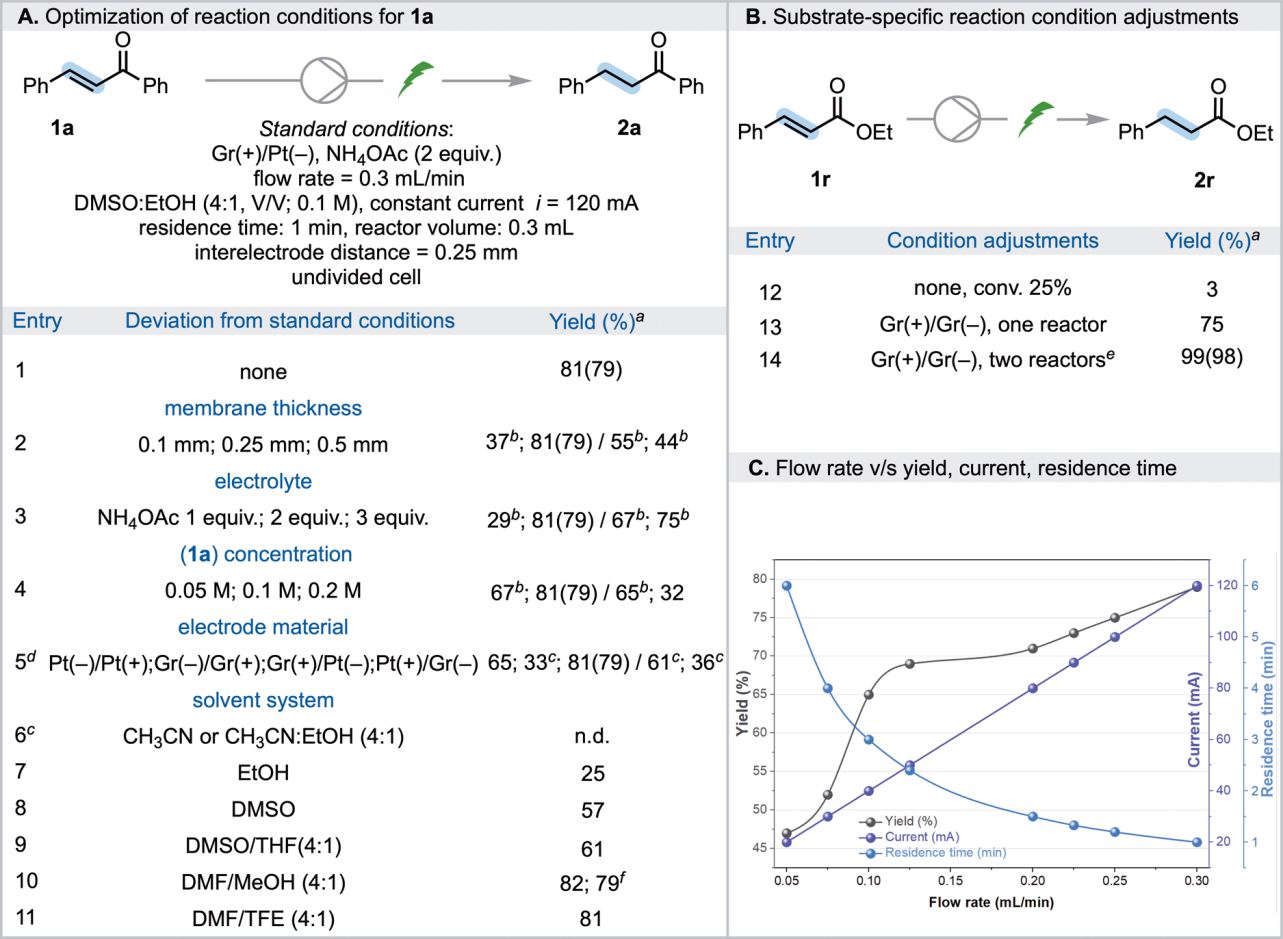
Optimization Studies^a^

a Isolated yields are given in parentheses, conversions and yields were measured by crude ^1^H NMR spectroscopy using dibromomethane as an internal standard.

b Pt­(+)/Pt­(−).

c 100 mA constant current.

d An insoluble reaction mixture was obtained.

e Electrolysis was carried out using two flow reactors in series, and a constant current was applied at each reactor.

f Back-pressure regulator (1 bar). n.d. = not detected.

This approach resulted in a flow rate of 0.30 mL/min with 120 mA applied current, delivering 75% yield with a residence time of 1.0 min. Electrolyte optimization established that 2.0 equiv of ammonium acetate provided the optimal balance between ionic conductivity and hydrogen donor availability, yielding 67% conversion (Table 1A, entry 3). Lower loading (1.0 equiv) resulted in diminished performance due to insufficient conductivity, while higher loading (3.0 equiv) offered no significant improvement (Table 1A, entry 3). The substrate concentration was constrained by electrolyte solubility in the DMSO/EtOH system, with 0.10 M identified as the maximum reliable concentration for consistent performance (Table 1A, entry 4). Screening of electrode combinations (Table 1A, entry 5) identified Pt(−)/Gr­(+) as the most effective. Solvent system evaluation revealed that DMSO/EtOH (4:1) delivered optimal yields (78%) but presented practical limitations due to solidification at ambient temperatures. Alternative systems were investigated (Table 1A, entries 6–11), with DMF/MeOH (4:1) providing comparable performance (82% yield, Table 1A, entry 10) without temperature-dependent handling issues. Under a back pressure of 1 bar (entry 10), no significant change in the yield was observed; however, segmented flow at the reactor outlet indicated formation of hydrogen. However, the amount of gas generated was minimal and did not impact the reaction performance or necessitate the use of a back pressure regulator under the applied conditions.

The electrode materials proved to be a decisive factor that varied with substrate class (Table 1B, entry 12–14). For chalcone and its derivatives, a graphite anode paired with a platinum cathode afforded the highest efficiency (Table 1A, entry 1 and entry 5), whereas ethyl cinnamate, a structurally similar yet electrochemically distinct substrate, required switching the cathode to graphite, improving the yield from ∼3% to 75% (Table 1B, entry 12–14) with incomplete conversion, while connecting two flow reactors in a series mode enabled >99% conversion and 98% yield (see the Supporting Information for full details).

Having successfully optimized the electrochemical hydrogenation of α,β-unsaturated ketones (Table 1A), the generality of the protocol across diverse α,β-unsaturated substrates was evaluated. All reactions were conducted at 2 g scale using 4-parallel reactors, enabling simultaneous processing of multiple substrates while maintaining identical reaction conditions. This parallel approach not only increased throughput but also demonstrated the methodology’s suitability for larger-scale operations. Chalcones bearing electron-donating groups such as methyl, benzyloxy, and phenyl (**2a**–**2d**) were efficiently hydrogenated under mild conditions, giving yields around 80% with excellent productivity. Substrates containing dialkoxy groups or other reduction-sensitive functionalities (**2e**–**2g**) also reacted smoothly, delivering moderate yields while retaining these sensitive groups indicating an important advantage for complex molecule synthesis. The scope extends beyond chalcones, α,β-unsaturated esters (**2r**, **2s**), ketone (**2t**), and carboxylic acid (**2u**) were successfully reduced by slightly increasing the applied current, affording moderate to good yields without compromising selectivity. However, **1p** and **1q** with nitro and aldehyde moieties did not afford the desired products, and complete degradation of the starting material was observed in both cases, likely due to their reduction and competing side reactions. Heterocycle-containing enones (**2i**–**2n**) performed particularly well, achieving good to excellent yields, which highlights the method’s compatibility with medicinally relevant scaffolds. Chemoselectivity was also evident for a substrate containing an alkyne moiety **1h**, the C=C bond was reduced preferentially, while the alkyne remained largely intact, albeit with slightly lower yields. To clearly demonstrate the advantages of the flow system, its performance against the original batch electrochemical reduction was evaluated.^5^ The batch method required 4 equiv of electrolyte and took 6 h on a 1 g scale of **1a**, whereas in the 4 parallel reactor flow setup, only 2 equiv. electrolyte and half the amount of solvent are necessary and the reaction time is reduced to only 1 min (see the Supporting Information for full details). At the 10 g scale, the entire process finished in 400 min, delivering **2a** in 79% yield (7.9 g) with a productivity of 1.2 g/h. During the 100 g scale reaction, a gradual increase in cell voltage (from 2 to 3 V) was observed over the 8 h continuous operation. However, this did not appear to significantly affect the reaction performance.

The bar graph comparison (Table 2) illustrates productivity enhancement of 60-fold for **2a**, 126-fold (**2b**), 18-fold (**2j**), and 93-fold (**2q**) for the flow protocol versus batch report. These data underscore the flow system’s capacity for substantially increased throughput. A key engineering innovation enabling large-scale implementation is the development of a three-electrode reactor design. In this configuration, each reactor module contains a central electrode functioning as an active cathode on both sides, creating two electrochemical chambers within a single unit which can be used in series and parallel configuration (Figure 1a). This dual-sided cathode design doubles productive electrode area and enables parallel electro-reductions within minimal footprint (Figure 1b). The electrochemical reduction was demonstrated at 100 g scale using 8 parallel reactor modules with 16 cells, each act as one single reactor (Scheme 2e). The single-pass reaction conditions optimized at laboratory scale were directly applied to 0.48 mol (100 g) of substrate **1a** without further reoptimization, delivering 73 g of product **2a** (73% yield) with 83% conversion (Scheme 2c). A productivity of 9.2 g/h was achieved while maintaining the ∼1 min residence time, with continuous operation for about 8 h demonstrating operational reliability. This approach provides a cost-effective method through parallel processing while enhancing productivity. Productivity analysis reveals the flow system achieves 1.2–1.4 g/h for most substrates at laboratory scale, representing an average of 75-fold improvement over batch methods. At industrial scale, the 9.2 g/h productivity represents a significant advancement for electrochemical reduction reactions. The methodology demonstrates consistent performance from 2 to 100 g scale without efficiency losses, with reduced electrolyte requirements (2 vs 4 equiv) and solvent consumption compared to batch methods. The developed platform addresses key scalability challenges through engineering innovation (three-electrode design), parallel processing capability (8 modules, 16 cell configuration), and linear scalability without performance degradation. These advancements establish a robust foundation for industrial implementation of electrochemical reduction reactions, overcoming limitations that have historically restricted adoption of electrochemical methods in production environments.

**Table 2:**
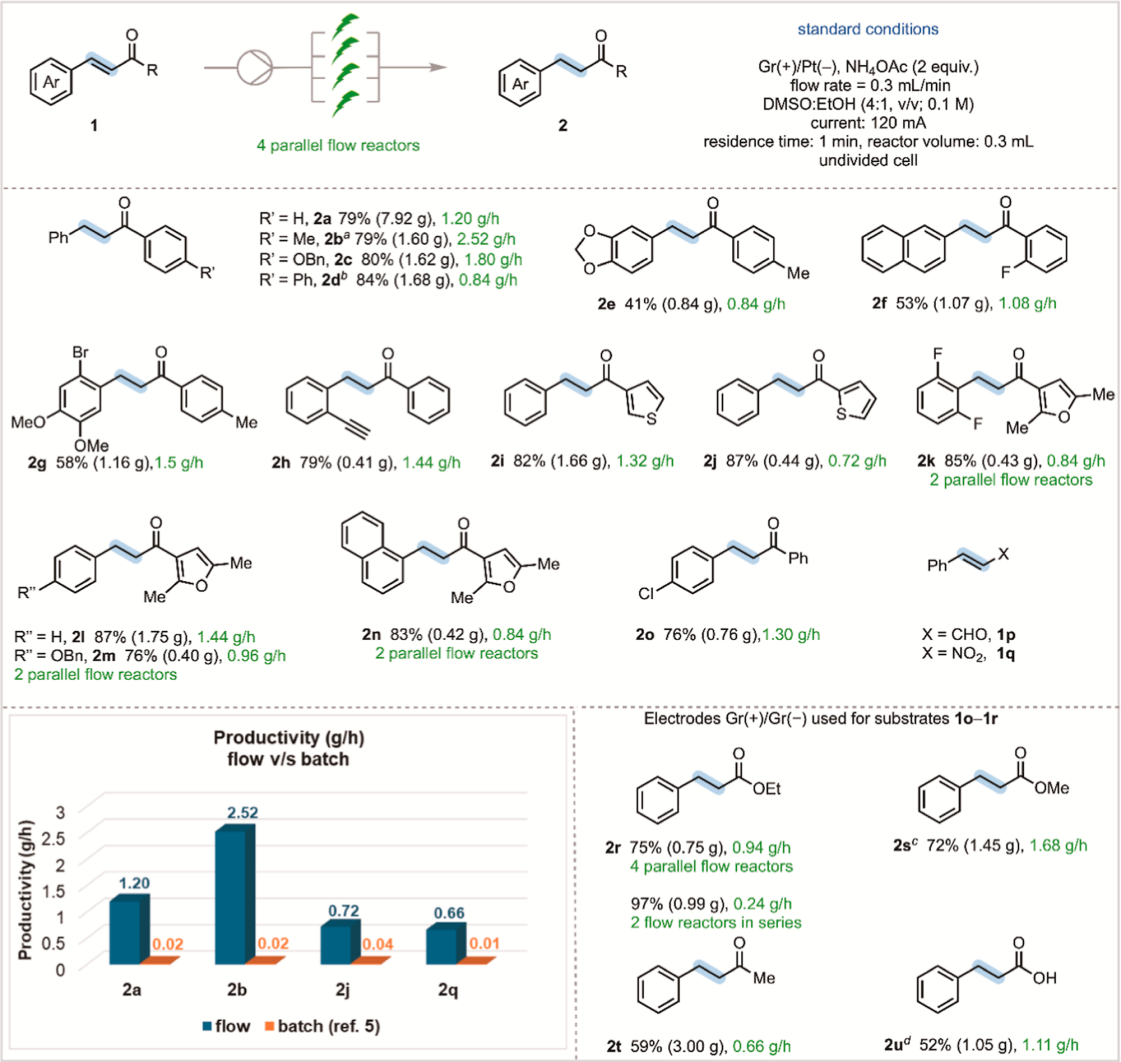
Substrate Scope and Comparison of Productivity with Batch Report^a^

a **1b** (0.2 M) and 160 mA were used.

b **1d** (0.05 M) and 80 mA were used.

c **1s** (0.2 M) and 260 mA were used.

d **1u** (0.2 M) and 300 mA were used.

**Figure 1:**
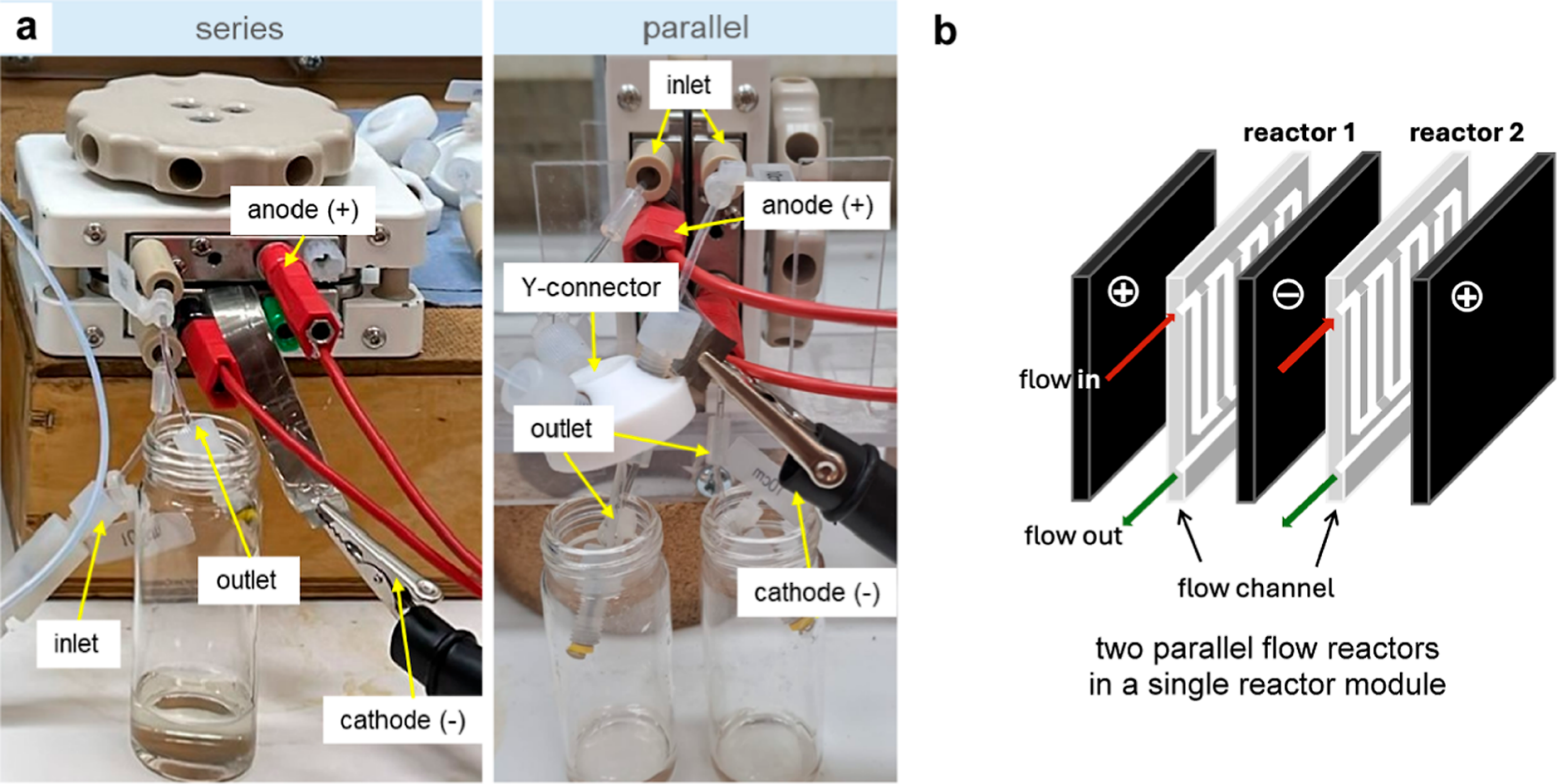
Development of a cost-effective reactor modulation with 3-electrode setup in parallel and series: (a) series and parallel setup, (b) schematic representation of two parallel flow reactors in a single reactor module.

**Scheme 2:**
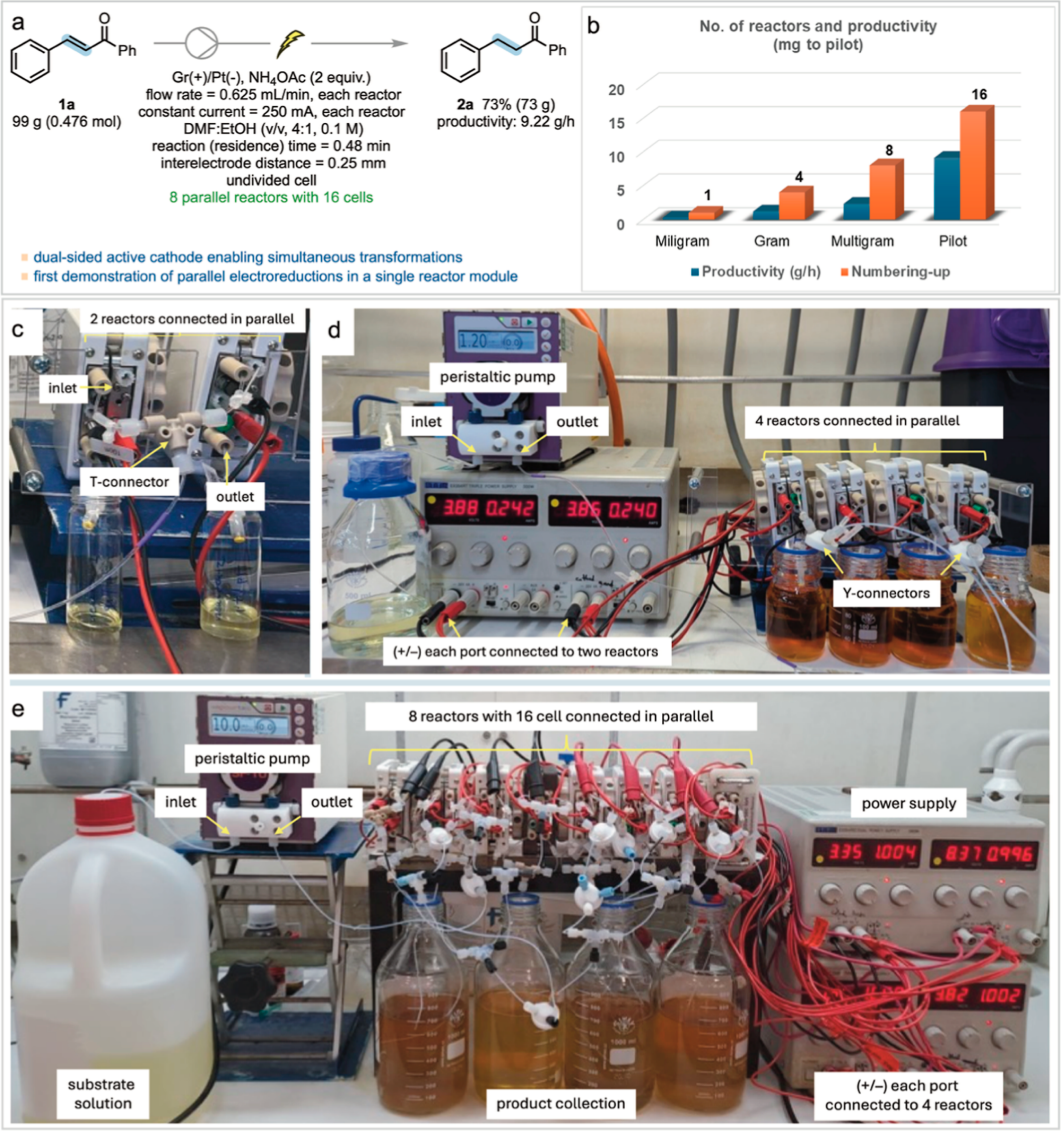
(a) 100 Gram Scale Demonstration, (b) Comparison between Productivity and Numbering-up from Milligram to Pilot Scale, (c) 2 Reactors (1 gram Scale), (d) 4 Reactors (10 gram Scale), (e) 8 Reactors with 16 Cells (100 Gram Scale)

## Conclusion

The flow electrochemical platform demonstrates industrial viability for reduction reactions through a three-electrode reactor design that enables parallel processing in 16 channels across eight modules. This configuration achieves 9.2 g/h productivity at 100 g scale with 73% yield while maintaining consistent performance from gram to hundred-gram scale without reoptimization. Compared to batch methods, the platform offers 75-fold higher productivity, reduced electrolyte consumption (2 vs 4 equiv), continuous operation capability (>5 h), and broad substrate scope across α,β-unsaturated carbonyl compounds (55–85% yield). This work establishes a practical pathway for implementing electrochemical reductions at the manufacturing scale, directly addressing the scalability limitations that have historically restricted electrochemical methods to laboratory applications.

## Experimental Section

### Flow-Electrolysis Optimization Details

#### General Procedure for Flow-Electrolysis Optimization Studies (GP2)

A solution of chalcone (0.1 M, 1 equiv) and the supporting electrolyte (NH_4_OAc) was prepared in the designated solvent system. This solution was pumped into a Vaportec Ion electrochemical flow reactor (reactor volume: 0.3 mL; interelectrode distance: 0.25 mm) using a syringe pump or peristaltic at the required flow rate. Electrolysis was performed under constant current conditions (charge in F/mol) using electrodes with an effective surface area of 12 cm^2^. After reaching steady-state conditions (equivalent to two reactor volumes), the initial outflow was discarded, and the product stream was collected in a glass vial. An aliquot corresponding to 0.3 mmol was worked up with diethyl ether: water (1:5), and the organic lager was concentrated under reduced pressure. The residue was diluted with 300 μL of a 1 M solution of CH_2_Br_2_ in CDCl_3_. Conversion and yield were determined by integrating the methylene (CH_2_) triplet signals in the ^1^H NMR spectrum.

### General Flow-Electrolysis Procedure

#### General Flow-Electrolysis Protocol for the Reduction of α,β-Unsaturated Aryl/Heteroaryl Ketones (GP3)

##### Parallel Configuration

A solution of α,β-unsaturated aryl ketone **1a**, **S1**–**S13** (0.1 M, 1 equiv) in dimethyl sulfoxide and ethanol (DMSO/EtOH; 1:1; v/v; 0.1 M) containing NH_4_OAc (0.1 M, 2 equiv), was introduced into a Vaportec ion electrochemical flow reactor (each reactor volume: 0.3 mL; interelectrode distance: 0.25 mm) using a peristaltic pump and with a flow rate of 0.3 mL/min. Electrolysis was carried out under a constant current employing graphite as the anode and platinum as the cathode (for α,β-unsaturated aryl and hetero aryl ketone) and graphite as anode and cathode (for esters, ketones, and acids), with both electrode having an effective surface area of 12 cm^2^. After reaching steady-state conditions (two reactor volumes discarded), the product stream was collected in a glass vial. After electrolysis, both electrodes were thoroughly rinsed with methanol (5 mL per reactor) without dismantling the reactor, and the reaction mixture was diluted with water (5 × the reaction volume), the aqueous layer was extracted three times with diethyl ether, dried over MgSO_4_, and filtered. The crude product was purified by flash column chromatography using petroleum ether (PE)/ethyl acetate (EA) as the eluent, affording the desired class of derivatives.

## General Flow-Electrolysis Protocol for Scale-Up (GP4)

10 g scale using 4 reactor setup in parallel configuration for reduction of chalcone:

Reaction performed at 10 g scale in parallel configuration, chalcone (**1a**) as the model substrate (0.1 M, 1 equiv, 48 mmol, 10 g), solvent: DMSO/EtOH, NH_4_OAc (0.1 M, 2 equiv, 96 mmol, 7.50 g), retention time 1 min, overall reaction time 6 h 40 min, conv.: 92%, NMR yield: 81%, isolated yield: 7.92 g, 38 mmol, 79%, productivity: 1.20 g/h (Scheme 2d).

A 100 g scale reaction was conducted using a modified single-unit dual-reactor of a 16-reactor setup in a parallel configuration:

Reaction performed at 100 g scale in parallel configuration, chalcone as the model substrate (0.1 M, 1 equiv, 476 mmol, 99 g), solvent: DMF/EtOH, NH_4_OAc (0.1 M, 2 equiv, 952 mmol, 74.26 g), retention time 1 min, overall reaction time 7 h 55 min, conv.: >83%, isolated yield: 73 g, 348 mmol, 73%, productivity: 9.22 g/h, total current used: 4 A (250 mA per cell), total flow rate: 10 mL/min, total reactor volume: 4.8 mL (Scheme 2e).

## Supplementary Material



## Data Availability

Information on the data underpinning this publication, including access details, can be found in the Cardiff University Research Data Repository at https://doi.org/10.17035/cardiff.32704476.

## Notes

^1^H NMR and ^13^C NMR spectroscopic data and mass spectrometric data of starting materials (**1a** to **1u**) and products (**2a** to **2u**); optimization details; and deuterium experiments of **2a** (PDF)

## ⊥

Department of Chemistry and Biotechnology, Tallinn University of Technology (TalTech), Akadeemia tee 15, SCI-414, Tallinn 12618, Estonia
