# Supplementary material for: Numbering-Up Flow Electroreduction of α,β-Unsaturated Carbonyls from Gram to Pilot-Relevant Scale
Source: Org Process Res Dev. 2026 Jun 24;30(7):2067–74. doi: 10.1021/acs.oprd.6c00175 (PMC13386526; doi:10.1021/acs.oprd.6c00175)

Supporting information

Numbering-Up Flow Electroreduction of *α,β*-Unsaturated Carbonyls from Gram to Pilot-Relevant Scale

Tribani Boruah,[a,b] Sagar Arepally,*[a] Rebecca L. Melen,*[b] Thomas Wirth*[a]

[a] Ms Tribani Boruah, Dr. Sagar Arepally, Prof. Dr. Thomas Wirth, School of Chemistry, Cardiff University, Main Building, Park Place, Cardiff CF10 3AT, Cymru/Wales, UK. Emails: ArepallyS@cardiff.ac.uk, wirth@cf.ac.uk.

[b] Ms Tribani Boruah , Prof. Dr. Rebecca L. Melen, Cardiff Catalysis Institute, School of Chemistry, Cardiff University, Translational Research Hub, Maindy Road, Cathays, Cardiff CF24 4HQ, Cymru/Wales, UK. Email: MelenR@cardiff.ac.uk.

Contents

1. General information S4

1.1. Reagents, solvents and experimental conditions S4

1.2. Analytical techniques S4

1.3. Compound purification S4

2. Experimental section. S5

2.1. Electrochemical setup. S5

2.1.1 Batch electrochemical set-up S5

2.1.2 Batch electrochemical reaction using IKA Electrasyn S5

2.1.3 Flow electrochemical set-up S5

2.2. Details for the synthesis of all starting materials. S6

General procedure (GP1) for the synthesis of α,β-unsaturated aryl ketones. S6

3. Flow-electrolysis optimization details S7

3.1.General procedure for flow-electrolysis optimization studies (GP2) S7

Table S1: Optimization of current and flow-rate S8

Table S2: Optimization of solvent S9

4. General Flow-electrolysis Procedure S9

4.1. General flow-electrolysis protocol for the reduction of *α,β*- unsaturated aryl/hetero-aryl ketones (GP3) S9

4.1.1. Method A S9

4.2. General flow-electrolysis protocol for the reduction of *α,β*- unsaturated aryl/hetero aryl ketones (GP4) S15

4.2.1. Method B: S15

5. General Flow-electrolysis Protocol for Scale-Up S15

6. Cost Efficient Electrochemical Reactor Design: A Modified Single-Unit Approach for Dual-Reactor Operation S17

7. Deuterium experiments S18

Table S3: Faradaic efficiency and F/mol of products S21

8. Details of reactor and electrodes cleaning S21

9. NMR spectral data S22

9.1. Characterization data for starting materials S22

9.2. Characterization data for α,β-unsaturated ketone derivatives S27

10. NMR spectra S40

10.1. NMR spectra for α,β-unsaturated ketone derivatives.................................. S40

1. General information

1.1. Reagents, solvents and experimental conditions

All reagents were purchased from Alfa Aesar, Sigma-Aldrich, Fluorochem, Acros Organics, Fisher Scientific and used without further purification, except otherwise stated. Dry solvents such as THF, toluene and acetonitrile were obtained after passing these previously degassed solvents through activated alumina columns (MBraun, SPS-800). DMF and DMSO solvents used without further purification.

1.2. Analytical techniques

NMR-spectra: NMR spectra's were recorded on Bruker DPX 300, 400 or 500 spectrometers. All spectral data was acquired at 295 K. Deuterated solvents for NMR analysis were purchased from Sigma Aldrich. ^{1}H and ^{13}C chemicals shifts (δ) are quoted in parts per million (ppm) against tetramethylsilane (TMS, δ = 0.00 ppm) and were internally referenced to residual $CHCl_3$ (7.26 ppm for ^{1}H, 77.16 ppm for ^{13}C).^{19}F chemicals shifts (δ) are quoted in parts per million (ppm) and were calibrated using absolute referencing to the ^{1}H NMR spectrum. Coupling constants (*J*) are reported in Hertz (Hz) to the nearest 0.1 Hz. The following abbreviations (or combinations thereof) were used to explain multiplicities: s = singlet, d = doublet, t = triplet, q = quartet, quintet, p = pentet, m = multiplet.

High-resolution mass spectra (HRMS): These were obtained by the MS service of Cardiff University on a Water LCR Premier XE-TOF. Ions were generated by the Atmospheric Pressure Ionization Techniques (APCI), Electrospray (ESI) and Electron Ionization (EI).

IR spectrum: The infrared spectra were obtained by using ATR-FTIR IRAffinity, Shimadzu instrument.

Thin layer chromatography (TLC): Carried out on Merck silica gel 60 F_{254} (0.20 mm) pre-coated aluminum sheets and were visualized using UV light (254 nm) and stained with iodine in silica.

1.3. Compound purification

Flash chromatography (Combi Flash NEXTGEN 300+ TELEDYNE ISCO) was carried out using silica gel (Redi Sep Rf Gold, 24 Gram Flash Column), eluting with the specified solvent system as mentioned. Solvents for chromatographic purification (*n*-hexane/cyclohexane/pet-ether and EtOAc) were purchased from commercial sources and used directly.

2. Experimental section.

2.1. Electrochemical setup.

2.1.1 Batch electrochemical set-up

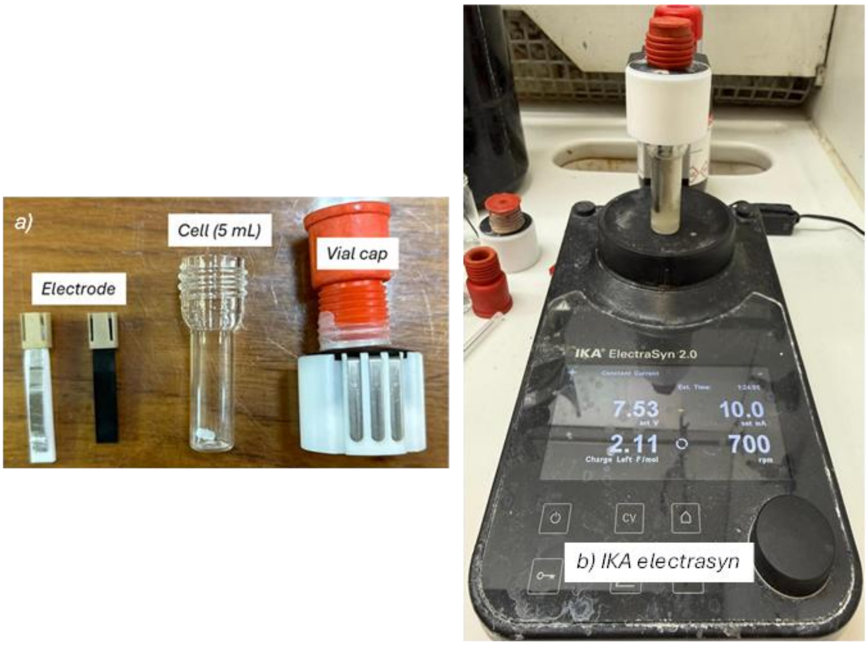

Figure S1: Batch electrochemical reaction set-up: (a) left: electrodes (cathode (Pt)), anode (Gr)) with electrode holder, right: electrochemical cell cap, middle: electrochemical cell (5 mL), b) *IKA* electra-syn set-up.

2.1.2 Batch electrochemical reaction using IKA Electrasyn

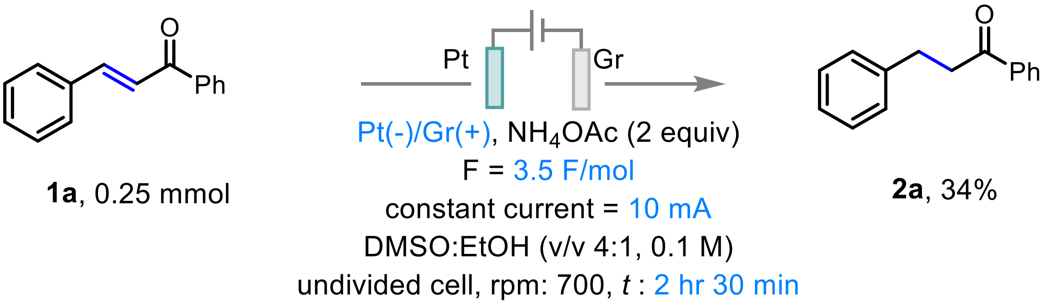

1a, 0.25 mmol

2a, 34%

Scheme S1: Batch electrochemical reaction.

2.1.3 Flow electrochemical set-up

Flow electrochemical experiments were carried out with an Ion electrochemical flow reactor from Vapourtec Ltd. (Figure S2). In this setup, working and counter electrodes (5 cm × 5 cm) were separated by a 0.25 mm thick FEP spacer resulting in a reactor volume of 300 µL and exposed electrode surface area of 12 cm^2. Rigid graphite (99.95% purity), Platinum foil (99.95% purity), were procured from

Goodfellow. KR Analytical Ltd Fusion 100 Touch syringe pumps were used to pump the reagent solution through the assembled undivided flow electrochemical reactor and was collected in suitable volumetric flask or vial. Aim-TTi Digital Bench Power Supply (280 W, 2 Output, 0 → 35 V, 0 → 4 A) was used for electrolysis under constant current conditions.

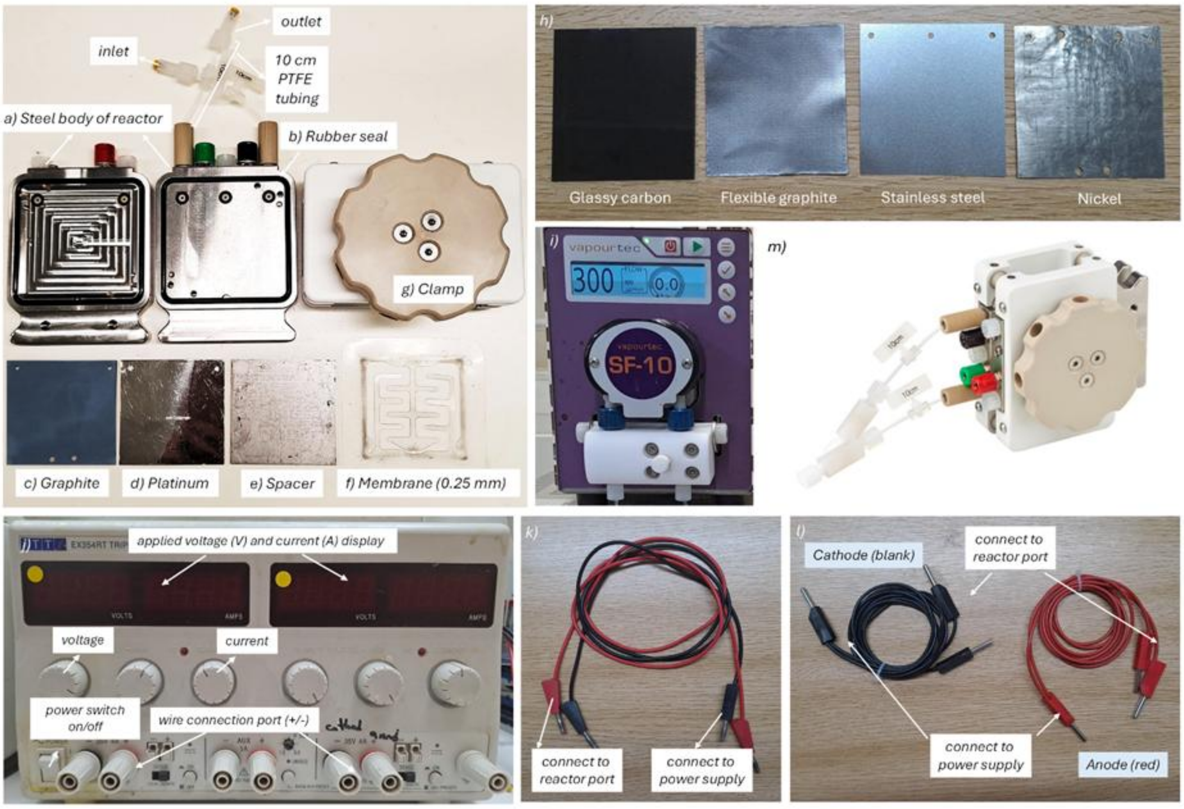

Figure S2: a) steel body of the reactor (containing inlet and outlet tubing on one side), b) rubber seal (to prevent leakage), materials used in this reaction: c) graphite d) platinum e) spacer: 1 mm f) membrane (or flow path): 0.25 mm; g) clamp (to hold the reactor), h) other electrode materials, i) peristatic pump, j) power supply; electrical wires: k) for 1 single reactor, l) for one pair of reactors, m) overall ion flow electrochemical reactor.

2.2. Details for the synthesis of all starting materials.

The starting materials, α,β-unsaturated ketones were prepared using procedures that are analogous to the respective literature procedure.[1]

General procedure (GP1) for the synthesis of α,β-unsaturated aryl ketones.

To a solution of ketone (20 mmol, 1.0 equiv) in 16 mL ethanol was added a solution of NaOH (1 g, 26 mmol, 2.6 equiv) in water (20 mL), then aldehyde (20 mmol, 1.0 equiv) was added gradually at 0 °C. The mixture allowed to warm to room temperature (25 °C) and stirred overnight. The solid product was collected by suction filtration on a Buchner funnel and washed repeatedly with cold ethanol and dried to obtained the pure solid followed by NMR analysis compared with literature.[1]

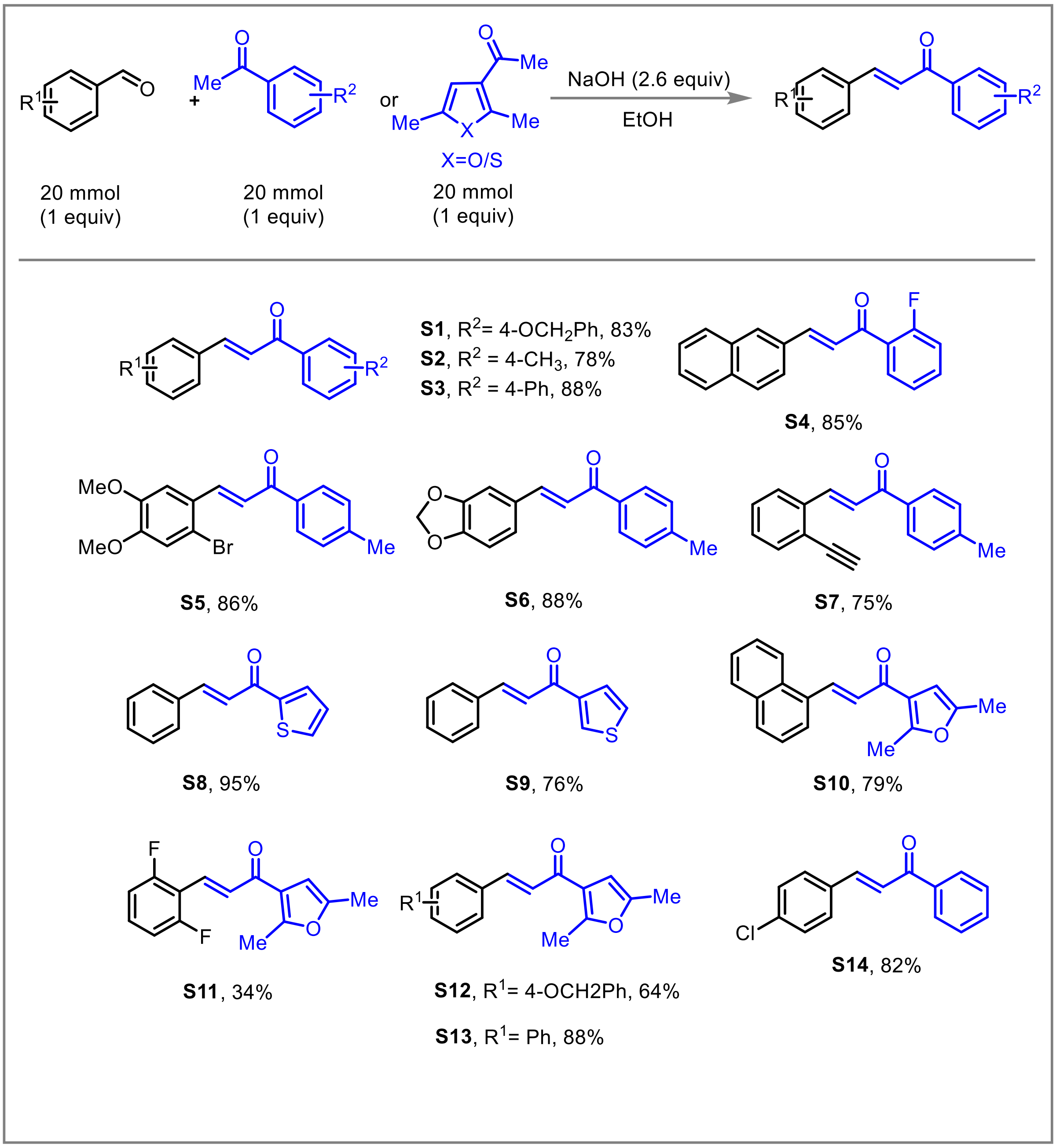

Scheme S2: Starting materials for *α,β*-unsaturated aryl and hetero aryl ketones.

Note: some of the SM spectra contains inseparable or trace amount of impurities, the purity of those determined by ^{1}H NMR using dibromomethane as internal standard prior to the flow electrochemical reaction.

3. Flow-electrolysis optimization details

3.1.General procedure for flow-electrolysis optimization studies (GP2)

A solution of chalcone (0.1 M, 1 equiv), and the supporting electrolyte (NH_4OAc or Et_4NBF_4) was prepared in the designated solvent system. This solution was pumped into a Vapourtec Ion electrochemical flow reactor (reactor volume: 0.3 mL; interelectrode distance: 0.25 mm) using a syringe

pump or peristaltic at the required flow rate. Electrolysis was performed under constant current conditions (charge in F/mol) using electrodes with an effective surface area of 12 cm^2. After reaching steady state conditions (equivalent to two reactor volumes), the initial outflow was discarded, and the product stream was collected in a glass vial. An aliquot corresponding to 0.3 mmol was work-up with diethyl ether: water (1:5) and the organic lager was concentrated under reduced pressure. The residue was diluted with 300 µL of a 1 M solution of CH_2Br_2 in $CDCl_3$, along with additional $CDCl_3$. Conversion and yield were determined by integrating the methylene (CH_2) triplet signals in the ^1H NMR spectrum.

Table S1: Optimization of current and flow-rate

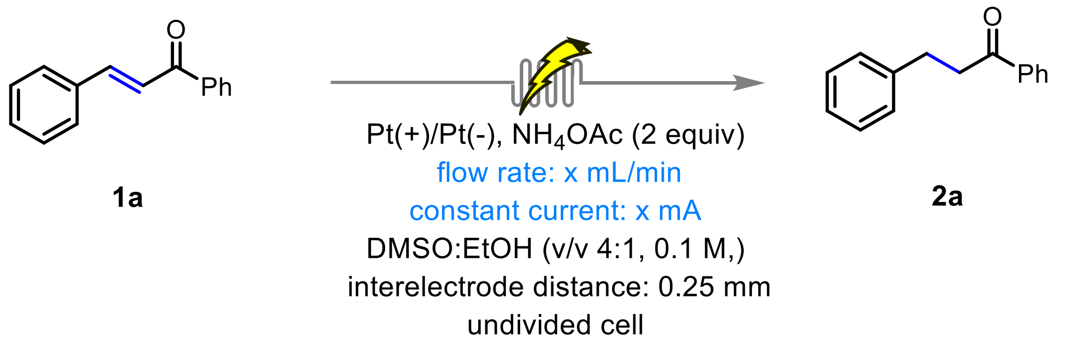

flow rate (mL/min)	current (i) (mA)	reaction time (min)	voltage (V)	yield **2a** (%)[a]	**1a** (%)[a]
0.05	20	6	2.93	47	trace
0.075	30	4	3.05	52	trace
0.1	40	3	3.1	65	trace
0.125	50	2.4	3.18	69	trace
0.2	80	1.5	3.46	71	trace
0.225	90	1.33	3.48	73	trace
0.25	100	1.2	3.6	75	trace
0.3	120	1	3.8	79	trace

[a] In all cases exact conversion was not resolved and yields determined by crude ^1H NMR using CH_2Br_2 as internal standard.
Note: above optimizations performed using single reactor without washing, no reproducibility issuse has been observed.

Table S2: Optimization of solvent

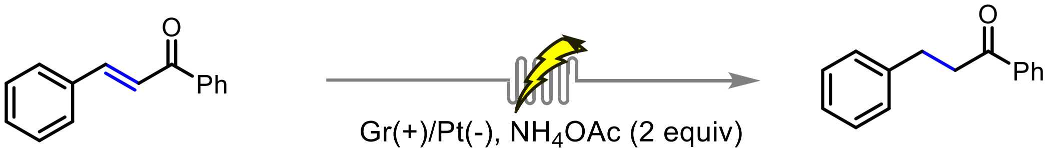

NH_4OAc(equiv)	Solvent system	Yield **2a** (%)[a]	Conv. **1a** (%)[a]
2	DMSO:THF (4:1)	61	>99
2	DMSO:THF : EtOH (3:1:1)	62	>99
2	DMSO	57	>99
2	DMF:MeOH (4:1)	82	>99
2	DMF:TFE (4:1)	81	>99
2	DMF:HFIP(4:1)	65	>90
Et_4NBr (1 equiv)	ACN:Toluene	n.d	complex mixture

[a] In all cases exact conversion was not resolved and yields determined by crude ^{1}H NMR using CH_2Br_2 as internal standard.

Note: above optimizations performed using single reactor without washing, no reproducibility issuse has been observed.

4. General Flow-electrolysis Procedure

4.1. General flow-electrolysis protocol for the reduction of *α,β*- unsaturated aryl/hetero-aryl ketones (GP3)

Note: All substrates were explored on a 2-gram scale otherwise stated, and all the solutions are prepared in 5 mL excess for the initial equilibrium of the reactors.

4.1.1. Method A

4.1.1.1. parallel configuration

Reaction protocol: A solution of *α,β*- unsaturated aryl ketone **1a**, **S1-S14** (0.1 M, 1 equiv) in solvent dimethyl sulphoxide and ethanol (DMSO:EtOH; 1:1; v/v; 0.1 M) containing NH_4OAc (0.1 M, 2 equiv), was introduced into a Vapourtec Ion Electrochemical Flow Reactor (each reactor volume: 0.3 mL;

interelectrode distance: 0.25 mm) using a peristaltic pump and a reaction flow rate of 0.3 mL/min. Electrolysis was carried out under a constant current employing a graphite as anode and platinum as cathode (for *α,β*- unsaturated aryl and hetero aryl ketone) and graphite as anode as well as cathode (for ester, ketone and acid derivatives), with effective electrode surface area: 12 cm^2. After reaching steady-state conditions (two reactor volumes discarded), the product stream was collected in a glass vial. After electrolysis, both electrodes were thoroughly rinsed with methanol (approx. 5 mL per reactor) without dismantling the reactor, and reaction mixture were diluted with 5x water with respect to reaction mixture, the aqueous layer was extracted three times with diethyl ether, dried over $MgSO_4$ and filtered. The crude product was purified by flash column chromatography using petroleum ether (PE)/ethyl acetate (EA) as the eluent, affording the desired class of derivatives.

Connectivity of reactors in parallel configuration:

2-reactor setup: A dual-reactor configuration was employed to enable parallel processing of the reaction mixture under identical flow conditions. The reaction solution was pumped through a peristaltic pump to a Y- or T-shaped fluid connector, which diverted the flow stream into two independent inlet tubing, each feeding one of the two reactors. This configuration allowed identical reaction conditions to be maintained across both reactors simultaneously, and the effluent from each reactor was collected separately in distinct vials at the outlet, allowing for direct comparison of reaction outcomes, and product yields under identical experimental conditions as shown in Figure S3. The system's modular design facilitated reproducibility, scalability, and efficient screening of reaction parameters across multiple setups.

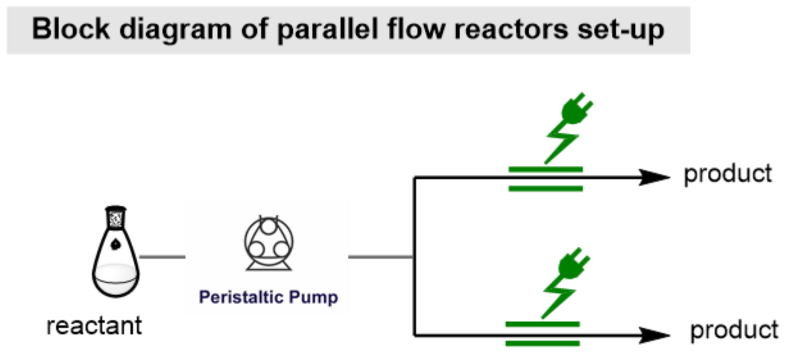

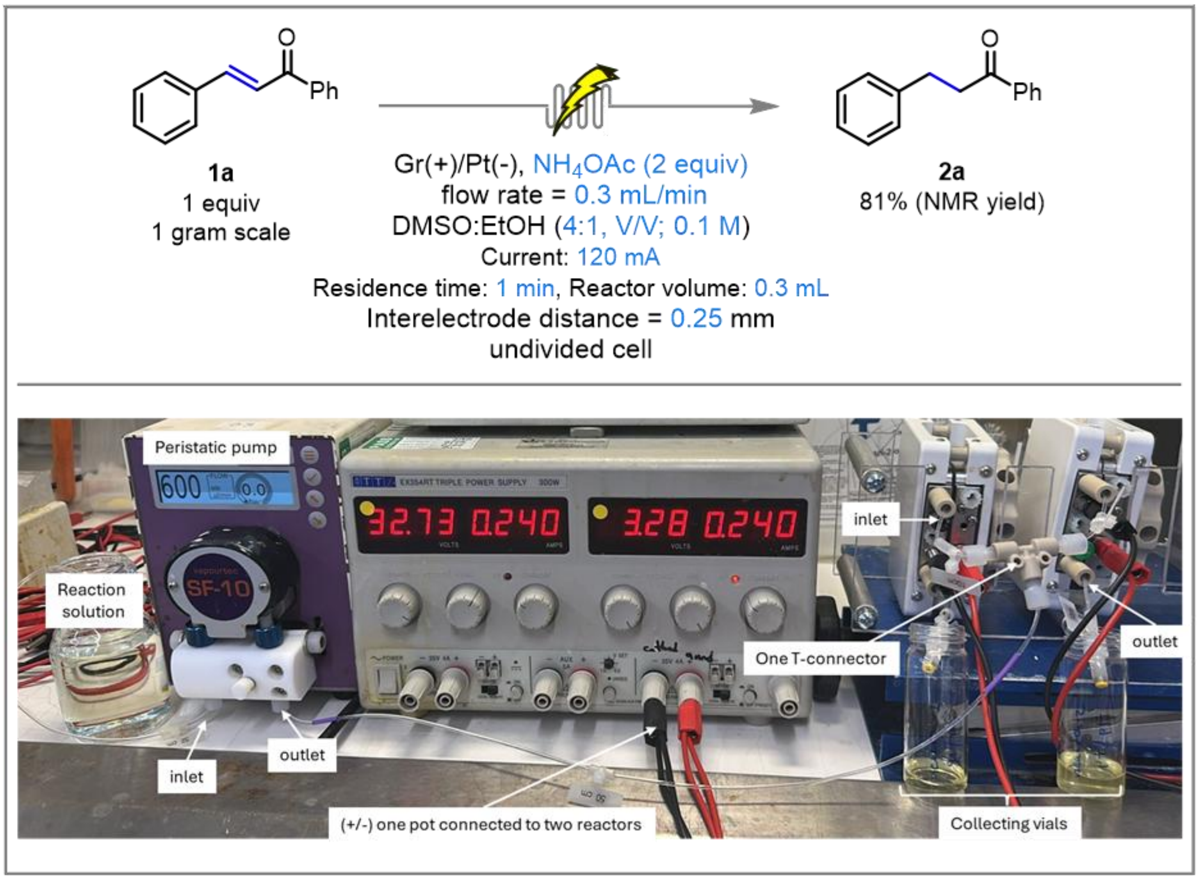

Figure S3: Ion-flow electrochemical set-up of two reactors in parallel configuration.

4-reactor setup: The reaction solution was introduced via the inlet of a peristaltic pump and directed into a primary Y- or T-shaped fluid connector. This junction split the stream into two downstream channels, each further connected to a secondary Y- or T-connector, which in turn distributed the flow to four independent reactor units, resulting in a total of four reactors operating in parallel. This cascaded branching design ensured uniform and simultaneous flow of the reaction mixture to all four reactors, minimizing residence time discrepancies and enhancing experimental reproducibility with increasing the productivity. Effluent from each reactor was collected separately in individual vials at the outlet of each reactor, as illustrated in Figure S4.

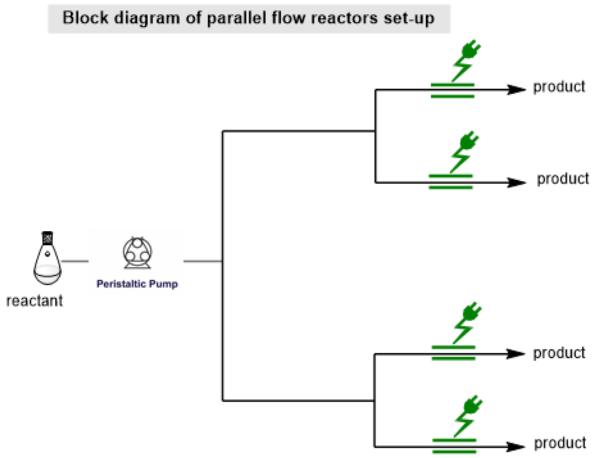

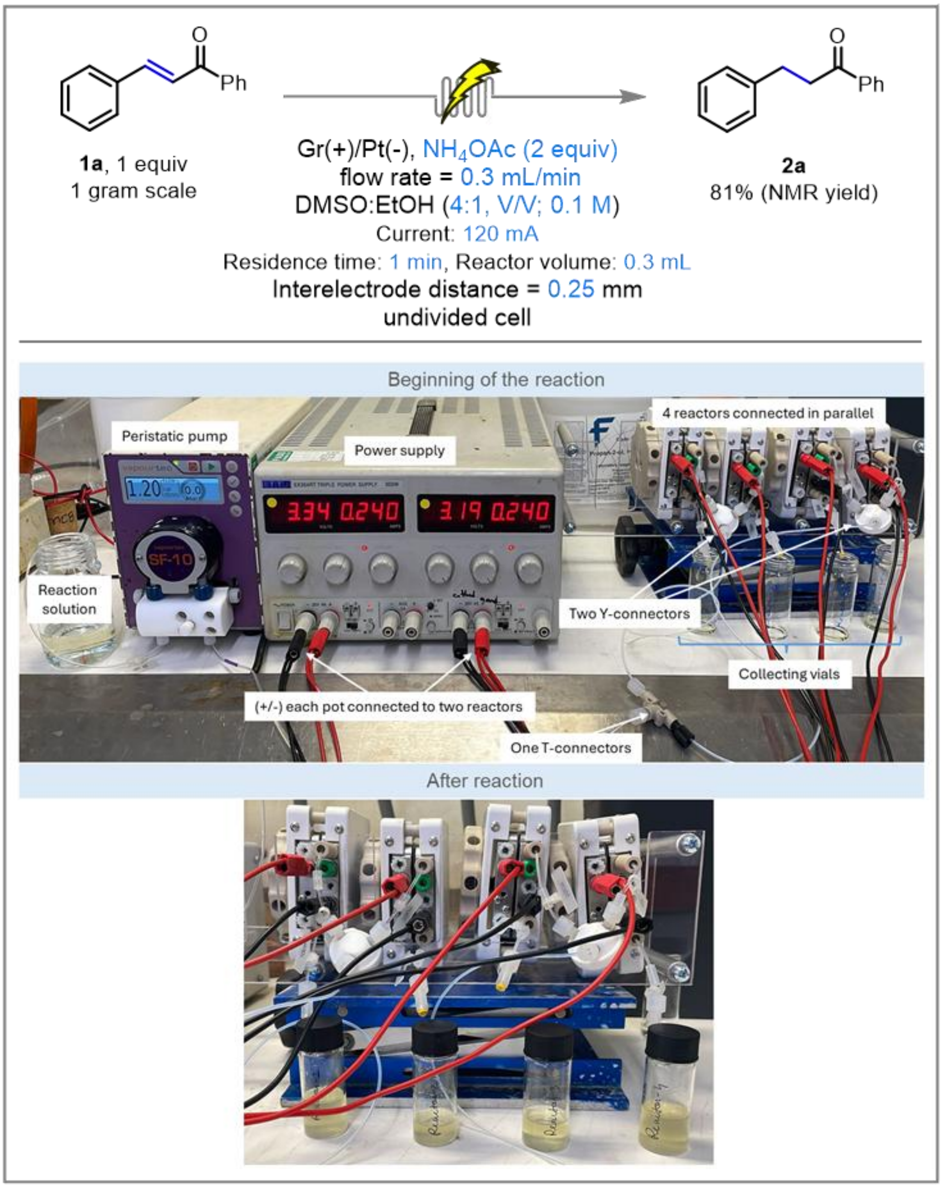

Figure S4: Ion-flow electrochemical set-up of four reactors in parallel configuration.

Electrochemical parameters in parallel configuration:

Flow rate:

As the flow stream was split using Y- or T-connectors, the volumetric flow was divided at each junction. Therefore, the peristaltic pump was set to deliver a total flow equal to twice the desired reaction flow rate for the two-reactor setup and four times the desired reaction flow rate for the four-reactor setup, so that each reactor receives the desired flow rate.

Current:

Electrolysis was performed under constant-current conditions using an Aim-TTi Digital Bench Power Supply (280 W, 2 outputs, 0–35 V, 0–4 A). One channel of the power supply was connected to the two-reactor array; therefore, the current set on the power supply was twice the intended current per reactor so that each reactor received the desired reaction current.

4.1.1.2. series configuration

Reaction protocol: Same as stated for the parallel configuration.

Connectivity of reactors in series configuration:

2-reactor setup: A dual-reactor configuration was employed to enable series processing of the reaction mixture under identical flow conditions. The reaction solution was pumped through a peristaltic pump whose outlet is connected to the inlet tubing of 1st reactor followed by the passing of reaction solution through 1st reactor which leads to the partial conversion of starting material, so the outlet of 1st reactor connected to the inlet of 2nd reactor to achieve the full conversion. This configuration allowed to achieve the complete conversion of the starting materials particularly for *α,β*- unsaturated esters and aryl alkynes, and the effluent collected in vial at the outlet of 2nd reactor, as shown in Figure S5.

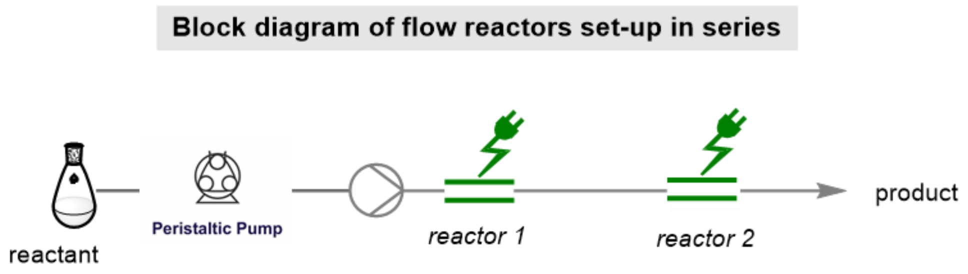

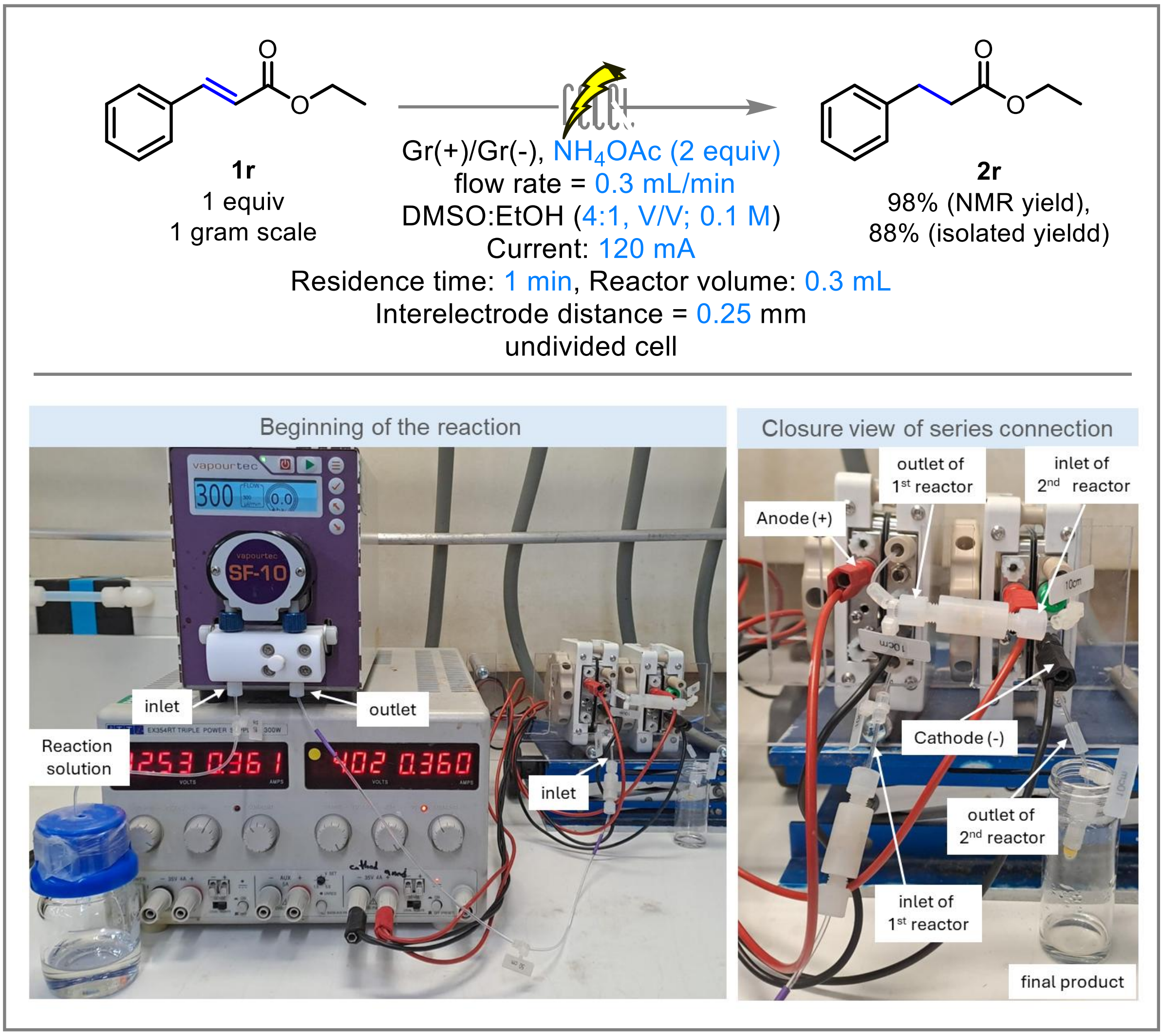

Figure S5: Ion-flow electrochemical set-up of two reactors in series configuration.

Electrochemical parameters in series configuration:

Flow rate:

The peristaltic pump was set to deliver the same flow rate as the desired reaction flow rate for 2 reactor set-up whereas, for 4-reactor set-up the pump was set to deliver 2x the reaction flow rate.

Current:

Electrolysis was performed under constant-current conditions using an Aim-TTi Digital Bench Power Supply (280 W, 2 outputs, 0–35 V, 0–4 A). One channel of the power supply was connected to the two-reactor array; therefore, the current set on the power supply was twice the intended current per reactor so that each reactor received the desired reaction current.

4.2. General flow-electrolysis protocol for the reduction of *α,β*- unsaturated aryl/hetero aryl ketones (GP4)

Note: All substrates were explored on a 2-gram scale otherwise stated, and all the solutions are prepared in 5 ml excess for the initial equilibrium of the reactors.

4.2.1. Method B:

GP4 is same as **GP3** except solvent system, (DMF: EtOH; 1:1; v/v; 0.1 M) used as desired solvent system.

5. General Flow-electrolysis Protocol for Scale-Up

5.1. 10 g scale using 4-reactor set-up in parallel configuration for reduction of chalcone:

Reaction protocol: Reaction performed at 10 gram scale in parallel configuration, chalcone (**1a**) as model substrate (0.1 M, 1 equiv, 48 mmol, 10 g), solvent: DMSO:EtOH, NH_4OAc (0.1 M, 2 equiv, 96 mmol, 7.50 g), retention time 1 min, overall reaction time 6 hr 40 min, conv.: 92%, NMR yield: 81%, isolated yield: 7.92 g, 38 mmol, 79%, productivity: 1.20 g/h (Figure S6).

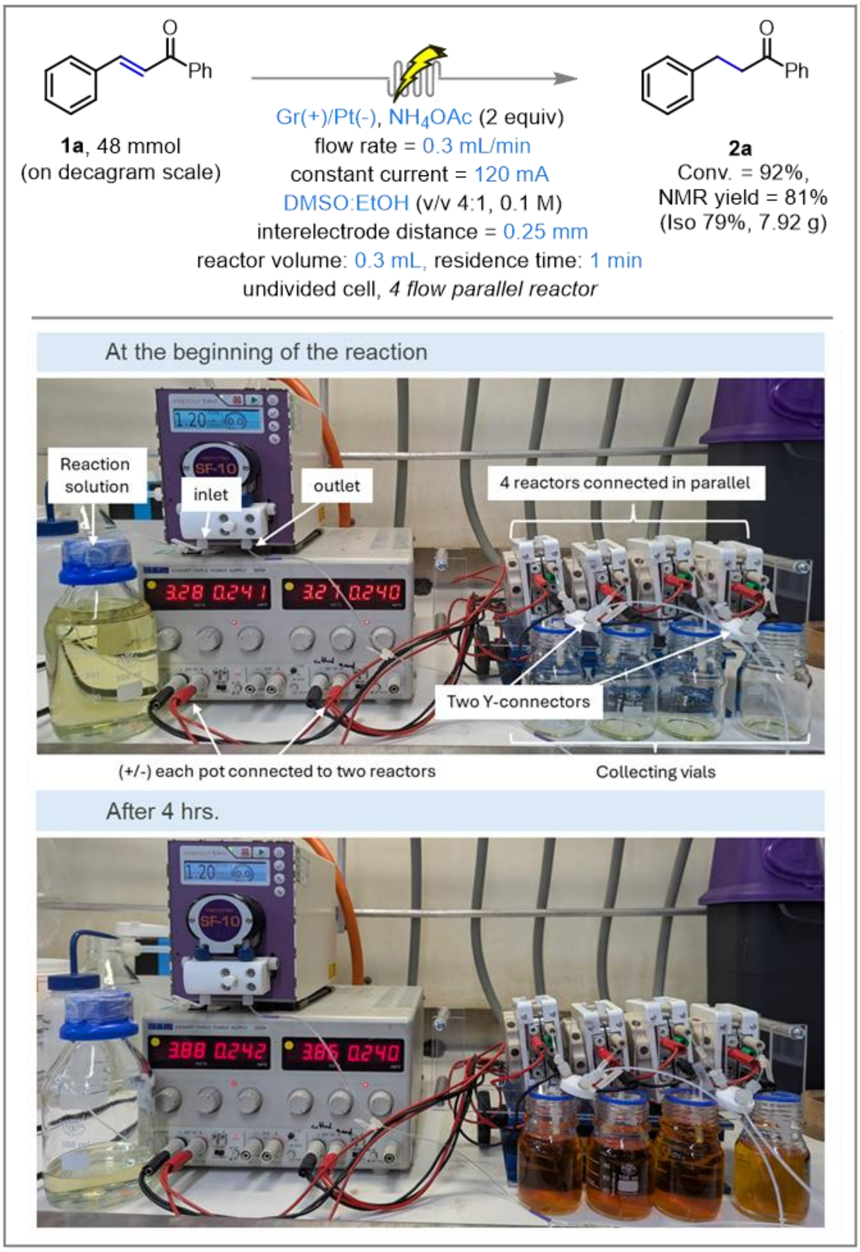

Figure S6: Ion-flow electrochemical set-up demonstrating scalable synthesis (10 gram) using four reactors in parallel configuration.

6. Cost Efficient Electrochemical Reactor Design: A Modified Single-Unit Approach for Dual-Reactor Operation

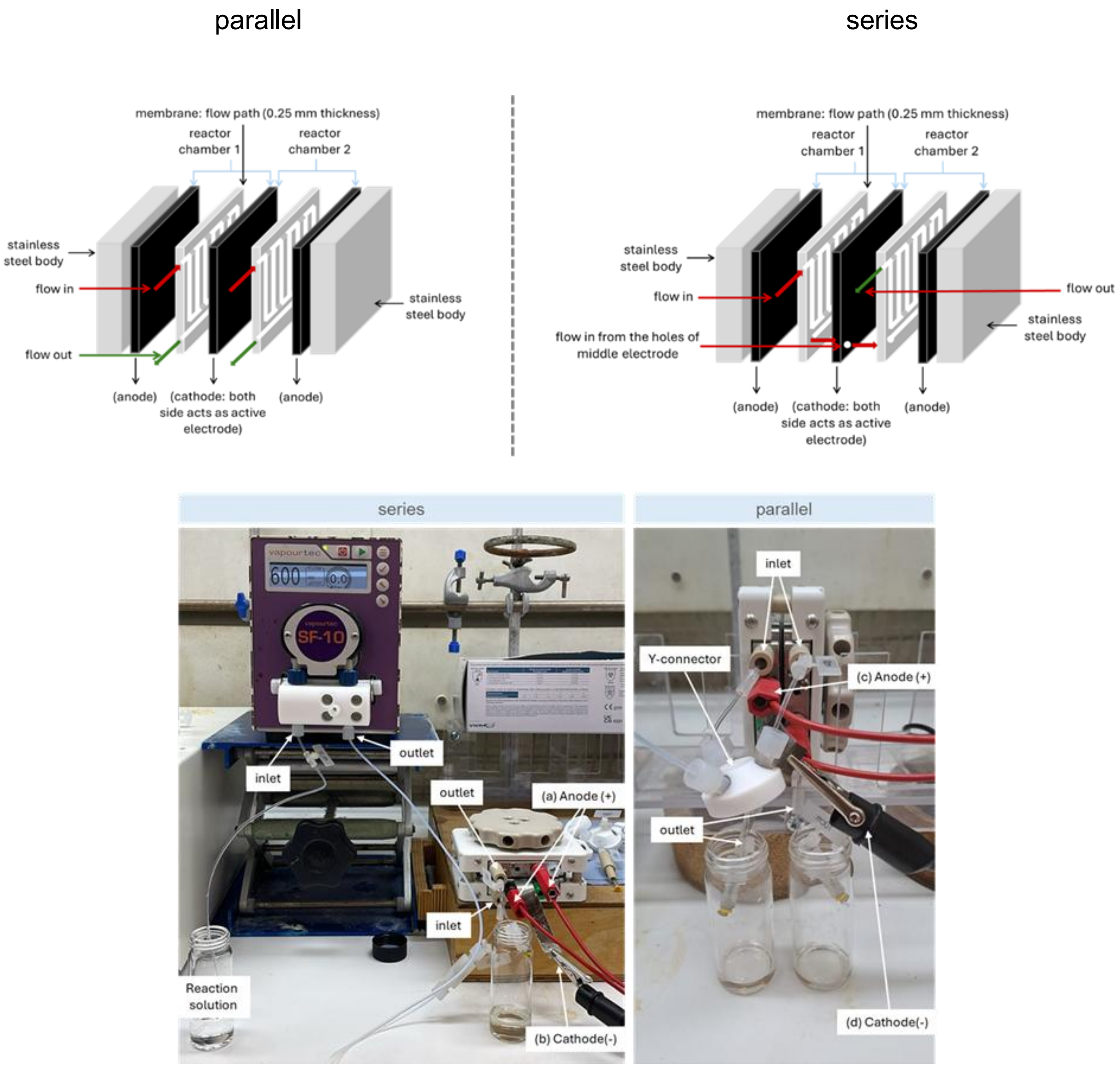

Figure S7: Modified Single-Unit Dual-Reactor Design: (upper) Schematic representation of parallel (left) and series (right) and (lower) reactor set-up in series (left) and parallel (right) operational mode.

7. Deuterium experiments

Deuterium experiments for α,β-unsaturated substrates:

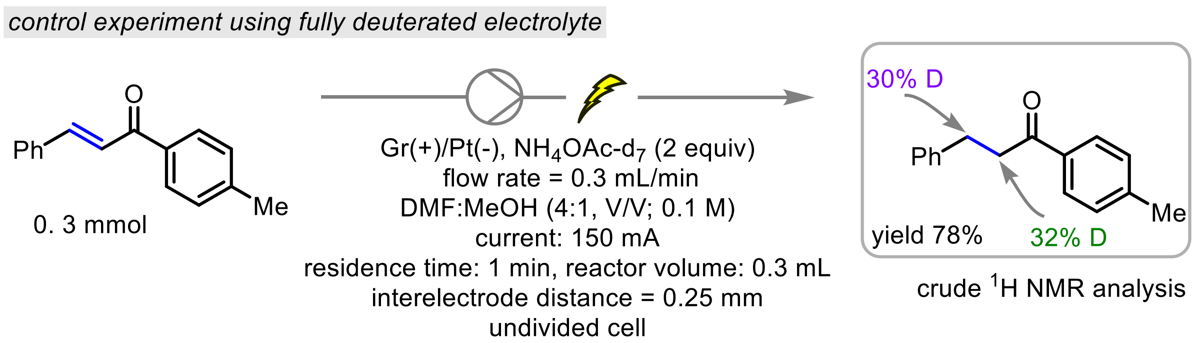

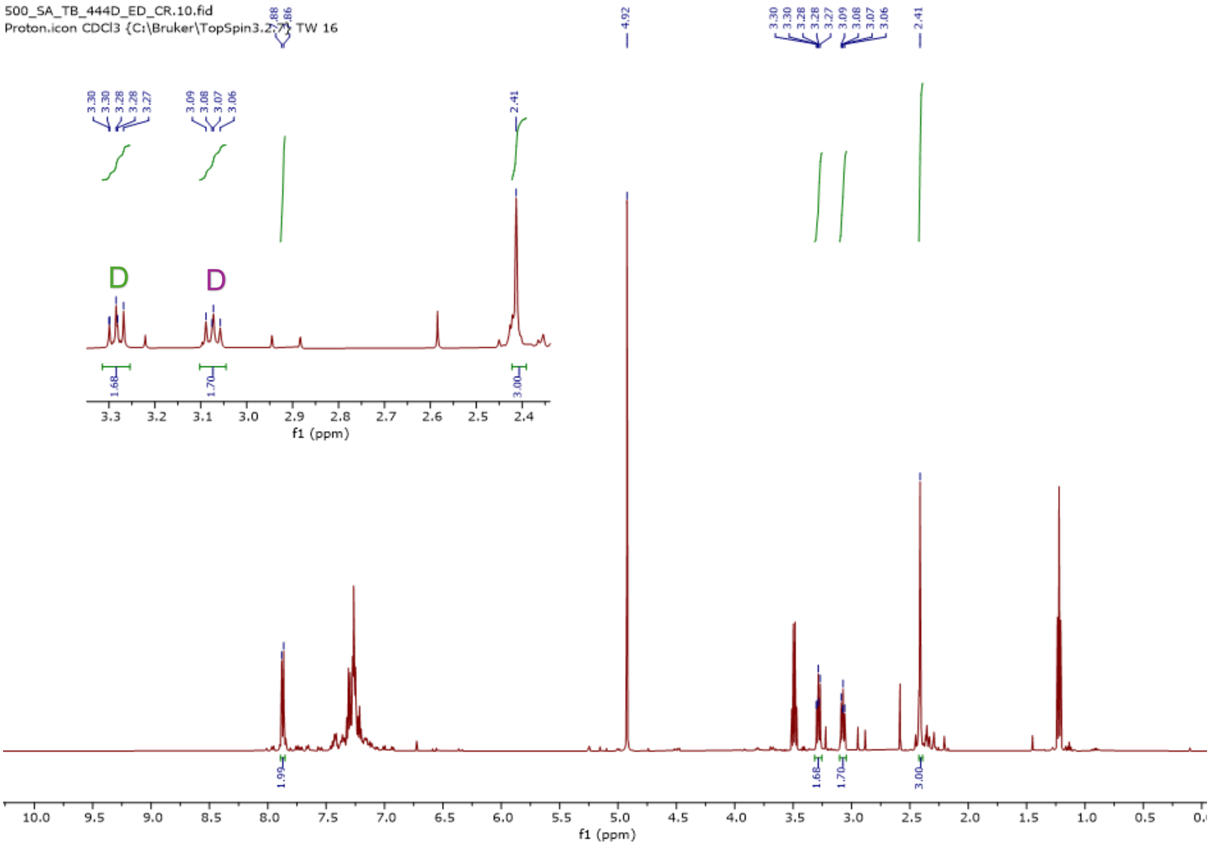

Control Experiment Using Fully Deuterated Solvents and Electrolyte

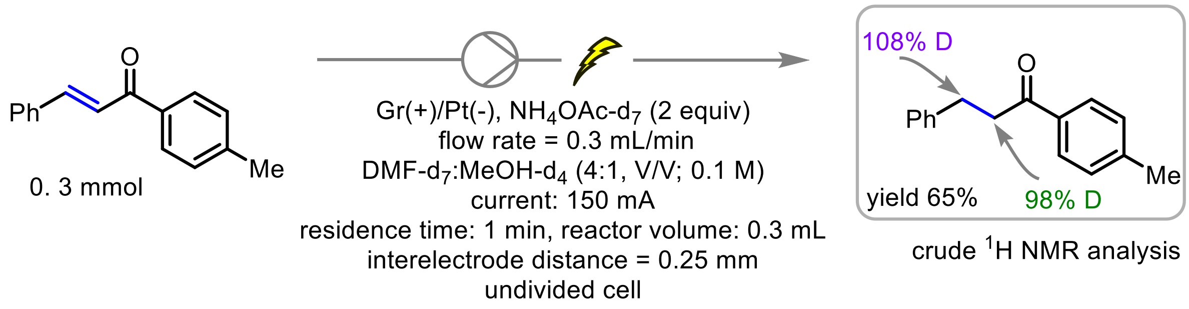

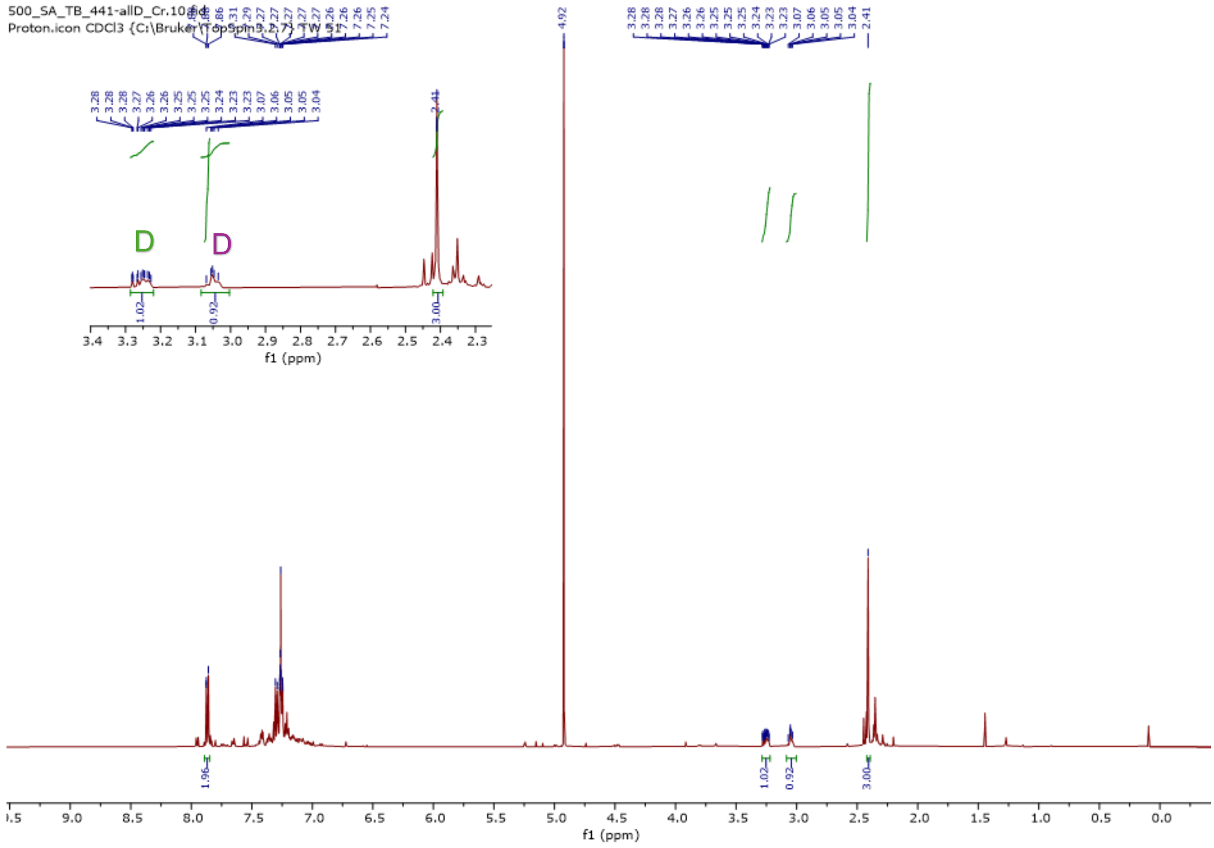

Control Experiment Using Fully Deuterated Solvents

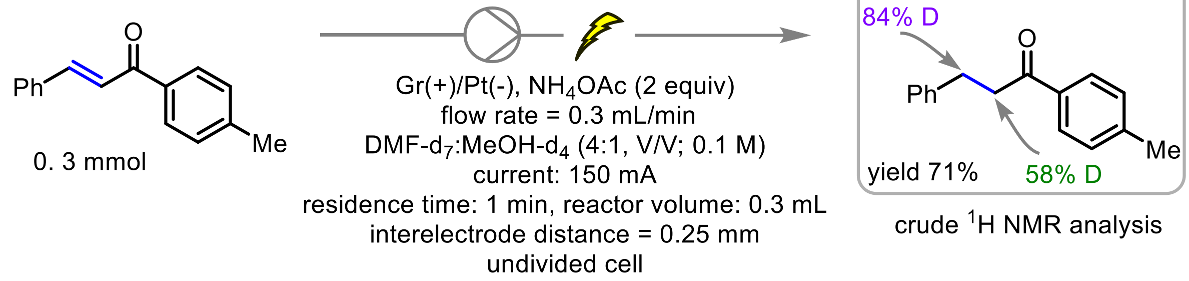

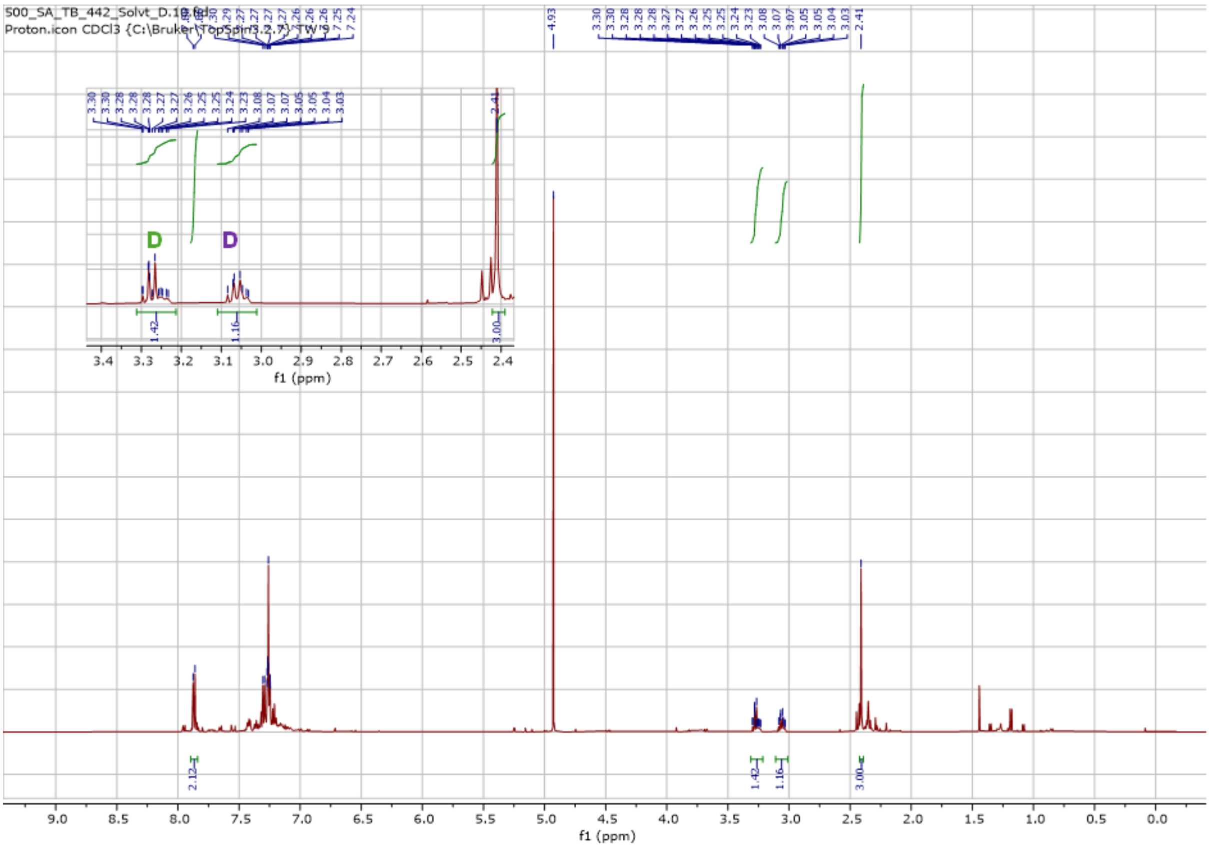

Table S3: Faradaic efficiency and F/mol of products

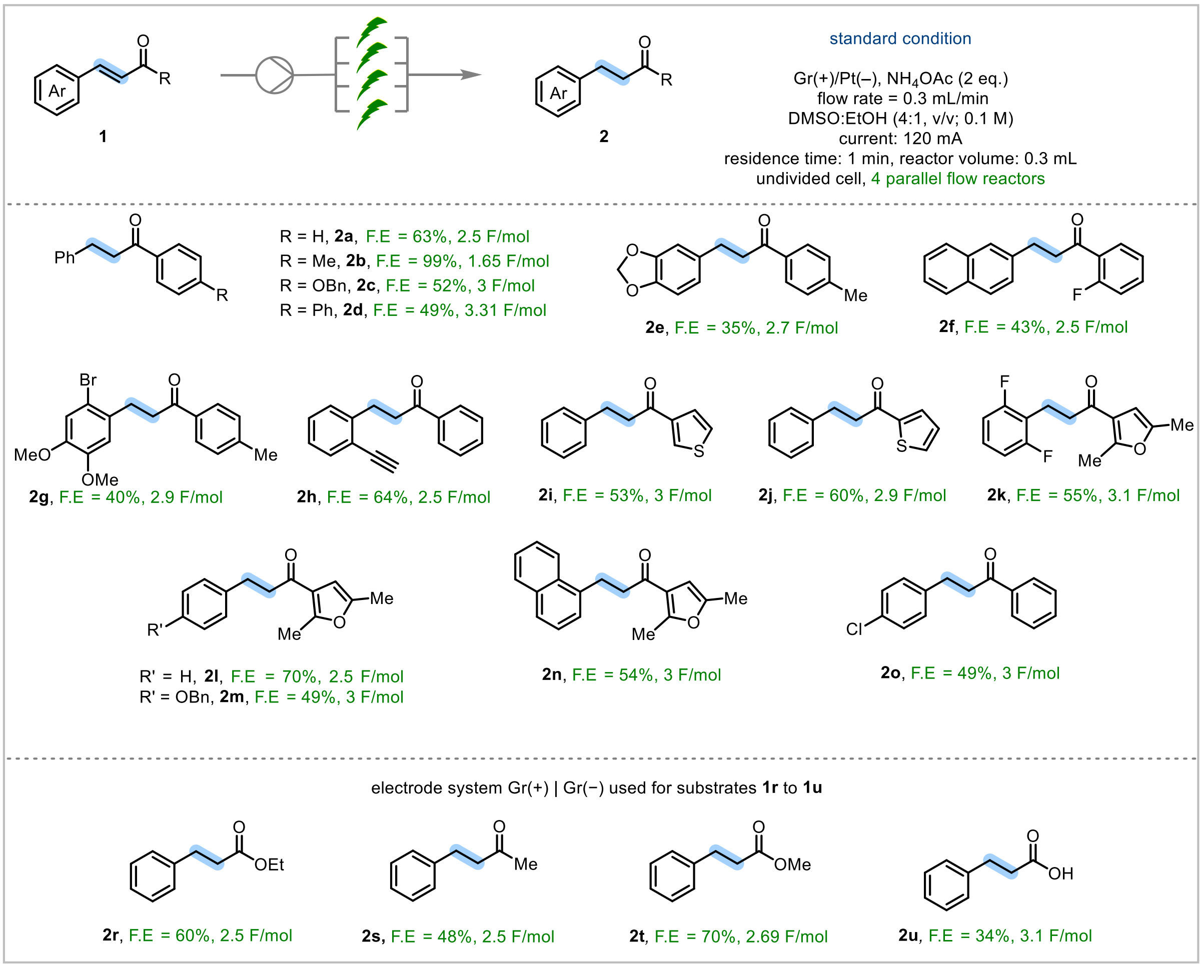

8. Details of reactor and electrodes cleaning

Stepwise procedure for electrode cleaning

Before reaction:

Step1: sanded the electrode surface (graphite, platinum) with 1200/800 grade sandpaper on both sides.

Step2: sonicate them in water, followed by methanol and then acetone for 10 mins each.

Step3: wiped the electrode surface with dry tissues and dried them in oven.

After reaction:

Step1: as the electrodes are wet in this case, rinsed them with methanol, followed by wiped with tissues and then follow the same steps as stated above for before reaction.

Reactor cleaning procedure:

Step1: After the completion of the reaction, pumped methanol (approx. 15 mL) through the reactor at a higher flow rate (note: without power) which ensures to clean the tubing and dis-contaminated the flow path inside of the reactor.

Step2: Dismantle the reactor followed by removal of both inlet and outlet and pumped methanol through each tubing with a syringe to ensure no blockage.
Step3: Wiped the body of the reactor with tissues dipped in methanol.

9. NMR spectral data

9.1. Characterization data for starting materials

(*E*)-1-(4-(Benzyloxy)phenyl)-3-phenylprop-2-en-1-one (S1)

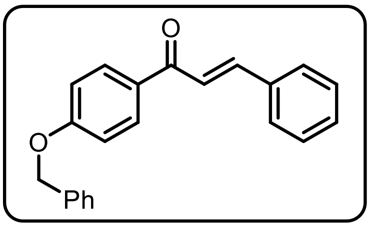

Compound **S1** was prepared according to the general procedure **GP1** using benzaldehyde (2 mL, 20 mmol) and 1-(4-(benzyloxy)phenyl)ethan-1-one (4.5 g, 20 mmol), affording off-white solid (5.20 g, 16.6 mmol, 83%).

^{1}H NMR (400 MHz, CDCl$_3$) δ 8.07 – 8.03 (m, 2H), 7.81 (d, *J* = 15.7 Hz, 1H), 7.67 – 7.63 (m, 2H), 7.55 (d, *J* = 15.7 Hz, 1H), 7.47 – 7.39 (m, 8H), 7.09 – 7.05 (m, 2H), 5.16 (s, 2H) ppm.
^{13}C NMR (101 MHz, CDCl$_3$) δ 188.8, 162.7, 144.2, 136.3, 135.2, 131.4, 131.0, 130.5, 129.1, 128.8, 128.5, 128.4, 127.6, 122.0, 114.8, 70.3 ppm.
The analytical data are in accordance with reported literature.[2]

(*E*)-3-Phenyl-1-(*p*-tolyl)prop-2-en-1-one (S2)

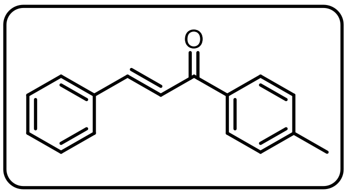

Compound **S2** was prepared according to the general procedure **GP1** using benzaldehyde (2 mL, 20 mmol) and 1-(p-tolyl)ethan-1-one (2.68 g, 20 mmol), affording a white solid (3.46 g, 15.6 mmol, 78%).
^{1}H NMR (400 MHz, CDCl$_3$) δ 8.00 – 7.95 (m, 2H), 7.84 (d, *J* = 15.7 Hz, 1H), 7.69 – 7.64 (m, 2H), 7.57 (d, *J* = 15.7 Hz, 1H), 7.43 (m, 3H), 7.32 (m, 2H), 2.45 (s, 3H) ppm.
^{13}C NMR (101 MHz, CDCl$_3$) δ 190.0, 144.4, 143.7, 135.6, 135.0, 130.5, 129.4, 129.0, 128.7, 128.5, 122.1, 21.7 ppm.

(*E*)-1-([1,1'-Biphenyl]-4-yl)-3-phenylprop-2-en-1-one (S3)

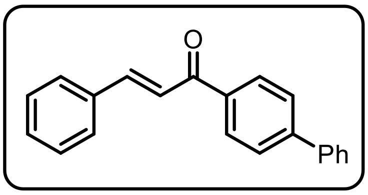

Compound **S3** was prepared according to the general procedure **GP1** using benzaldehyde (2 mL, 20 mmol) and 1-([1,1'-biphenyl]-4-yl)ethan-1-one (3.9 g, 20 mmol), affording a off-white solid (4.40 g, 15.5 mmol, 88%).
^{1}H NMR (400 MHz, CDCl$_3$) δ 8.16 – 8.09 (m, 2H), 7.86 (d, *J* = 15.7 Hz, 1H), 7.77 – 7.72 (m, 2H), 7.70 – 7.64 (m, 4H), 7.60 (d, *J* = 15.7 Hz, 1H), 7.54 – 7.36 (m, 6H) ppm.

^{13}C NMR (101 MHz, CDCl$_3$) δ 190.1, 145.7, 144.9, 140.1, 137.0, 135.0, 130.7, 129.3, 129.1, 129.1, 128.6, 128.4, 127.4, 125.9, 122.1 ppm.

(E)-1-(2-Fluorophenyl)-3-(naphthalen-2-yl)prop-2-en-1-one (S4)

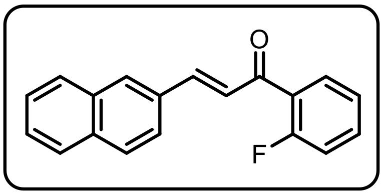

Compound **S4** was prepared according to the general procedure **GP1** using 2-naphthaldehyde (3.12 g, 20 mmol) and 1-(2-fluorophenyl)ethan-1-one (2.76 g, 20 mmol), affording a white solid (4.70 g, 17.0 mmol, 85%).

^{1}H NMR (400 MHz, CDCl$_3$) δ 8.01 (m, 1H), 7.90 – 7.82 (m, 5H), 7.77 (dd, *J* = 8.6, 1.7 Hz, 1H), 7.56 – 7.49 (m, 4H), 7.28 (m, 1H), 7.19 (ddd, *J* = 10.9, 8.3, 1.1 Hz, 1H) ppm.

^{13}C NMR (101 MHz, CDCl$_3$) δ 189.2 (d, *J* = 2.6 Hz), 161.3 (d, *J* = 253.1 Hz), 145.1 (d, *J* = 1.3 Hz), 134.6, 134.0 (d, *J* = 8.8 Hz), 133.4, 132.3, 131.1 (d, *J* = 2.7 Hz), 131.1, 128.8 (d, *J* = 7.3 Hz), 127.9, 127.6, 127.4, 127.3, 126.9, 125.9 (d, *J* = 6.6 Hz), 124.7 (d, *J* = 3.4 Hz), 123.8, 116.7 (d, *J* = 23.2 Hz) ppm.

^{19}F NMR (376 MHz, CDCl$_3$) δ –110.7 (s, 1F) ppm.

(*E*)-3-(2-Bromo-4,5-dimethoxyphenyl)-1-(*p*-tolyl)prop-2-en-1-one (S5)

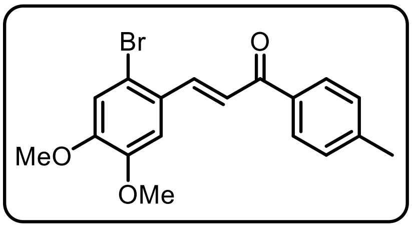

Compound **S5** was prepared according to the general procedure **GP1** using 2-bromo-4,5-dimethoxybenzaldehyde (4.9 g, 20 mmol) and 1-(p-tolyl)ethan-1-one (2.68 g, 20 mmol), affording a white solid (6.21 g, 17.2 mmol, 86%).

^{1}H NMR (400 MHz, CDCl$_3$) δ 8.05 (d, *J* = 15.7 Hz, 1H), 7.94 – 7.88 (m, 2H), 7.32 – 7.26 (m, 3H), 7.12 (d, *J* = 46.3 Hz, 2H), 3.94 (s, 3H), 3.90 (s, 3H), 2.42 (s, 3H) ppm.

^{13}C NMR (101 MHz, CDCl$_3$) δ 190.4, 151.5, 148.7, 143.7, 143.1, 135.6, 129.4, 128.9, 127.1, 123.1, 118.1, 115.8, 109.5, 56.4, 56.3, 21.8 ppm.

(*E*)-3-(Benzo[*d*][1,3]dioxol-5-yl)-1-(*p*-tolyl)prop-2-en-1-one (S6)

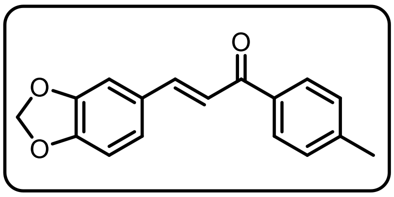

Compound **S6** was prepared according to the general procedure **GP1** using benzo[d][1,3]dioxole-5-carbaldehyde (3 g, 20 mmol) and 1-(p-tolyl)ethan-1-one (2.68 g, 20 mmol), affording a white solid (4.68 g, 17.6 mmol, 88%).

^{1}H NMR (400 MHz, $CDCl_3$) δ 7.94 – 7.90 (m, 2H), 7.73 (d, *J* = 15.5 Hz, 1H), 7.37 (d, *J* = 15.6 Hz, 1H), 7.29 (m, 2H), 7.18 – 7.16 (m, 1H), 7.12 (m, 1H), 6.84 (d, *J* = 8.0 Hz, 1H), 6.02 (s, 2H), 2.43 (s, 3H) ppm.

^{13}C NMR (101 MHz, $CDCl_3$) δ 190.0, 149.9, 148.5, 144.4, 143.6, 135.9, 129.6, 129.4, 128.7, 125.3, 120.2, 108.8, 106.8, 101.8, 21.8 ppm.

(E)-3-(2-Ethynylphenyl)-1-(p-tolyl)prop-2-en-1-one (S7)

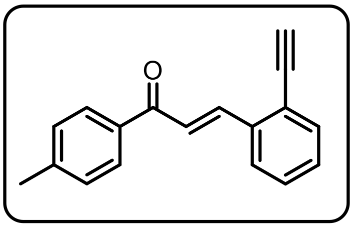

Compound **S7** was prepared according to the general procedure **GP1** using 2-ethynylbenzaldehyde (1 g, 7.69 mmol) and 1-(p-tolyl)ethan-1-one (1 g, 7.69 mmol), affording a brown solid (1.42 g, 5.76 mmol, 75%).

^{1}H NMR (400 MHz, $CDCl_3$) δ 8.26 (d, *J* = 15.8 Hz, 1H), 7.98 – 7.91 (m, 2H), 7.76 (dd, *J* = 7.8, 1.5 Hz, 1H), 7.62 (d, *J* = 15.8 Hz, 1H), 7.57 (dd, *J* = 7.7, 1.4 Hz, 1H), 7.43 – 7.38 (m, 1H), 7.35 (m, 1H), 7.31 (m, 2H), 3.44 (s, 1H), 2.44 (s, 3H) ppm.

^{13}C NMR (101 MHz, $CDCl_3$) δ 190.3, 143.9, 142.0, 137.2, 135.6, 133.8, 129.9, 129.5, 129.2, 128.9, 126.6, 124.3, 123.3, 83.7, 81.4, 21.8 ppm.

(*E*)-3-phenyl-1-(thiophen-2-yl)prop-2-en-1-one (S8)

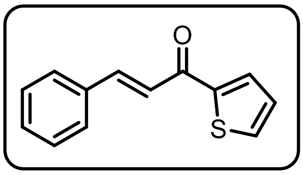

Compound **S8** was prepared according to the general procedure **GP1** using benzaldehyde (2.12 g, 20 mmol) and 1-(thiophen-2-yl)ethan-1-one (2.52 g, 20 mmol). The precipitate was filtered out, affording a brown solid (4.09 g, 19.07 mmol, 95%).

^{1}H NMR (400 MHz, $CDCl_3$) δ 7.91 – 7.81 (m, 2H), 7.68 (dd, *J* = 4.9, 1.1 Hz, 1H), 7.66 – 7.62 (m, 2H), 7.46 – 7.40 (m, 4H), 7.19 (dd, *J* = 4.9, 3.8 Hz, 1H) ppm.

^{13}C NMR (101 MHz, $CDCl_3$) δ 182.2, 145.6, 144.2, 134.8, 134.1, 132.0, 130.7, 129.1, 128.6, 128.4, 121.7 ppm.

(*E*)-3-Phenyl-1-(thiophen-3-yl)prop-2-en-1-one (S9)

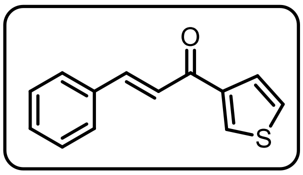

Compound **S9** was prepared according to the general procedure **GP1** using benzaldehyde (2.12 g, 20 mmol) and 1-(thiophen-3-yl)ethan-1-one (2.52 g, 20 mmol). The precipitate was filtered out, affording a beige solid (3.27 g, 15.24 mmol, 76%).

^{1}H NMR (400 MHz, CDCl$_3$) δ 8.17 (dd, *J* = 2.9, 1.3 Hz, 1H), 7.82 (d, *J* = 15.7 Hz, 1H), 7.68 (dd, *J* = 5.1, 1.3 Hz, 1H), 7.65 – 7.61 (m, 2H), 7.43 – 7.39 (m, 4H), 7.36 (dd, *J* = 5.1, 2.9 Hz, 1H) ppm.

^{13}C NMR (101 MHz, CDCl$_3$) δ 184.0, 144.2, 143.2, 134.9, 132.2, 130.6, 129.0, 128.5, 127.5, 126.6, 122.8 ppm.

The analytical data are in accordance with reported literature.[3]

(E)-1-(2,5-Dimethylfuran-3-yl)-3-(naphthalen-1-yl)prop-2-en-1-one (S10)

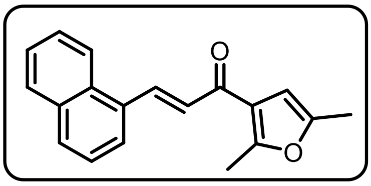

Compound **S10** was prepared according to the general procedure **GP1** using 1-naphthaldehyde (3.12 g, 20 mmol) and 1-(2,5-dimethylfuran-3-yl)ethan-1-one (2.76 g, 20 mmol). The precipitate was filtered out, affording a bright yellow solid (4.34 g, 15.70 mmol, 79%).

^{1}H NMR (400 MHz, CDCl$_3$) δ 8.58 (d, *J* = 15.4 Hz, 1H), 8.31 – 8.22 (m, 1H), 7.96 – 7.82 (m, 3H), 7.61 – 7.48 (m, 3H), 7.28 (m, 1H), 6.37 (s, 1H), 2.66 (s, 3H), 2.31 (s, 3H) ppm.

^{13}C NMR (101 MHz, CDCl$_3$) δ 185.9, 158.3, 150.3, 139.9, 133.9, 132.7, 131.9, 130.7, 128.9, 127.0, 126.4, 125.6, 125.0, 123.7, 122.6, 105.9, 77.4, 14.7, 13.4 ppm.

(*E*)-3-(2,6-Difluorophenyl)-1-(2,5-dimethylfuran-3-yl)prop-2-en-1-one (S11)

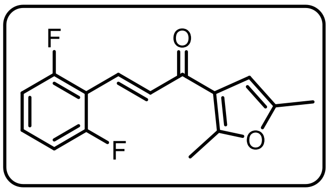

Compound **S11** was prepared according to the general procedure **GP1** using 2,6-difluorobenzaldehyde (2.84 g, 20 mmol) and 1-(2,5-dimethylfuran-3-yl)ethan-1-one (2.76 g, 20 mmol). The precipitate was filtered out, affording a white solid (1.80 g, 6.887 mmol, 34%).

^{1}H NMR (400 MHz, CDCl$_3$) δ 7.80 (d, *J* = 16.1 Hz, 1H), 7.48 (d, *J* = 16.1 Hz, 1H), 7.30 (m, 1H), 6.95 (t, *J* = 8.6 Hz, 2H), 6.32 (d, *J* = 1.2 Hz, 1H), 2.62 (s, 3H), 2.29 (s, 3H) ppm.

^{13}C NMR (101 MHz, CDCl$_3$) δ 186.0, 158.4, 150.3, 131.0 (t, *J* = 11.2 Hz), 129.9 (t, *J* = 11.2 Hz), 128.7, 122.7, 112.1, 111.9, 105.8, 77.4, 14.7, 13.4 ppm.

(*E*)-3-(4-(Benzyloxy)phenyl)-1-(2,5-dimethylfuran-3-yl)prop-2-en-1-one (S12)

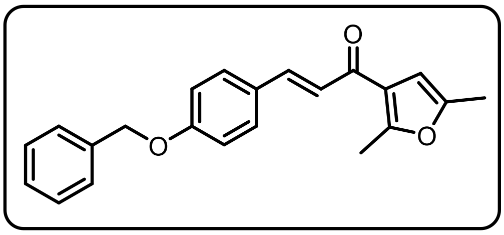

Compound **S12** was prepared according to the general procedure **GP1** using 4-(benzyloxy)benzaldehyde (4.25 g, 20 mmol) and 1-(2,5-dimethylfuran-3-yl)ethan-1-one (2.76 g, 20 mmol). The precipitate was filtered out, affording a pale yellow solid (4.2378 g, 12.75 mmol, 63.75%).

^{1}H NMR (400 MHz, CDCl$_3$) δ 7.69 (d, *J* = 15.7 Hz, 1H), 7.59 – 7.53 (m, 2H), 7.45 – 7.38 (m, 5H), 7.06 (d, *J* = 15.7 Hz, 1H), 6.99 (d, *J* = 8.8 Hz, 2H), 6.32 (s, 1H), 5.11 (s, 2H), 2.61 (s, 3H), 2.29 (s, 3H) ppm.

^{13}C NMR (101 MHz, CDCl$_3$) δ 186.1, 160.7, 157.8, 150.1, 142.6, 136.6, 130.2, 128.8, 128.3, 128.1, 127.6, 122.7, 122.2, 115.4, 105.9, 70.2, 14.6, 13.4 ppm.

(*E*)-1-(2,5-Dimethylfuran-3-yl)-3-phenylprop-2-en-1-one (S13)

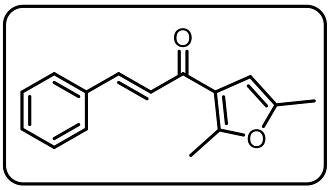

Compound **S13** was prepared according to the general procedure **GP1** using benzaldehyde (2.12 g, 20 mmol) and 1-(2,5-dimethylfuran-3-yl)ethan-1-one (2.76 g, 20 mmol). The precipitate was filtered out, affording a white solid (4 g, 17.6 mmol, 88%).

^{1}H NMR (400 MHz, CDCl$_3$) δ 7.72 (d, *J* = 16.1 Hz, 1H), 7.62 – 7.58 (m, 2H), 7.43 – 7.38 (m, 3H), 7.18 (d, *J* = 15.7 Hz, 1H), 6.34 (d, *J* = 1.2 Hz, 1H), 2.62 (s, 3H), 2.29 (s, 3H) ppm.

^{13}C NMR (101 MHz, CDCl$_3$) δ 186.1, 158.1, 150.2, 142.9, 135.1, 130.4, 129.0, 128.5, 124.3, 122.6, 105.8, 14.6, 13.4 ppm.

(E)-3-(4-chlorophenyl)-1-phenylprop-2-en-1-one (S14)

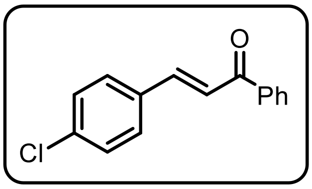

Compound **S14** was prepared according to the general procedure **GP1** using 4-chlorobenzaldehyde (2.80 g, 20 mmol) and acetophenone (2.40 g, 20 mmol). The precipitate was filtered out, affording a white solid (4 g, 16.4 mmol, 82%).

^{1}H NMR (500 MHz, CDCl$_3$) δ 8.04 – 7.99 (m, 2H), 7.76 (d, *J* = 15.7 Hz, 1H), 7.62 – 7.56 (m, 3H), 7.57 – 7.46 (m, 3H), 7.42 – 7.35 (m, 2H) ppm.

^{13}C NMR (126 MHz, CDCl$_3$) δ 190.4, 143.4, 138.1, 136.6, 133.5, 133.1, 129.7, 129.38, 128.8, 128.6, 122.6 ppm.

9.2. Characterization data for α,β-unsaturated ketone derivatives

1,3-Diphenylpropan-1-one (2a)

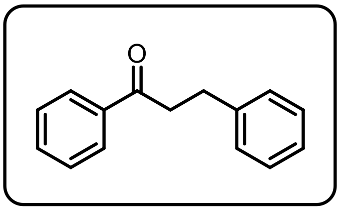

Compound **2a** was prepared according to the general procedure **GP4** from chalcone (10 g, 48 mmol, 0.1 M). The crude product was purified by flash column chromatography (EA/PE = 5:95), affording a white solid.

***e-flow* parameters:**

Configuration: 4 reactors in parallel

Electrode material: Gr(+) | Pt(−)

Current: 120 mA (each reactor)

Reaction flow rate: 0.3 mL/min (each reactor)

Residence time: 1 min

Total reaction time: 6 hr 40 min

Conv.: 92%, NMR yield: 78%, Isolated yield: 7.92 g, 38 mmol, 79%

Productivity: 1.20 g/h

Spectral details:

^{1}H NMR (500 MHz, $CDCl_3$) δ 7.95 – 7.90 (m, 2H), 7.53 – 7.49 (m, 1H), 7.43 – 7.39 (m, 2H), 7.28 – 7.24 (m, 2H), 7.23 – 7.20 (m, 2H), 7.18 – 7.15 (m, 1H), 3.26 (t, J = 8.0 Hz, 2H), 3.03 (t, J = 8.0 Hz, 2H) ppm.

^{13}C NMR (126 MHz, $CDCl_3$) δ 199.3, 141.4, 136.9, 133.2, 128.7, 128.7, 128.6, 128.2, 126.3, 40.6, 30.2 ppm.

HRMS (ESI): m/z calculated for [$C_{15}H_{14}O$] [M^+]: 210.1039, measured: 210.1030.

The analytical data are in accordance with reported literature.[1]

3-Phenyl-1-(p-tolyl)propan-1-one (2b)

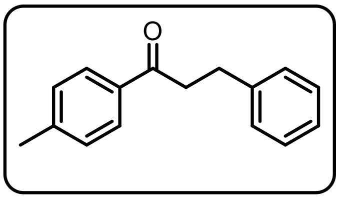

Compound **2b** was prepared according to the general procedure **GP4** from **S2** (2 g, 9.0 mmol, 0.2 M). The crude product was purified by flash column chromatography (EA/PE = 10:90), affording an off white solid.

***e-flow* parameters:**

Configuration: 4 reactors in parallel

Electrode material: Gr(+) | Pt(−)

Current: 160 mA (each reactor)

Reaction flow rate: 0.3 mL/min (each reactor)

Residence time: 1 min

Total reaction time: 38 min

Conv.: >95%, NMR yield: 82%, Isolated yield: 1.60 g, 7.11 mmol, 79%

Productivity: 2.52 g/h

Spectral details:

^{1}H NMR (400 MHz, CDCl$_3$) δ 7.91 – 7.88 (m, 2H), 7.35 – 7.24 (m, 7H), 3.33 – 3.29 (m, 2H), 3.09 (t, *J* = 7.9 Hz, 2H), 2.44 (s, 3H) ppm.

^{13}C NMR (101 MHz, CDCl$_3$) δ 199.1, 144.0, 141.6, 134.5, 129.5, 128.7, 128.6, 128.3, 126.3, 40.5, 30.4, 21.8 ppm.

HRMS (ESI): m/z calculated for [$C_{16}H_{16}O$] [M^+]: 224.1195, measured: 224.1191.

The analytical data are in accordance with reported literature.[1,4]

1-(4-(Benzyloxy)phenyl)-3-phenylpropan-1-one (2c)

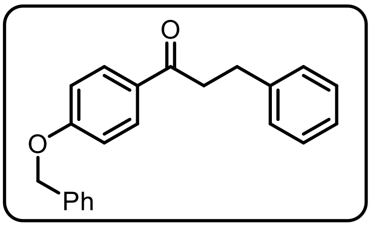

Compound **2c** was prepared according to the general procedure **GP4** from **S1** (2 g, 6.36 mmol, 0.1 M). The crude product was purified by flash column chromatography (EA/PE = 5:95), affording a colourless solid (1.63 g, 5.15 mmol, 80%).

***e-flow* parameters:**

Configuration: 4 reactors in parallel

Electrode material: Gr(+) | Pt(−)

Current: 150 mA (each reactor)

Reaction flow rate: 0.3 mL/min (each reactor)

Residence time: 1 min

Total reaction time: 54 min

Conv.: >87%, NMR yield: 81%, Isolated yield: 1.62 g, 5.15 mmol, 80%

Productivity: 1.80 g/h

Spectral details:

^{1}H NMR (400 MHz, CDCl$_3$) δ 7.94 (d, *J* = 8.9 Hz, 2H), 7.45 – 7.27 (m, 8H), 7.26 – 7.13 (m, 2H), 7.00 (d, *J* = 9.0 Hz, 2H), 5.13 (s, 2H), 3.25 (t, *J* = 12 Hz, 3H), 3.05 (t, *J* = 12 Hz, 3H) ppm.

^{13}C NMR (101 MHz, CDCl$_3$) δ 198.0, 162.7, 141.6, 136.3, 130.5, 130.3, 128.9, 128.7, 128.6, 128.4, 127.6, 126.2, 114.7, 70.3, 40.3, 30.5 ppm.

The analytical data are in accordance with reported literature.[5]

1-([1,1'-Biphenyl]-4-yl)-3-phenylpropan-1-one (2d)

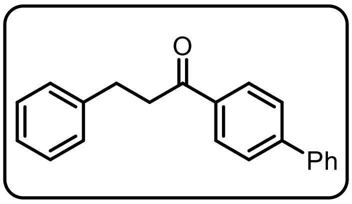

Compound **2d** was prepared according to the general procedure **GP4** from **S3** (2 g, 7 mmol, 0.05 M). The crude product was purified by flash column chromatography (EA/PE = 0.5:99.5), affording an off white solid.

***e-flow* parameters:**

Configuration: 4 reactors in parallel

Electrode material: Gr(+) | Pt(−)

Current: 80 mA (each reactor)

Reaction flow rate: 0.3 mL/min (each reactor)

Residence time: 1 min

Total reaction time: 117 min (1 hr 57 min)

Conv.: not resolved, NMR yield: 85%, Isolated yield: 1.68 g, 5.88 mmol, 84%

Productivity: 0.84 g/h

Spectral details:

^{1}H NMR (400 MHz, CDCl$_3$) δ 8.06 – 8.01 (m, 2H), 7.70 – 7.61 (m, 4H), 7.50 – 7.45 (m, 2H), 7.43 – 7.38 (m, 1H), 7.34 – 7.27 (m, 4H), 7.24 – 7.20 (m, 1H), 3.34 (t, *J* = 8 Hz, 2H), 3.10 (t, *J* = 8 Hz, 2H) ppm.

^{13}C NMR (101 MHz, CDCl$_3$) δ 199.0, 145.9, 141.4, 140.0, 135.7, 129.3, 129.1, 128.8, 128.7, 128.6, 128.4, 127.4, 126.3, 40.7, 30.3 ppm.

The analytical data are in accordance with reported literature.[6]

3-(Benzo[*d*][1,3]dioxol-5-yl)-1-(*p*-tolyl)propan-1-one (2e)

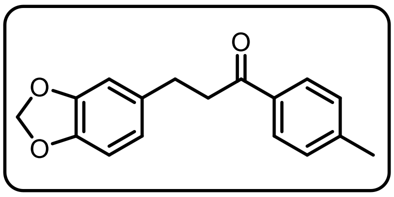

Compound **2e** was prepared according to the general procedure **GP4** from **S6** (2 g, 7.51 mmol, 0.1 M). The crude product was purified by flash column chromatography (EA/PE = 0.5:99.5), affording a yellow oil.

***e-flow* parameters:**

Configuration: 4 reactors in parallel

Electrode material: Gr(+) | Pt(−)

Current: 130 mA (each reactor)

Reaction flow rate: 0.3 mL/min (each reactor)

Residence time: 1 min

Total reaction time: 60 min

Conv.: >88%, NMR yield: 42%, Isolated yield: 0.84 g, 3.08 mmol, 41%

Productivity: 0.84 g/h

Spectral details:

^{1}H NMR (400 MHz, $CDCl_3$) δ 7.88 (d, *J* = 8.2 Hz, 2H), 7.27 (d, *J* = 7.9 Hz, 2H), 6.80 – 6.68 (m, 3H), 5.93 (s, 2H), 3.28 – 3.21 (t, *J* = 8 Hz, 2H), 3.00 (t, *J* = 8 Hz, 2H), 2.43 (s, 3H) ppm.

^{13}C NMR (101 MHz, $CDCl_3$) δ 198.9, 147.7, 145.8, 143.9, 135.2, 134.4, 129.3, 128.2, 121.2, 109.0, 108.3, 100.9, 77.5, 76.8, 40.6, 30.0, 21.7 ppm.

HRMS (ESI): m/z calculated for [$C_{17}H_{16}O_3$] [M^+]: 268.1094, measured: 268.1091.

Trace amount of inseparable impurities present.

The analytical data are in accordance with reported literature.[7]

1-(2-Fluorophenyl)-3-(naphthalen-2-yl)propan-1-one (2f)

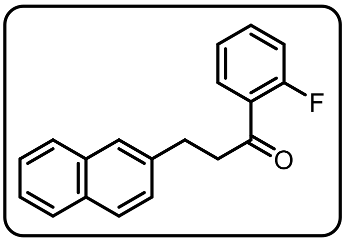

Compound **2f** was prepared according to the general procedure **GP4** from **S4** (2 g, 7.24 mmol, 0.1 M). The crude product was purified by flash column chromatography (EA/PE = 5:95), affording a yellow solid.

***e-flow* parameters:**

Configuration: 4 reactors in parallel

Electrode material: Gr(+) | Pt(−)

Current: 120 mA (each reactor)

Reaction flow rate: 0.3 mL/min (each reactor)

Residence time: 1 min

Total reaction time: 60 min

Conv.: >87%, NMR yield: 55%, Isolated yield: 1.07 g, 3.84 mmol, 53%

Productivity: 1.08 g/h

Spectral details:

^{1}H NMR (400 MHz, $CDCl_3$) δ 7.71 – 7.66 (m, 1H), 7.82 – 7.78 (m, 3H), 7.69 (s, 1H), 7.55 – 7.48 (m, 1H), 7.46 – 7.39 (m, 3H), 7.25 – 7.21 (m, 1H), 7.16 – 7.11 (m, 1H), 3.47 – 3.37 (m, 2H), 3.23 (t, *J* = 8.0 Hz, 2H) ppm.

^{13}C NMR (101 MHz, $CDCl_3$) δ 197.7 (d, *J* = 4.1 Hz), 162.1 (d, *J* = 254.5 Hz), 138.8, 134.69 (d, *J* = 9.0 Hz), 133.7, 132.2, 130.8 (d, *J* = 2.7 Hz), 128.2, 127.7, 127.6, 127.4, 126.7, 126.1, 125.7 (d, *J* = 13.0 Hz), 125.4, 124.6 (d, *J* = 3.4 Hz), 116.8 (d, *J* = 24.0 Hz), 45.3 (d, *J* = 7.3 Hz), 30.3 (d, *J* = 2.1 Hz) ppm.

^{19}F NMR (376 MHz, $CDCl_3$) δ –109.1 (s, 1F) ppm.

HRMS (ESI): m/z calculated for [$C_{19}H_{15}OF$] [M^+]: 278.1101, measured: 278.1096.

3-(2-Bromo-4,5-dimethoxyphenyl)-1-(*p*-tolyl)propan-1-one (2g)

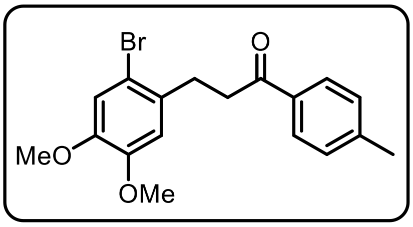

Compound **2g** was prepared according to the general procedure **GP4** from **S5** (2 g, 5.54 mmol, 0.1 M). The crude product was purified by flash column chromatography (EA/PE = 2:98), affording an off white solid.

***e-flow* parameters:**

Configuration: 4 reactors in parallel

Electrode material: Gr(+) | Pt(−)

Current: 140 mA (each reactor)

Reaction flow rate: 0.3 mL/min (each reactor)

Residence time: 1 min

Total reaction time: 47 min

Conv.: >87%, NMR yield: 60%, Isolated yield: 1.166 g, 3.21 mmol, 58%

Productivity: 1.50 g/h

Spectral details:

^{1}H NMR (400 MHz, CDCl$_3$) δ 7.87 (d, J = 8.2 Hz, 2H), 7.25 – 7.22 (m, 2H), 7.00 (s, 1H), 6.82 (s, 1H), 3.85 (d, J = 3.1 Hz, 6H), 3.35 (t, J = 8 Hz, 3H), 3.09 (t, J = 8 Hz, 3H), 2.41 (s, 2H) ppm.

^{13}C NMR (101 MHz, CDCl$_3$) δ 199.0, 148.5, 148.2, 144.1, 134.5, 132.7, 129.4, 128.4, 115.6, 114.0, 113.5, 56.3, 56.2, 39.0, 30.8, 21.8 ppm.

The analytical data are in accordance with reported literature.[8]

3-(2-Ethynylphenyl)-1-(*p*-tolyl)propan-1-one (2h)

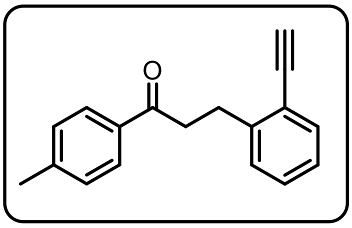

Compound **2h** was prepared according to the general procedure **GP4** from **S7** (0.5 g, 2.03 mmol, 0.1 M). The crude product was purified by flash column chromatography (EA/PE = 0.5:99.5), affording a yellow oil.

***e-flow* parameters:**

Configuration: 4 reactors in parallel

Electrode material: Gr(+) | Pt(−)

Current: 120 mA (each reactor)

Reaction flow rate: 0.3 mL/min (each reactor)

Residence time: 1 min

Total reaction time: 17 min

Conv.: >95%, NMR yield: 82%, Isolated yield: 0.41 g, 1.60 mmol, 79%

Productivity: 1.44 g/h

Spectral details:

^{1}H NMR (400 MHz, CDCl$_3$) δ 7.81 (d, J = 8.2 Hz, 2H), 7.42 (m, 1H), 7.25 – 7.20 (m, 2H), 7.19 – 7.15 (m, 2H), 7.15 – 7.06 (m, 1H), 3.27 – 3.21 (m, 2H), 3.20 (s, 1H), 3.18 – 3.11 (m, 2H), 2.33 (s, 3H) ppm.

^{13}C NMR (101 MHz, CDCl$_3$) δ 199.1, 144.2, 144.0, 134.5, 133.2, 129.4, 129.3, 129.2, 128.37, 126.3, 121.6, 82.3, 81.3, 39.4, 29.4, 21.8 ppm.

3-Phenyl-1-(thiophen-3-yl)propan-1-one (2i)

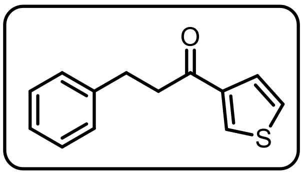

Compound **2i** was prepared according to the general procedure **GP4** from **S9** (2 g, 9.33 mmol, 0.1 M). The crude product was purified by flash column chromatography (EA/PE = 0.5:99.5), affording a colourless liquid.

***e-flow* parameters:**

Configuration: 4 reactors in parallel
Electrode material: Gr(+) | Pt(−)
Current: 150 mA (each reactor)
Reaction flow rate: 0.3 mL/min (each reactor)
Residence time: 1 min
Total reaction time: 78 min
Isolated yield: 1.66 g, 7.65 mmol, 82%
Productivity: 1.32 g/h

Spectral details:

^{1}H NMR (500 MHz, CDCl$_3$) δ 7.97 (dd, J = 2.9, 1.3 Hz, 1H), 7.50 (dd, J = 5.1, 1.3 Hz, 1H), 7.28 – 7.23 (m, 3H), 7.21 – 7.19 (m, 2H), 7.18 – 7.14 (m, 1H), 3.16 (t, 8 Hz, 2H), 3.01 (t, 8 Hz 2H) ppm.

^{13}C NMR (126 MHz, CDCl$_3$) δ 193.7, 142.3, 141.3, 132.0, 128.6, 128.5, 127.0, 126.5, 126.3, 41.8, 30.2 ppm.

The analytical data are in accordance with reported literature.[9]

3-Phenyl-1-(thiophen-2-yl)propan-1-one (2j)

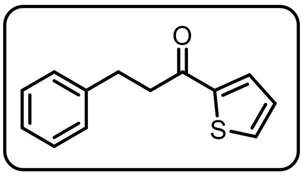

Compound **2j** was prepared according to the general procedure **GP4** from **S8** (0.5 g, 2.34 mmol, 0.1 M). The crude product was purified by flash column chromatography (EA/PE = 0.5:99.5), affording a colourless oil.

***e-flow* parameters:**

Configuration: 2 reactors in parallel
Electrode material: Gr(+) | Pt(−)

Current: 140 mA (each reactor)

Reaction flow rate: 0.3 mL/min (each reactor)

Residence time: 1 min

Total reaction time: 38 min

NMR yield: 90%, Isolated yield: 0.440 g, 2.03 mmol, 87%

Productivity: 0.72 g/h

Spectral details:

^{1}H NMR (400 MHz, $CDCl_3$) δ 7.69 (dd, *J* = 3.8, 1.1 Hz, 1H), 7.63 (dd, *J* = 5.0, 1.1 Hz, 1H), 7.34 – 7.26 (m, 3H), 7.25 – 7.18 (m, 2H), 7.13 – 7.09 (m, 1H), 3.24 (t, 8 Hz, 2H), 3.07 (t, 8 Hz, 2H) ppm.

^{13}C NMR (101 MHz, $CDCl_3$) δ 192.3, 144.3, 141.1, 133.7, 132.0, 128.7, 128.6, 128.2, 126.4, 41.3, 30.5 ppm.

The analytical data are in accordance with reported literature.[1]

3-(2,6-Difluorophenyl)-1-(2,5-dimethylfuran-3-yl)propan-1-one (2k)

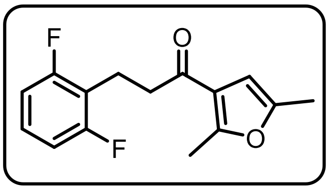

Compound **2k** was prepared according to the general procedure **GP4** from **S11** (0.5 g, 1.90 mmol, 0.1 M). The crude product was purified by flash column chromatography (EA/PE = 1:99), affording a colourless oil.

***e-flow* parameters:**

Configuration: 2 reactors in parallel

Electrode material: Gr(+) | Pt(−)

Current: 150 mA (each reactor)

Reaction flow rate: 0.3 mL/min (each reactor)

Residence time: 1 min

Total reaction time: 32 min

Conv.: >94%, NMR yield: 89%, Isolated yield: 0.43 g, 1.62 mmol, 85%

Productivity: 0.84 g/h

Spectral details:

^{1}H NMR (400 MHz, $CDCl_3$) δ 7.21 – 7.08 (m, 1H), 6.85 (t, *J* = 1.3 Hz, 2H), 6.18 (d, *J* = 1.1 Hz, 1H), 3.06 – 3.00 (m, 2H), 2.97 – 2.92 (m, 2H), 2.55 (s, 3H), 2.24 (s, 3H) ppm.

^{13}C NMR (101 MHz, $CDCl_3$) δ 195.2, 161.7 (dd, *J* = 246.7, 8.8 Hz), 157.3, 150.1, 127.8 (t, *J* = 10.3 Hz), 121.4, 116.9 (t, *J* = 20.1 Hz), 111.6 – 110.9 (m), 105.65, 40.6, 17.0 (t, *J* = 3.0 Hz), 14.5, 13.3 ppm

^{19}F NMR (376 MHz, $CDCl_3$) δ –115.6 (s, 1F) ppm.

1-(2,5-Dimethylfuran-3-yl)-3-phenylpropan-1-one (2l)

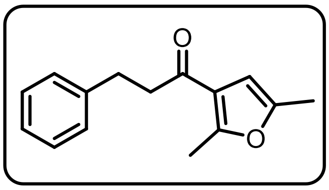

Compound **2l** was prepared according to the general procedure **GP4** from **S13** (2 g, 8.84 mmol, 0.1 M). The crude product was purified by flash column chromatography (EA/PE = 1:99), affording a yellow oil.

***e-flow* parameters:**

Configuration: 4 reactors in parallel

Electrode material: Gr(+) | Pt(−)

Current: 120 mA (each reactor)

Reaction flow rate: 0.3 mL/min (each reactor)

Residence time: 1 min

Total reaction time: 73 min (1 hr 13 min)

Isolated yield: 1.755 g, 7.69 mmol, 87%

Productivity: 1.44 g/h

Spectral details:

^{1}H NMR (400 MHz, CDCl$_3$) δ 7.33 – 7.27 (m, 2H), 7.25 – 7.17 (m, 3H), 6.18 (d, *J* = 1.2 Hz, 1H), 3.00 (s, 4H), 2.55 (s, 3H), 2.25 (s, 3H) ppm.

^{13}C NMR (101 MHz, CDCl$_3$) δ 195.7, 157.2, 150.1, 141.6, 128.6, 128.5, 126.2, 121.6, 105.7, 43.1, 30.0, 14.5, 13.3 ppm.

HRMS (ESI): m/z calculated for [$C_{15}H_{17}O_2$] [M+H$^+$]: 229.1231, measured: 229.1229.

3-(4-(Benzyloxy)phenyl)-1-(2,5-dimethylfuran-3-yl)propan-1-one (2m)

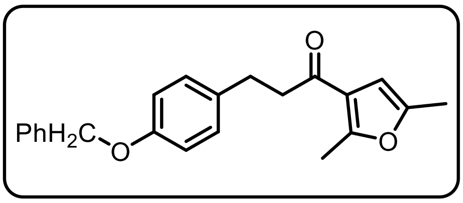

Compound **2m** was prepared according to the general procedure **GP4** from **S12** (0.5 g, 1.50 mmol, 0.1 M). The crude product was purified by flash column chromatography (EA/PE = 1:99), affording a colourless oil (0.381 g, 1.14 mmol, 76%).

***e-flow* parameters:**

Configuration: 2 reactors in parallel

Electrode material: Gr(+) | Pt(−)

Current: 150 mA (each reactor)

Reaction flow rate: 0.3 mL/min (each reactor)

Residence time: 1 min

Total reaction time: 25 min

conv.: >99%, NMR yield: 84%, Isolated yield: 0.40 g, 1.14 mmol, 76%

Productivity: 0.96 g/h

Spectral details:

^{1}H NMR (400 MHz, CDCl$_3$) δ 7.45 – 7.42 (m, 2H), 7.40 – 7.36 (m, 2H), 7.34 – 7.30 (m, 1H), 7.16 – 7.13 (m, 2H), 6.93 – 6.89 (m, 2H), 6.20 – 6.16 (m, 1H), 5.05 (d, *J* = 0.7 Hz, 2H), 2.98 – 2.92 (m, 4H), 2.55 (s, 3H), 2.25 (dd, *J* = 1.1, 0.5 Hz, 3H) ppm.

^{13}C NMR (101 MHz, CDCl$_3$) δ 195.9, 157.3, 157.2, 150.1, 137.3, 133.9, 129.5, 128.7, 128.1, 127.6, 121.7, 115.0, 105.7, 70.2, 43.3, 32.1, 14.3, 13.4 ppm.

1-(2,5-Dimethylfuran-3-yl)-3-(naphthalen-1-yl)propan-1-one (2n)

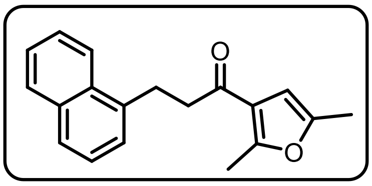

Compound **2n** was prepared according to the general procedure **GP4** from **S10** (0.5 g, 1.8 mmol, 0.1 M). The crude product was purified by flash column chromatography (EA/PE = 1:99), affording a colourless liquid.

***e-flow* parameters:**

Configuration: 2 reactors in parallel

Electrode material: Gr(+) | Pt(−)

Current: 150 mA (each reactor)

Reaction flow rate: 0.3 mL/min (each reactor)

Residence time: 1 min

Total reaction time: 30 min

Isolated yield: 0.42 g, 1.5 mmol, 83%

Productivity: 0.84 g/h

Spectral details:

^{1}H NMR (400 MHz, CDCl$_3$) δ 8.07 – 8.02 (m, 1H), 7.89 – 7.85 (m, 1H), 7.77 – 7.71 (m, 1H), 7.55 – 7.46 (m, 2H), 7.43 – 7.35 (m, 2H), 6.14 (d, *J* = 1.2 Hz, 1H), 3.46 (t, 2H), 3.12 (t, 2H), 2.57 (s, 3H), 2.22 (s, 3H) ppm.

^{13}C NMR (101 MHz, CDCl$_3$) δ 195.9, 157.3, 150.1, 137.6, 134.0, 131.8, 129.0, 127.0, 126.2, 125.8, 125.7, 123.7, 121.6, 105.7, 42.3, 27.0, 14.5, 13.3 ppm.

(E)-3-(4-chlorophenyl)-1-phenylprop-2-en-1-one (2o)

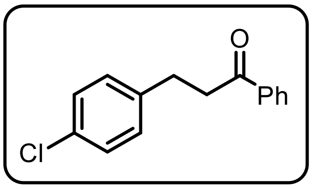

Compound **2o** was prepared according to the general procedure **GP4** from **S14** (1 g, 4.12 mmol, 0.1 M). The crude product was purified by flash column chromatography (EA/PE = 1:99), affording a colourless liquid.

***e-flow* parameters:**

Configuration: 4 reactors in parallel

Electrode material: Gr(+) | Pt(−)

Current: 150 mA (each reactor)

Reaction flow rate: 0.3 mL/min (each reactor)

Residence time: 1 min

Total reaction time: 35 min

Isolated yield: 0.76 g, 1.5 mmol, 76%

Productivity: 1.30 g/h

Spectral details:

^{1}H NMR (500 MHz, CDCl$_3$) δ 7.80 – 7.73 (m, 2H), 7.40 – 7.36 (m, 1H), 7.31 – 7.26 (m, 2H), 7.08 (d, *J* = 8.4 Hz, 2H), 7.01 (d, *J* = 8.4 Hz, 2H), 3.10 (t, *J* = 7.0 Hz, 2H), 2.87 (t, *J* = 7.5 Hz, 2H) ppm.

^{13}C NMR (126 MHz, CDCl$_3$) δ 199.0, 139.9, 136.8, 133.3, 132.0, 130.0, 128.8, 128.7, 128.1, 40.3, 29.5 ppm.

Ethyl 3-phenylpropanoate (2r)

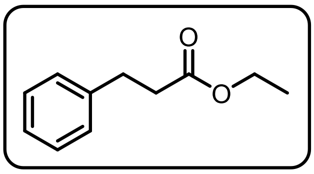

Compound **2r** was prepared according to the general procedure **GP4** from ethyl cinnamate (1 g, 5.68 mmol, 0.1 M). The crude product was purified by flash column chromatography (EA/PE = 0.5:99.5), affording a colourless liquid.

***e-flow* parameters:**

Configuration: 4 reactors in parallel; 2 reactors in series

Electrode material: Gr(+) | Gr(−)

Current: 120 mA (each reactor, both in series and parallel)

Reaction flow rate: 0.3 mL/min (each reactor)

Residence time: 1 min

Total reaction time: 48 min (parallel), 190 min (series)

Conv.: >99% (in series); 79% (in parallel), NMR yield: 98% (in series), 75% (in parallel) ; Isolated yield: series 2-reactor; 0.992 g, 5.50 mmol, 97%; parallel 2-reactor: 0.75 g, 4.20 mmol, 74%

Productivity: 0.94 g/h (parallel), 0.314 g/h (series)

Spectral details:

^{1}H NMR (400 MHz, CDCl$_3$) δ 7.30 (dd, *J* = 7.8, 6.9 Hz, 2H), 7.25 – 7.18 (m, 3H), 4.14 (q, *J* = 7.1 Hz, 2H), 2.96 (t, *J* = 7.9 Hz, 2H), 2.63 (t, *J* = 7.9 Hz, 2H), 1.24 (t, *J* = 7.1 Hz, 3H) ppm.

^{13}C NMR (101 MHz, CDCl$_3$) δ 173.0, 140.7, 128.6, 128.4, 126.3, 60.5, 36.1, 31.1, 14.3 ppm.

HRMS (ESI): m/z calculated for [$C_{11}H_{14}O_2$] [M^+]: 178.0988, measured: 178.0986.

The analytical data are in accordance with reported literature.[8]

Methyl 3-phenylpropanoate (2s)

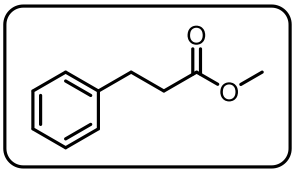

Compound **2s** was prepared according to the general procedure **GP4** from methyl cinnamate (2 g, 12.33 mmol, 0.2 M). The crude product was purified by flash column chromatography (EA/PE = 0.5:99.5), affording a colourless liquid.

***e-flow* parameters:**

Configuration: 4 reactors in parallel

Electrode material: Gr(+) | Gr(−)

Current: 260 mA (each reactor)

Reaction flow rate: 0.3 mL/min (each reactor)

Residence time: 1 min

Total reaction time: 52 min

Conv.: >99%, NMR yield: 96%, Isolated yield: 1.456 g, 8.88 mmol, 72%

Note: Some product lost during evaporation under vacuum because of its low boiling point.

Productivity: 1.68 g/h

Spectral details:

^{1}H NMR (400 MHz, $CDCl_3$) δ 7.30 – 7.23 (m, 2H), 7.21 – 7.14 (m, 3H), 3.64 (s, 3H), 2.93 (t, *J* = 7.9 Hz, 2H), 2.61 (t, *J* = 7.9 Hz, 2H) ppm.

^{13}C NMR (101 MHz, $CDCl_3$) δ 173.3, 140.6, 128.6, 128.3, 126.3, 51.6, 35.7, 31.0 ppm.

HRMS (ESI): m/z calculated for [$C_{10}H_{12}O_2$] [M^+]: 164.0831, measured: 164.0826.

The analytical data are in accordance with reported literature.[8]

4-Phenylbutan-2-one (2t)

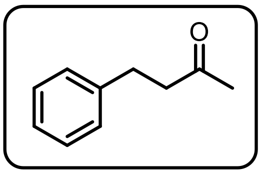

Compound **2t** was prepared according to the general procedure **GP4** from (E)-4-phenylbut-3-en-2-one (5 g, 34.24 mmol, 0.1 M). The crude product was purified by flash column chromatography (EA/PE = 0.5:99.5), affording a white solid.

***e-flow* parameters:**

Configuration: 4 reactors in parallel

Electrode material: Gr(+) | Gr(−)

Current: 120 mA (each reactor)

Reaction flow rate: 0.3 mL/min (each reactor)

Residence time: 1 min

Total reaction time: 4 hr 46 min

Conv.: 87%, NMR yield: 55%, Isolated yield: 3 g, 20.20 mmol, 59%

Productivity: 0.66 g/h

Spectral details:

^{1}H NMR (400 MHz, CDCl$_3$) δ 7.20 – 7.15 (m, 2H), 7.10 – 7.07 (m, 3H), 2.79 (t, J = 7.6 Hz, 2H), 2.64 (t, J = 7.7 Hz, 2H), 2.02 (s, 3H) ppm.

^{13}C NMR (101 MHz, CDCl$_3$) δ 207.9, 141.0, 128.5, 128.3, 126.1, 45.1, 45.1, 30.0, 29.7 ppm.

Trace amount of inseparable impurities present.

The analytical data are in accordance with reported literature.[9]

3-Phenylpropanoic acid (2u)

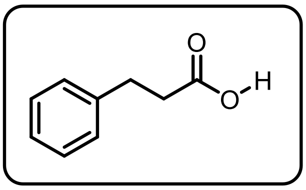

Compound **2u** was prepared according to the general procedure **GP4** from cinnamic acid (2 g, 13.50 mmol, 0.2 M) with slight modification in the workup procedure: acidic work-up perform during extraction. The crude product was purified by flash column chromatography (EA/PE = 10:90), affording a white solid.

***e-flow* parameters:**

Configuration: 4 reactors in parallel

Electrode material: Gr(+) | Gr(−)

Current: 300 mA (each reactor)

Reaction flow rate: 0.3 mL/min (each reactor)

Residence time: 1 min

Total reaction time: 57 min

Conv.: not resolved, NMR yield: 54%, Isolated yield: 1.053 g, 7.02 mmol, 52%

Productivity: 1.11 g/h

Spectral details:

^{1}H NMR (400 MHz, CDCl$_3$) δ 7.34 – 7.27 (m, 2H), 7.25 – 7.16 (m, 3H), 2.97 (t, J = 7.8 Hz, 2H), 2.70 (t, J = 8.1 Hz, 2H) ppm.

^{13}C NMR (101 MHz, CDCl$_3$) δ 179.1, 140.3, 128.7, 128.4, 126.5, 35.7, 30.7 ppm.

The analytical data are in accordance with reported literature.[10]

Reference

(1) Huang, B.; Li, Y.; Yang, C.; Xia, W. Electrochemical 1,4-Reduction of α,β-Unsaturated Ketones with Methanol and Ammonium Chloride as Hydrogen Sources. *Chem. Commun.* **2019**, *55* (47), 6731–6734.

(2) Iftikhar, S.; Khan, S.; Bilal, A.; Manzoor, S.; Abdullah, M.; Emwas, A.-H.; Sioud, S.; Gao, X.; Chotana, G. A.; Faisal, A.; Saleem, R. S. Z. Synthesis and Evaluation of Modified Chalcone Based P53 Stabilizing Agents. *Bioorg. Med. Chem. Lett.* **2017**, *27* (17), 4101–4106.

(3) Nicholson, K.; Langer, T.; Thomas, S. P. Borane-Catalyzed, Chemoselective Reduction and Hydrofunctionalization of Enones Enabled by B–O Transborylation. *Org. Lett.* **2021**, *23* (7), 2498–2504.

(4) Atreya, V.; Jalwal, S.; Chakraborty, S. Chromium-Catalyzed Sustainable C–C and C–N Bond Formation: C -Alkylation and Friedländer Quinoline Synthesis Using Alcohols. *Dalton Trans.* **2025**, *54* (3), 1212–1221.

(5) Wu, P.; Jiao, G.-S.; Zhang, C.-P. Nickel-Catalyzed Synthesis of Aryl Ketones from Arylsulfonium Salts and Nitriles. *Org. Chem. Front.* **2025**, *12* (24), 7097–7106.

(6) Singh, A.; Jaiswal, V.; Misra, S.; Singh, A. K. Metal–Ligand Cooperativity via Π–Π Interactions Supporting Outer-Sphere Hydride Transfer. *Chem. – Eur. J.* **2025**, *31* (56), e202501301.

(7) Kishore, D. R.; Goel, K.; Shekhar, C.; Satyanarayana, G. An Access to Benzo[a]Fluorenes, Benzo[b]Fluorenes, and Indenes Triggered by Simple Lewis Acid. *J. Org. Chem.* **2022**, *87* (5), 2178–2203.

(8) Deng, Z.; Han, S.; Ke, M.; Ning, Y.; Chen, F.-E. Ligand-Enabled Palladium-Catalyzed Hydroesterification of Vinyl Arenes with High Linear Selectivity to Access 3-Arylpropanoate Esters. *Chem. Commun.* **2022**, *58* (24), 3921–3924.

(9) Gu, Y.; Norton, J. R.; Salahi, F.; Lisnyak, V. G.; Zhou, Z.; Snyder, S. A. Highly Selective Hydrogenation of C═C Bonds Catalyzed by a Rhodium Hydride. *J. Am. Chem. Soc.* **2021**, *143* (25), 9657–9663.

(10) Speckmeier, E.; Zeitler, K. Desyl and Phenacyl as Versatile, Photocatalytically Cleavable Protecting Groups: A Classic Approach in a Different (Visible) Light. *ACS Catal.* **2017**, *7* (10), 6821–6826.

10. NMR spectra

10.1. NMR spectra for α,β-unsaturated ketone derivatives

(E)-1-(4-(benzyloxy)phenyl)-3-phenylprop-2-en-1-one (S1)

^{1}H NMR (400 MHz, CDCl$_3$)

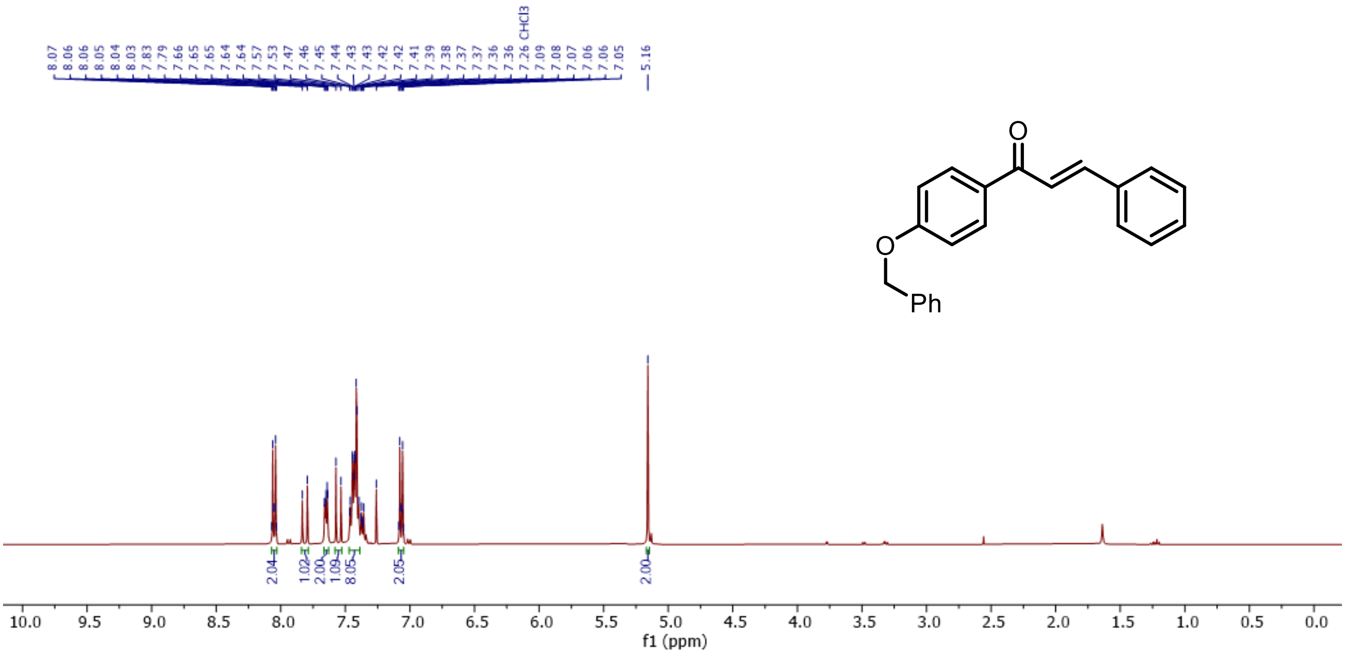

^{13}C NMR (101 MHz, CDCl$_3$)

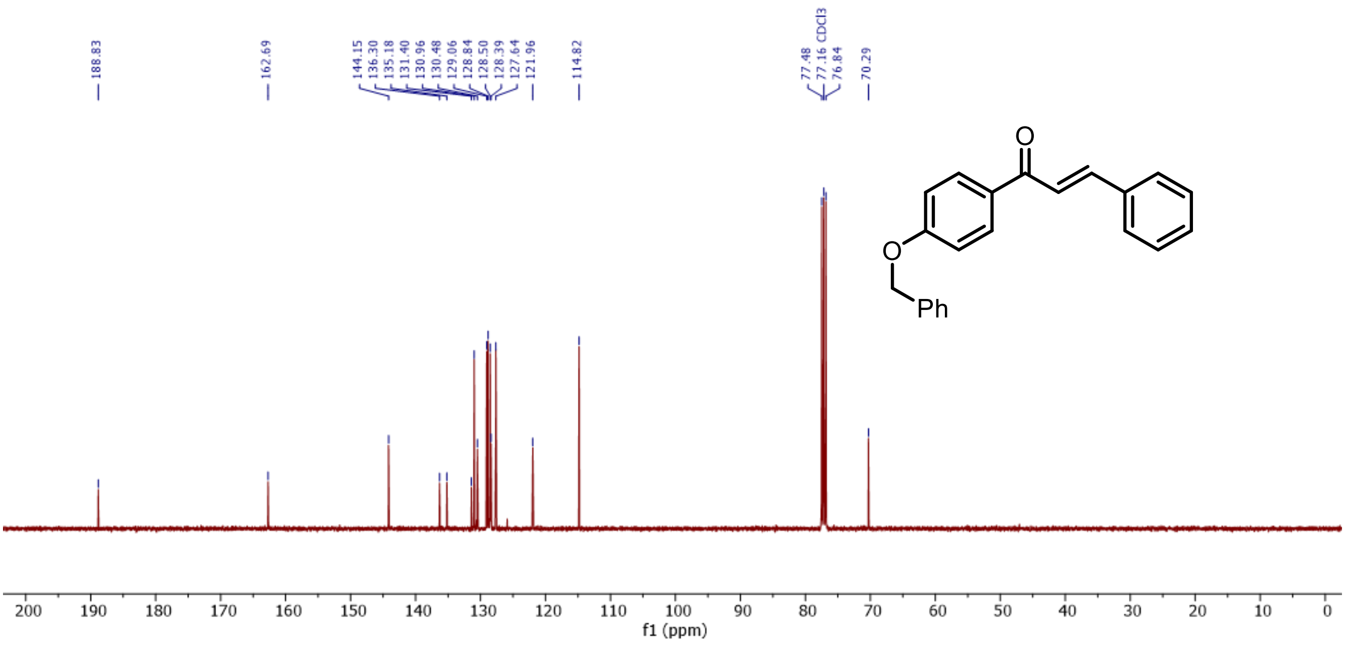

(E)-3-phenyl-1-(p-tolyl)prop-2-en-1-one (S2)

^{1}H NMR (400 MHz, $CDCl_3$)

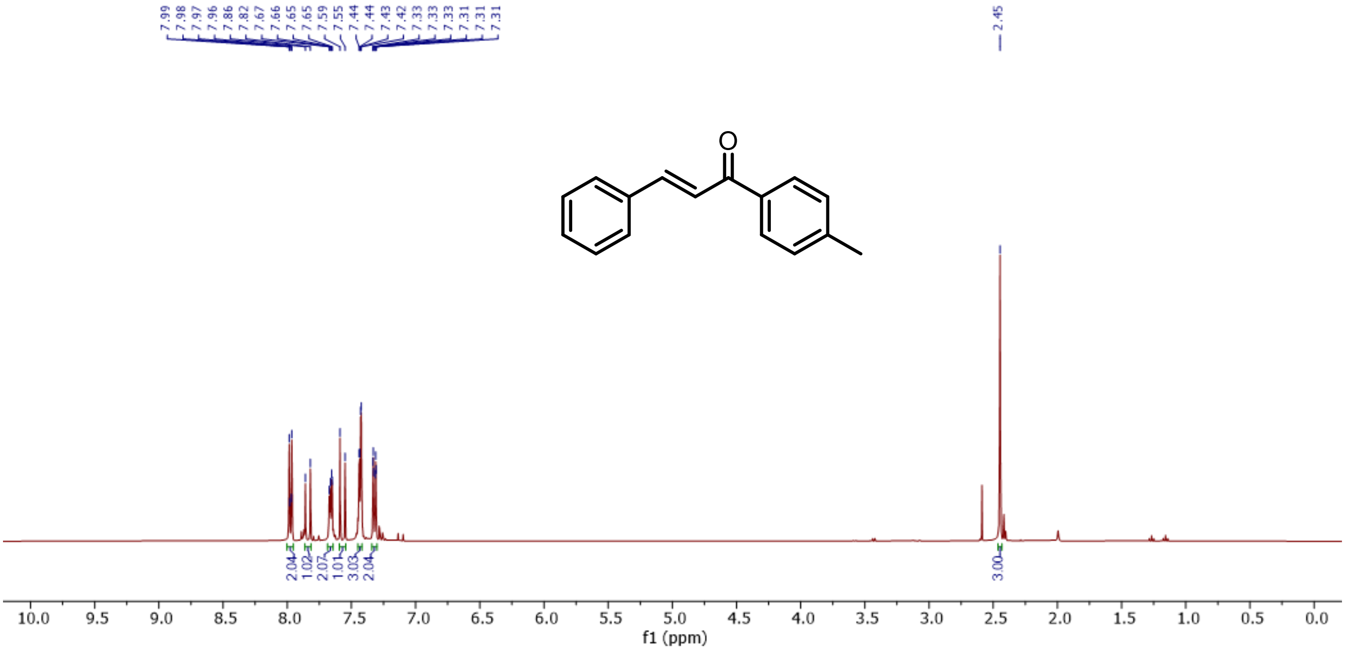

^{13}C NMR (101 MHz, $CDCl_3$)

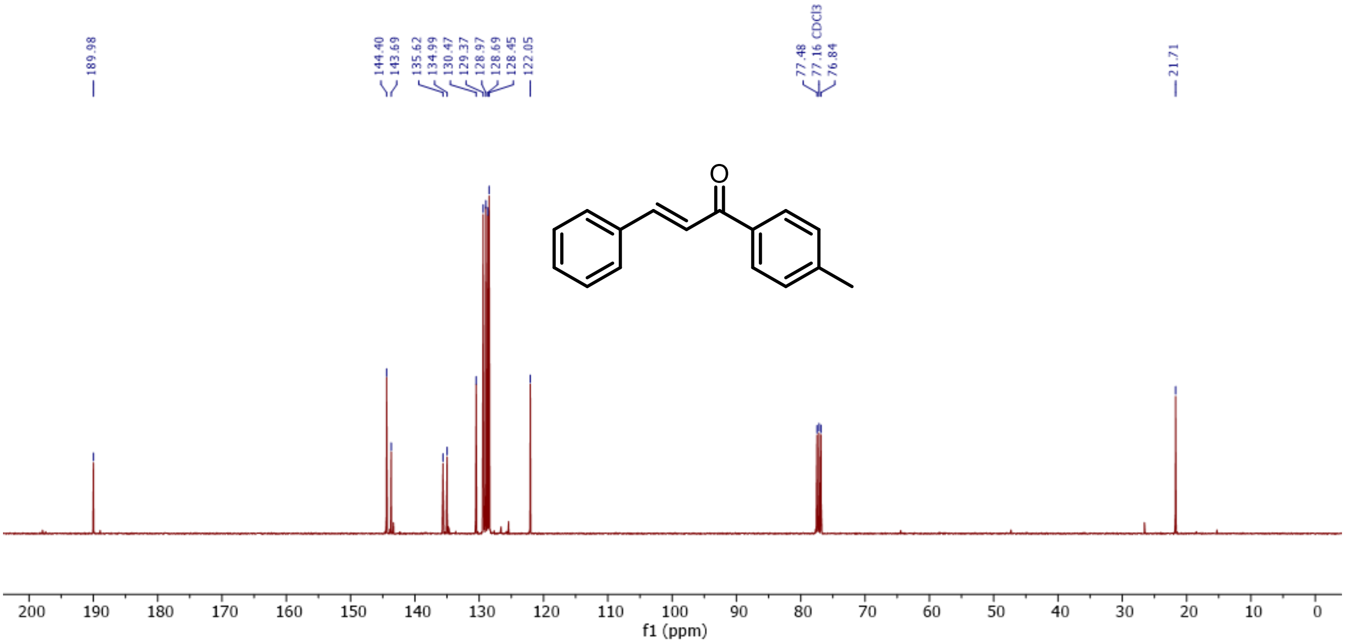

(E)-1-([1,1'-biphenyl]-4-yl)-3-phenylprop-2-en-1-one (S3)

^{1}H NMR (400 MHz, CDCl$_3$)

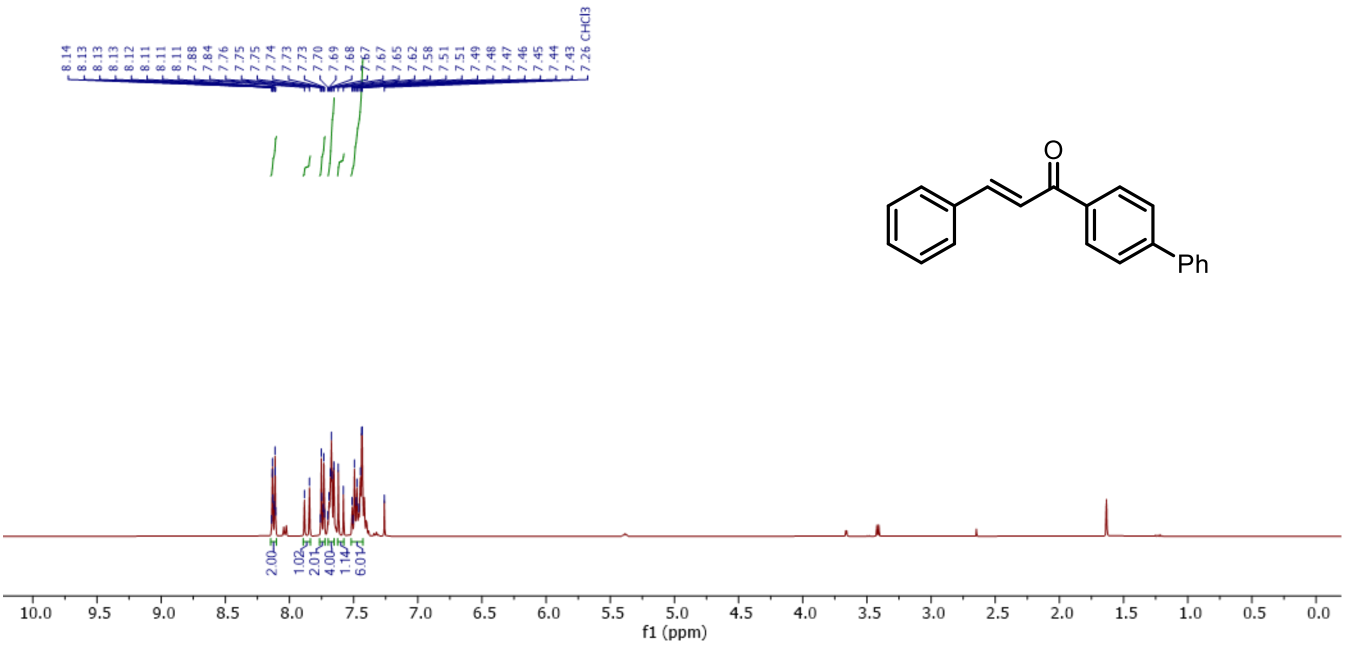

^{13}C NMR (101 MHz, CDCl$_3$)

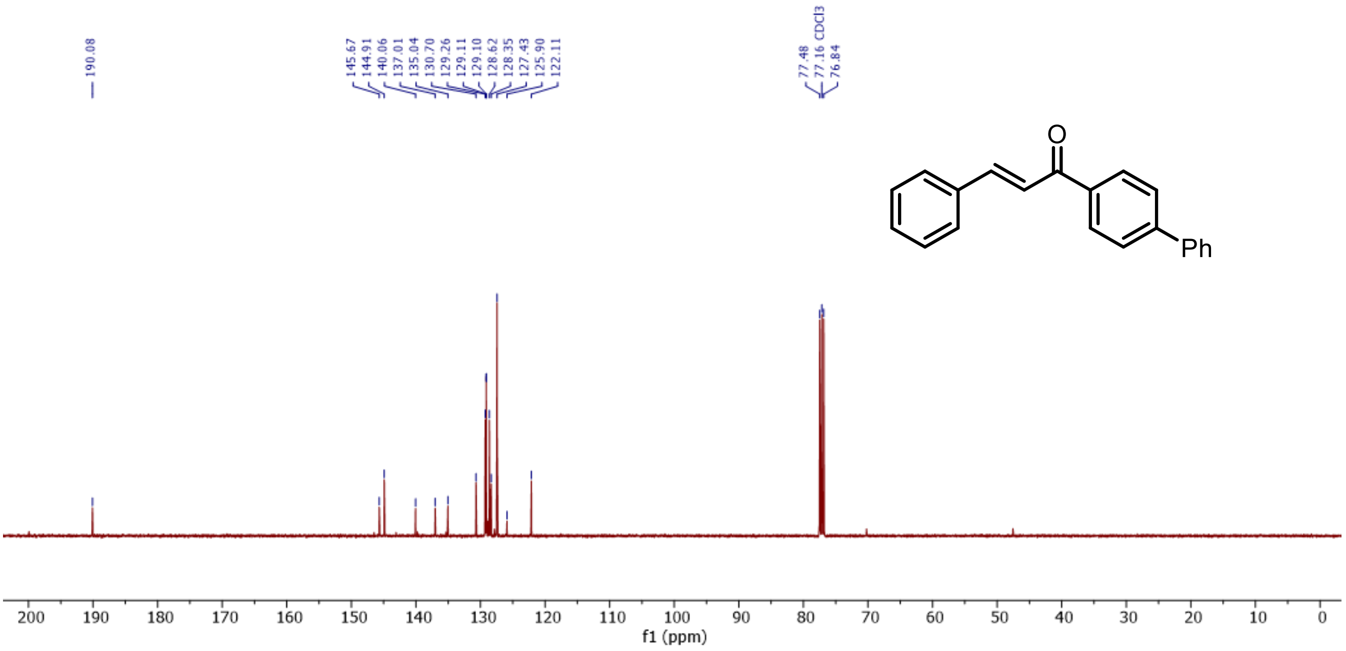

(E)-1-(2-fluorophenyl)-3-(naphthalen-2-yl)prop-2-en-1-one (S4)

^{1}H NMR (400 MHz, CDCl$_3$)

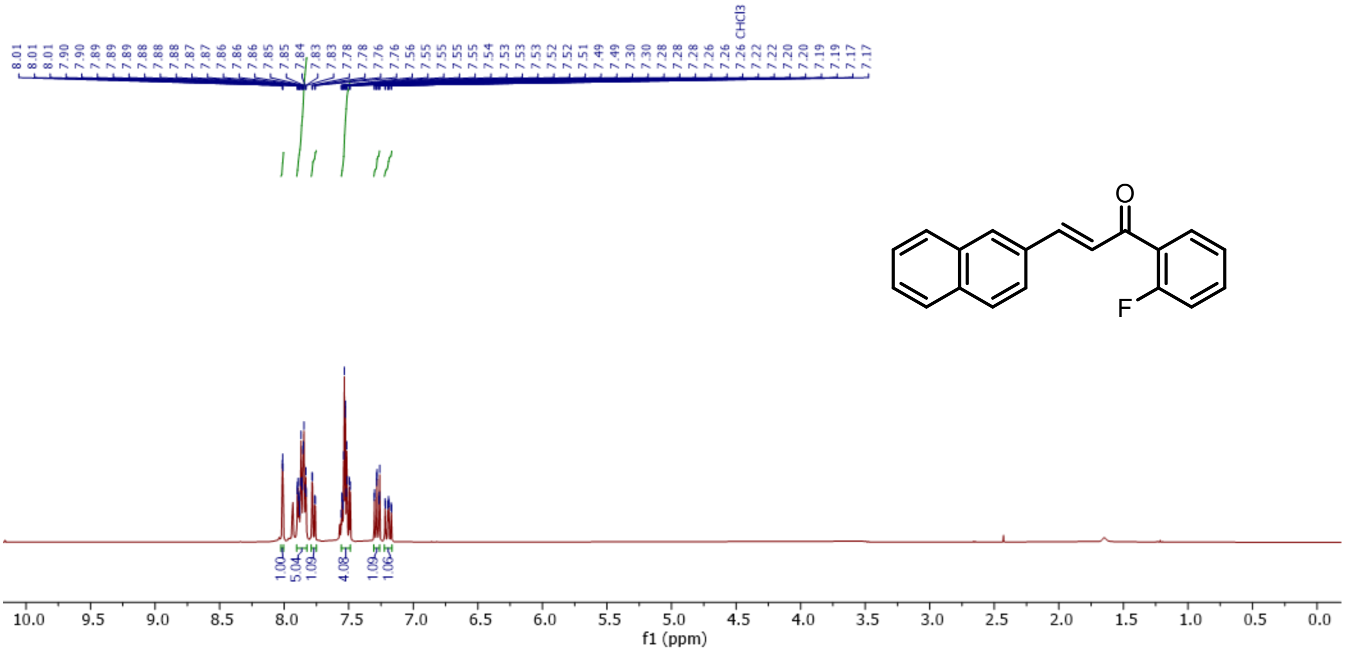

^{13}C NMR (101 MHz, CDCl$_3$)

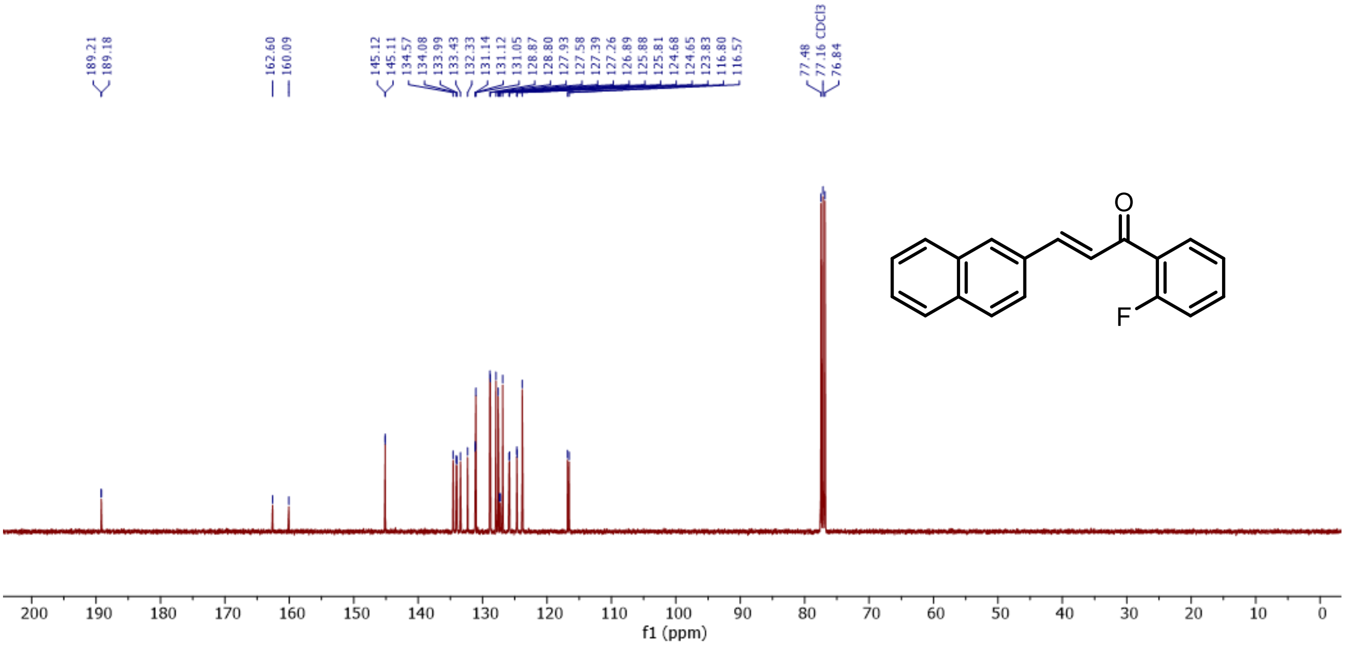

^{19}F NMR (376 MHz, CDCl$_3$)

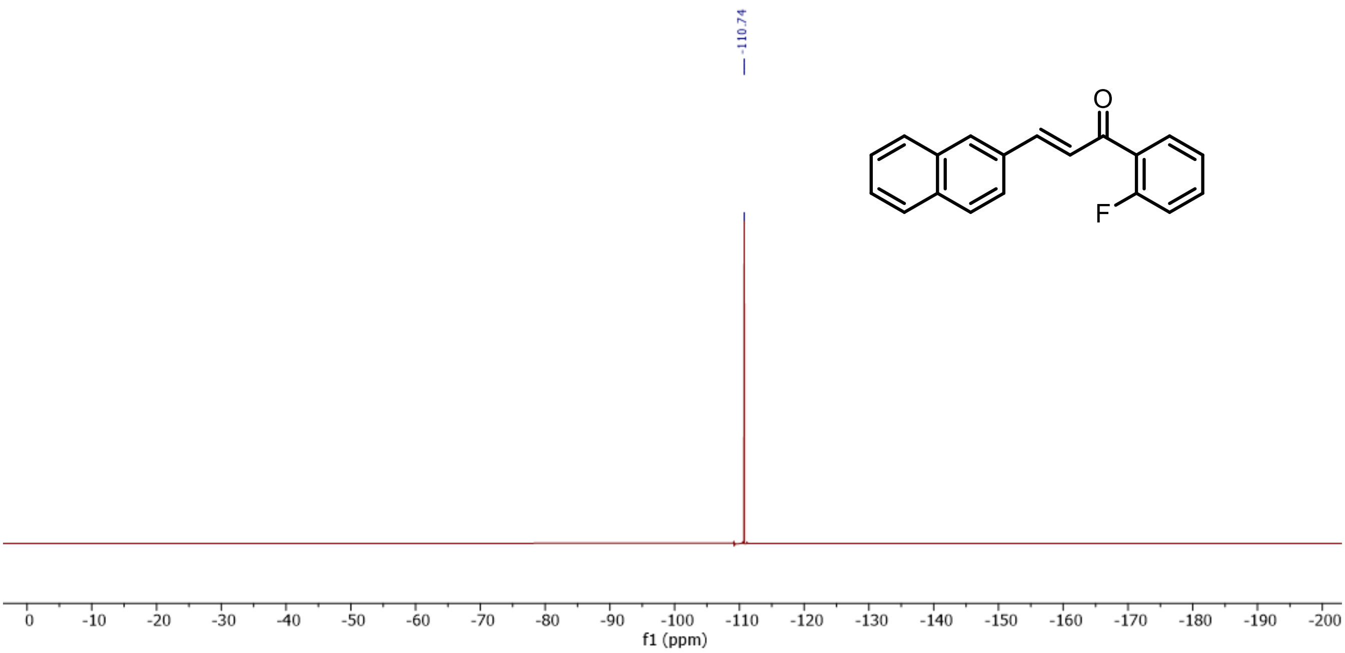

(E)-3-(2-bromo-4,5-dimethoxyphenyl)-1-(p-tolyl)prop-2-en-1-one (S5)

^{1}H NMR (400 MHz, CDCl$_3$)

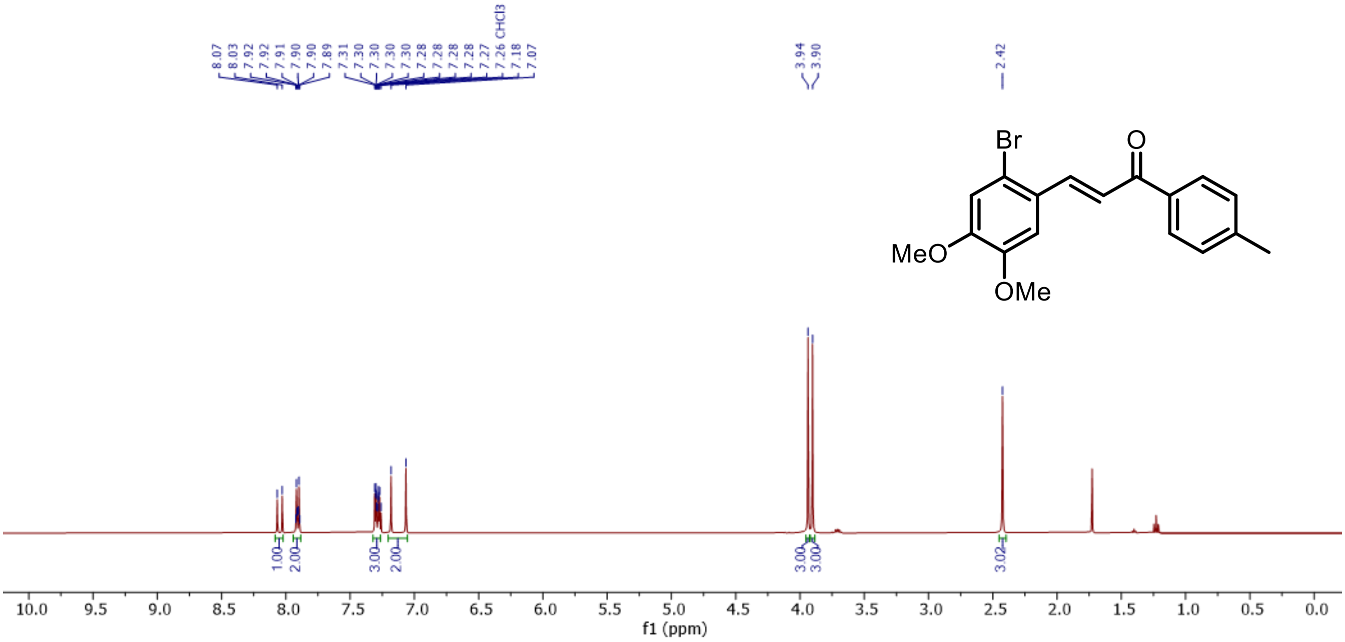

^{13}C NMR (101 MHz, CDCl$_3$)

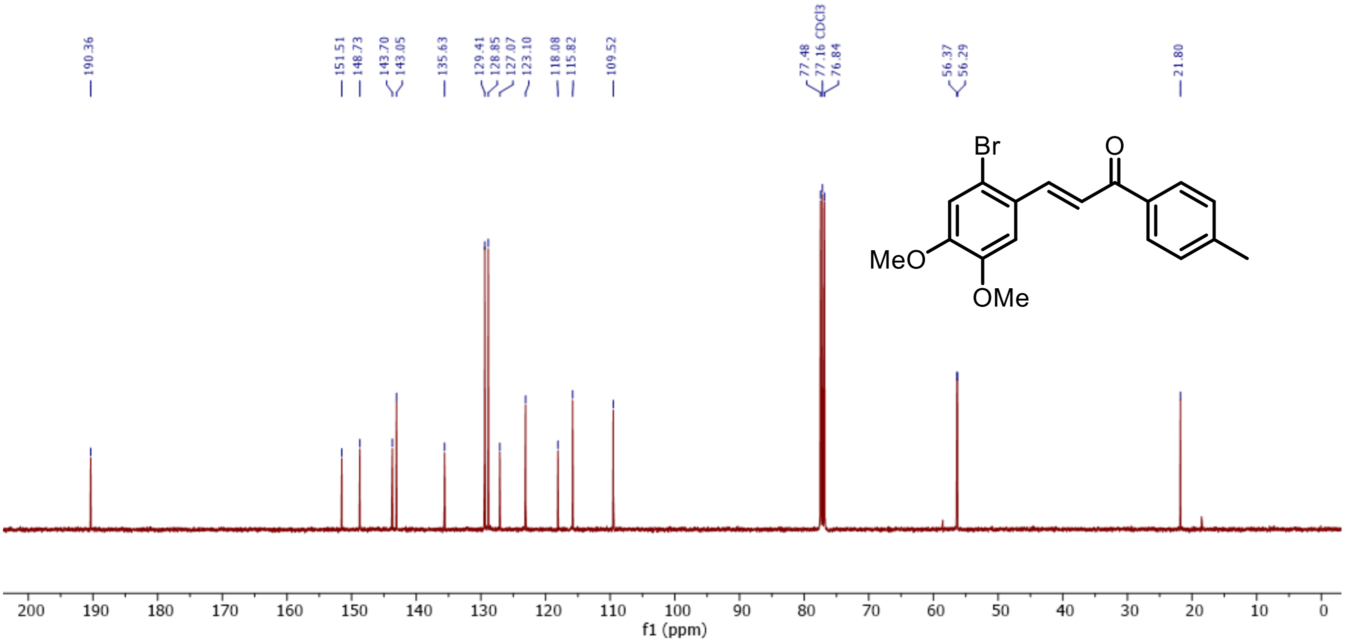

(E)-3-(benzo[d][1,3]dioxol-5-yl)-1-(p-tolyl)prop-2-en-1-one (S6)

^{1}H NMR (400 MHz, CDCl$_3$)

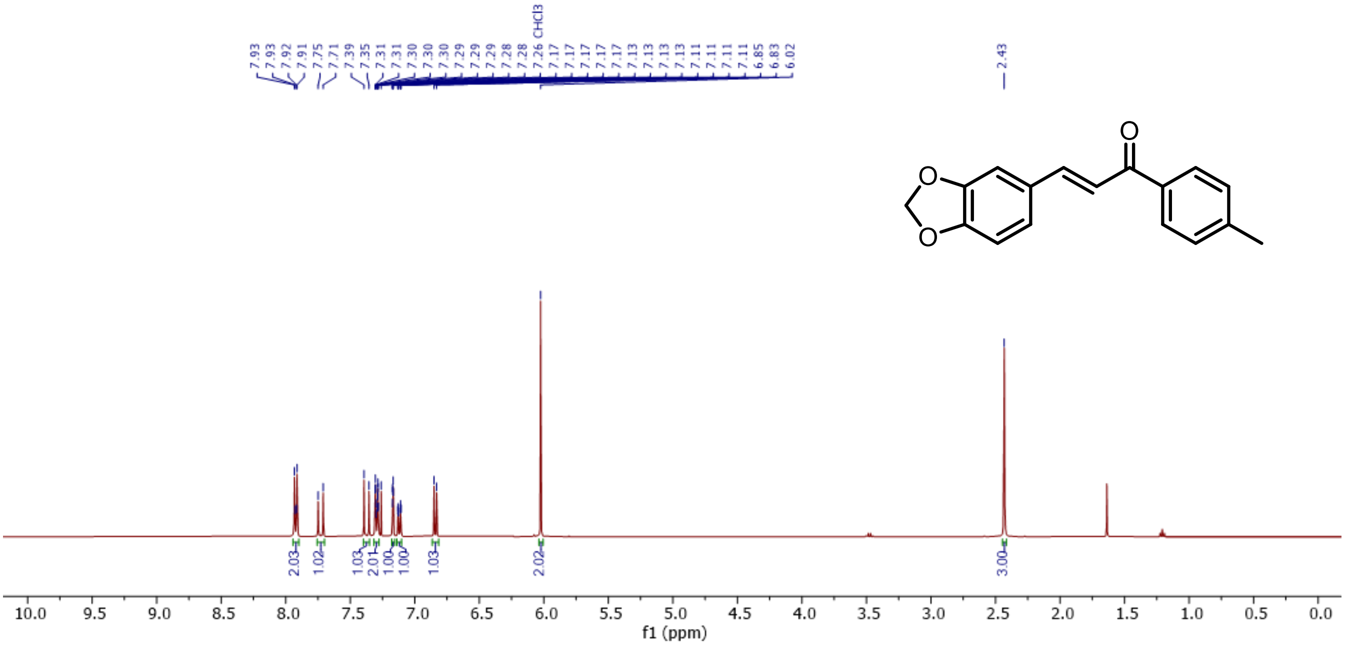

^{13}C NMR (101 MHz, CDCl$_3$)

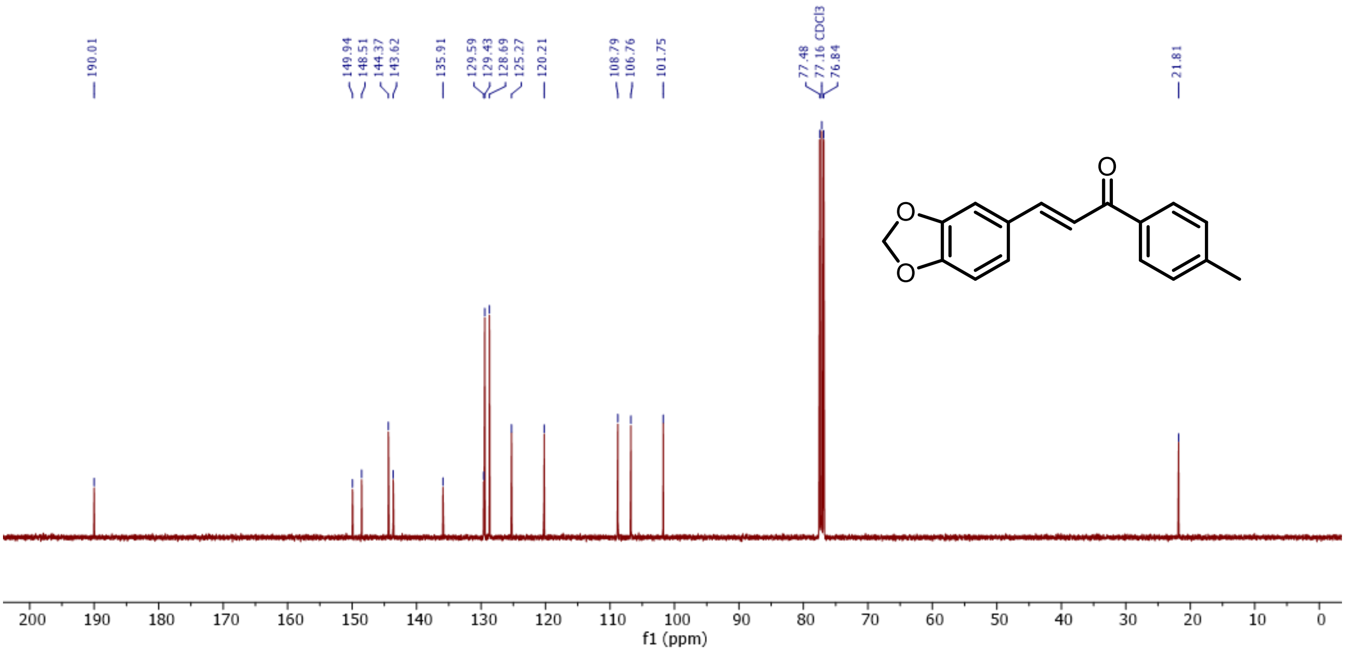

(E)-3-(2-ethynylphenyl)-1-(p-tolyl)prop-2-en-1-one (S7)

^{1}H NMR (400 MHz, CDCl$_3$)

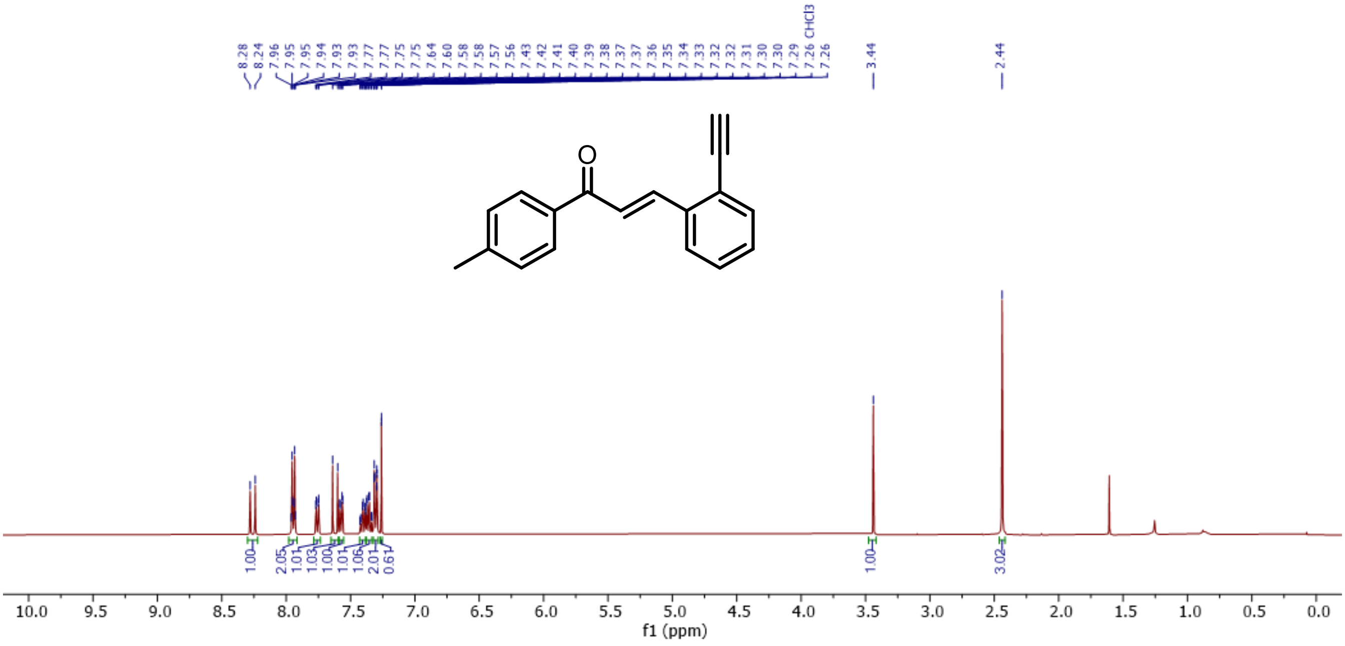

^{13}C NMR (101 MHz, $CDCl_3$)

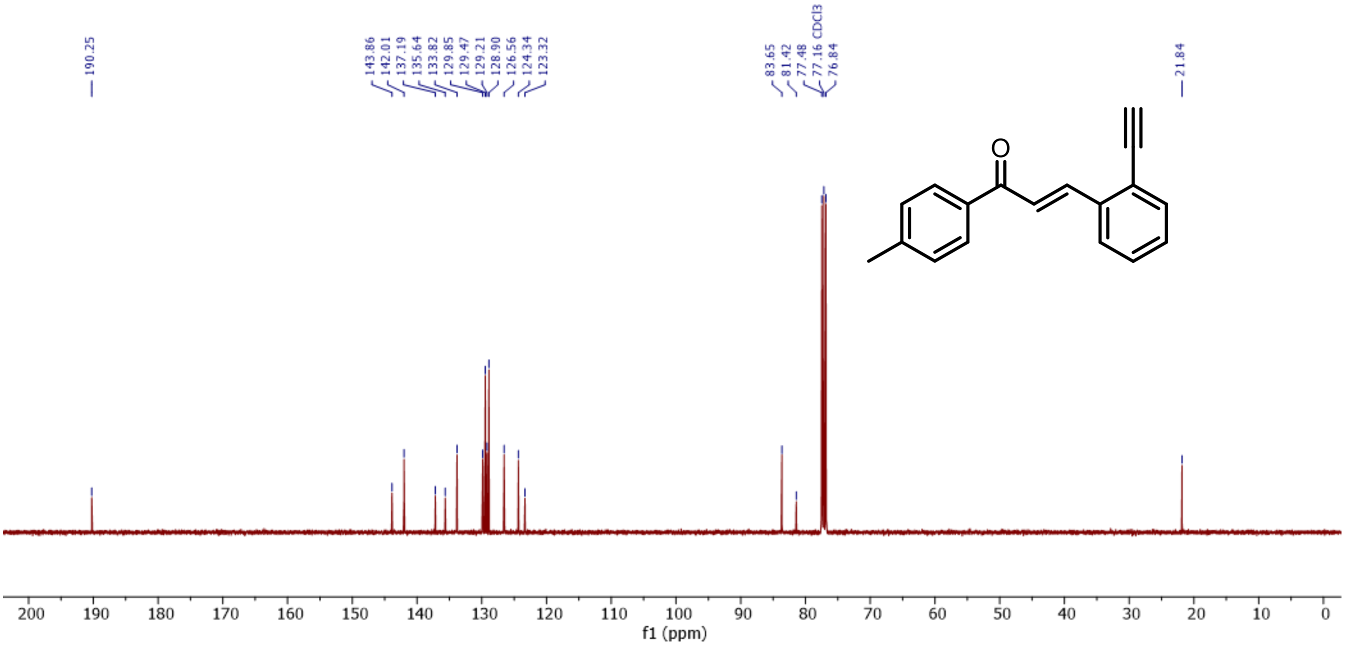

(E)-3-phenyl-1-(thiophen-2-yl)prop-2-en-1-one (S8)

^{1}H NMR (400 MHz, $CDCl_3$)

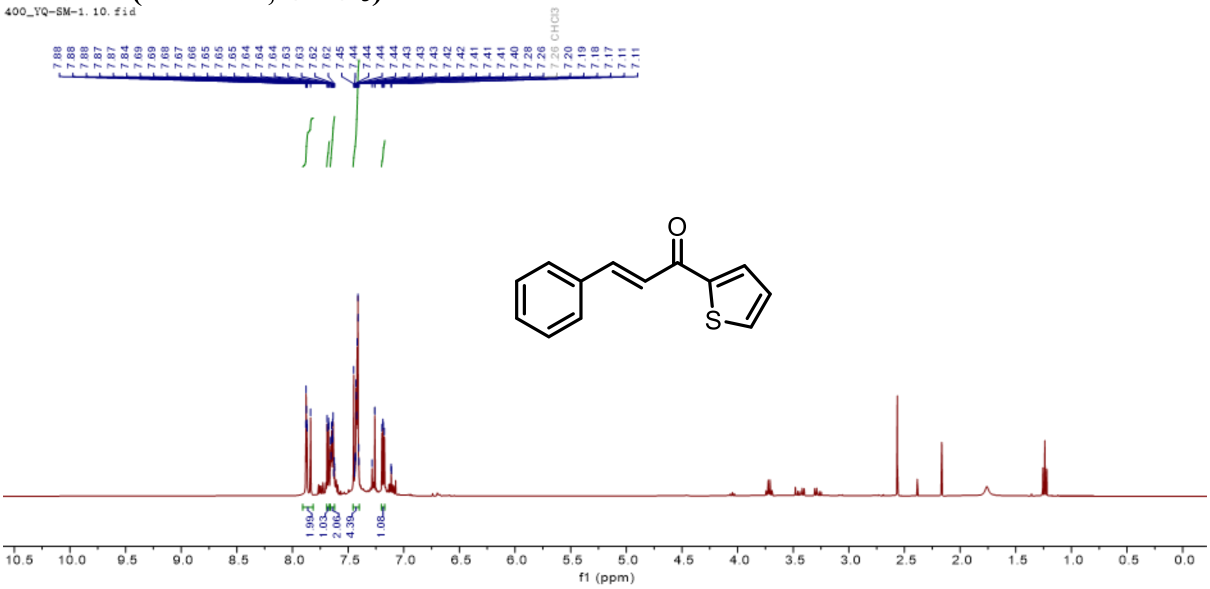

^{13}C NMR (101 MHz, $CDCl_3$)

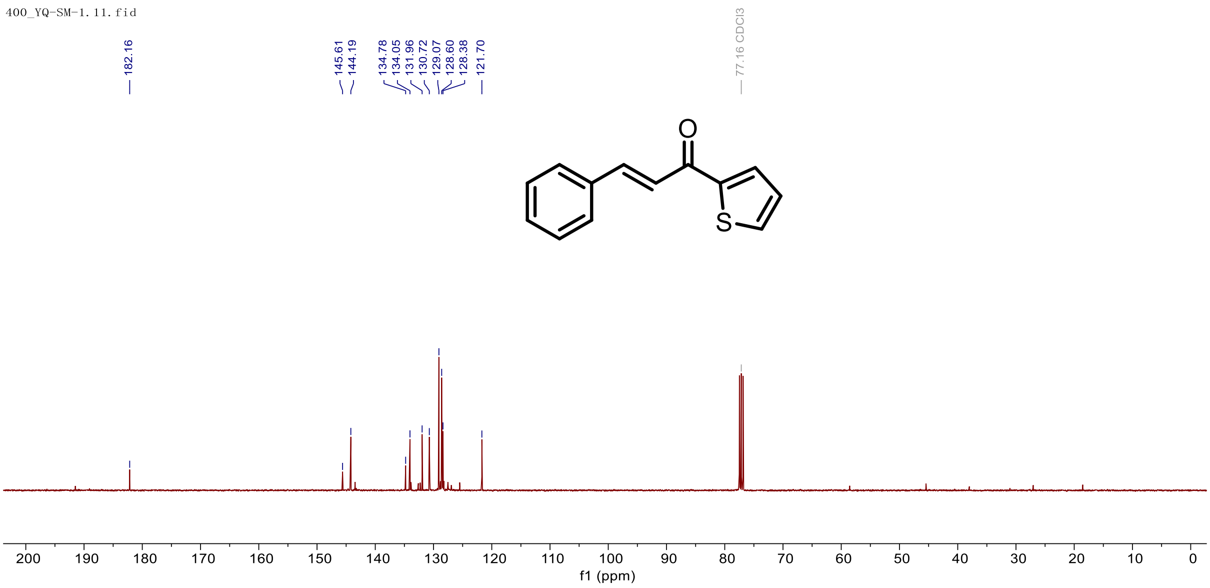

(E)-3-phenyl-1-(thiophen-3-yl)prop-2-en-1-one (S9)

^{1}H NMR (400 MHz, $CDCl_3$)

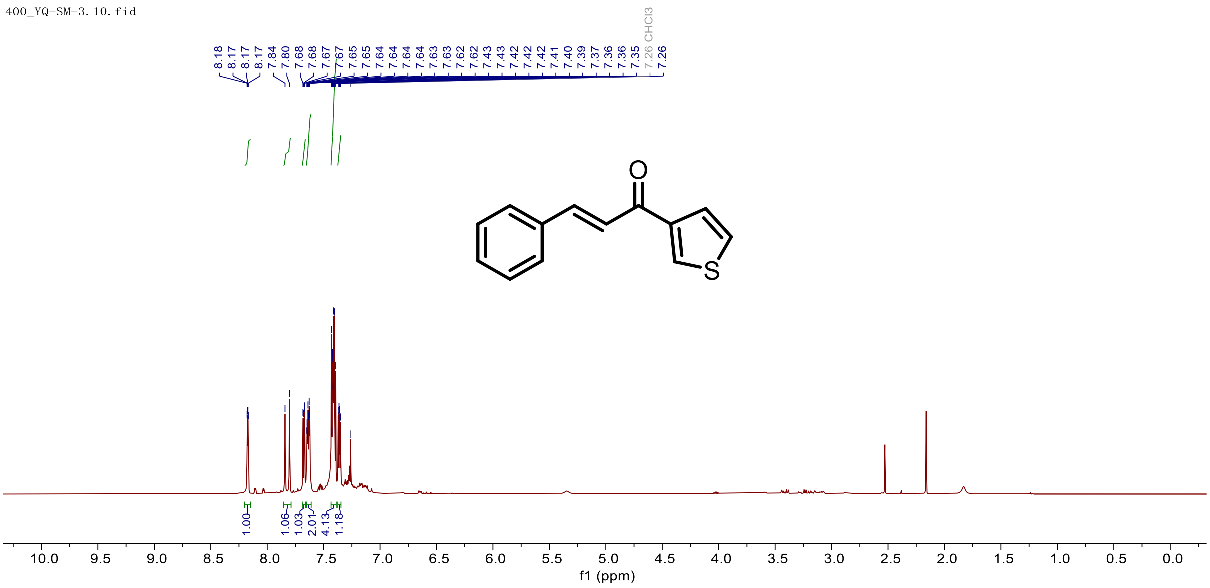

^{13}C NMR (101 MHz, $CDCl_3$)

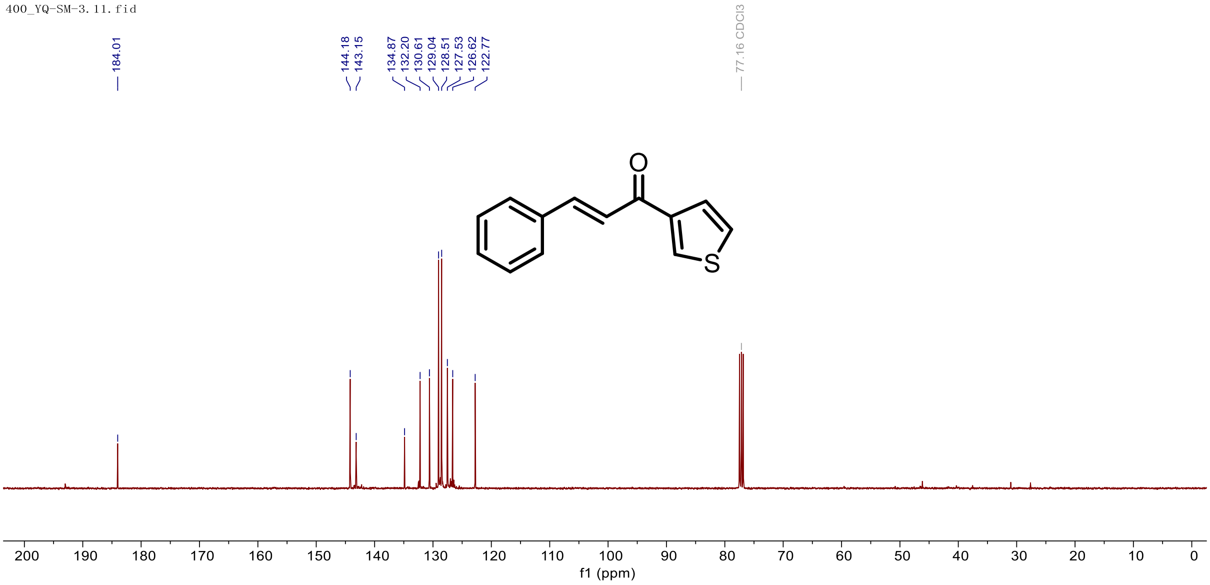

(E)-3-phenyl-1-(thiophen-2-yl)prop-2-en-1-one (S10)

^1H NMR (400 MHz, $CDCl_3$)

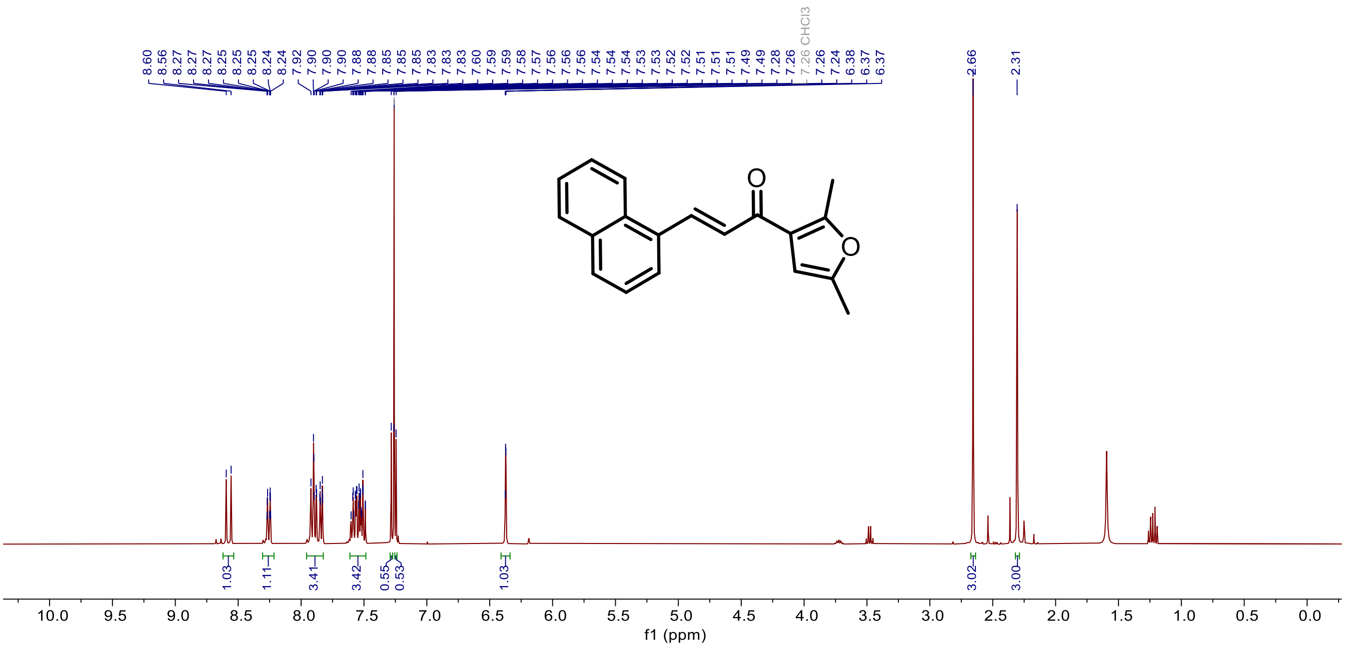

^{13}C NMR (101 MHz, CDCl$_3$)

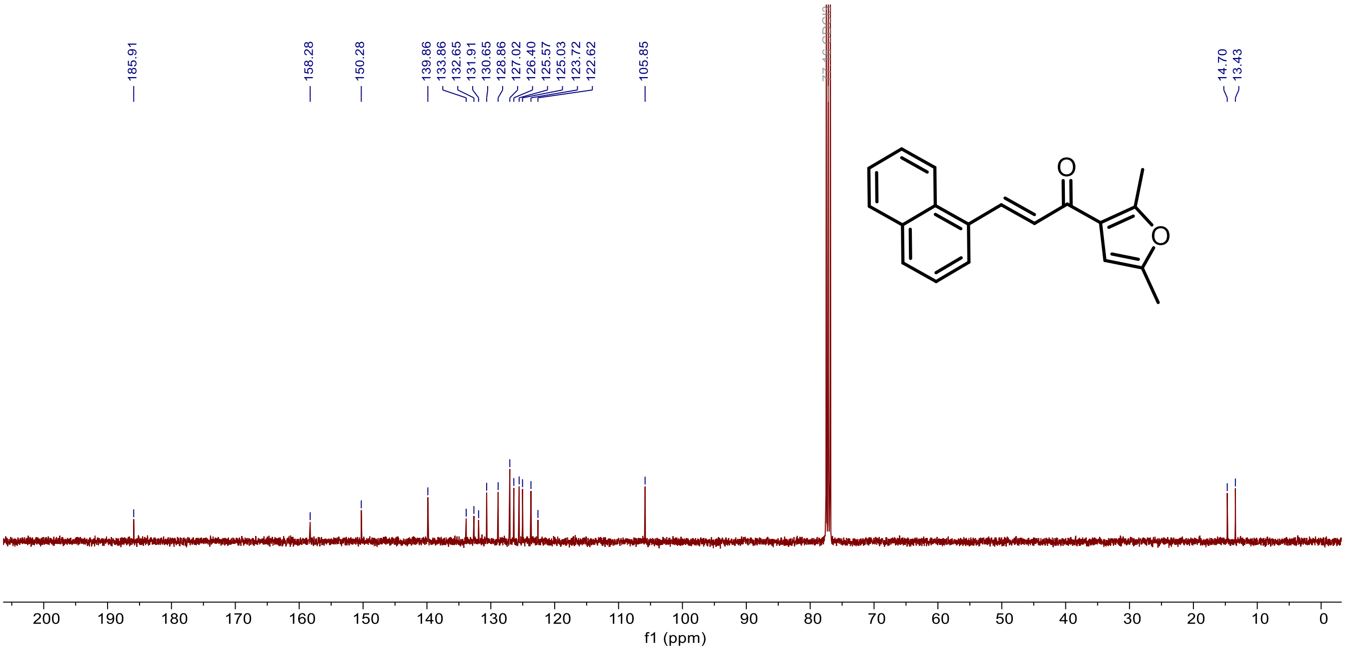

(E)-3-(2,6-difluorophenyl)-1-(2,5-dimethylfuran-3-yl)prop-2-en-1-one (S11)

^{1}H NMR (400 MHz, CDCl$_3$)

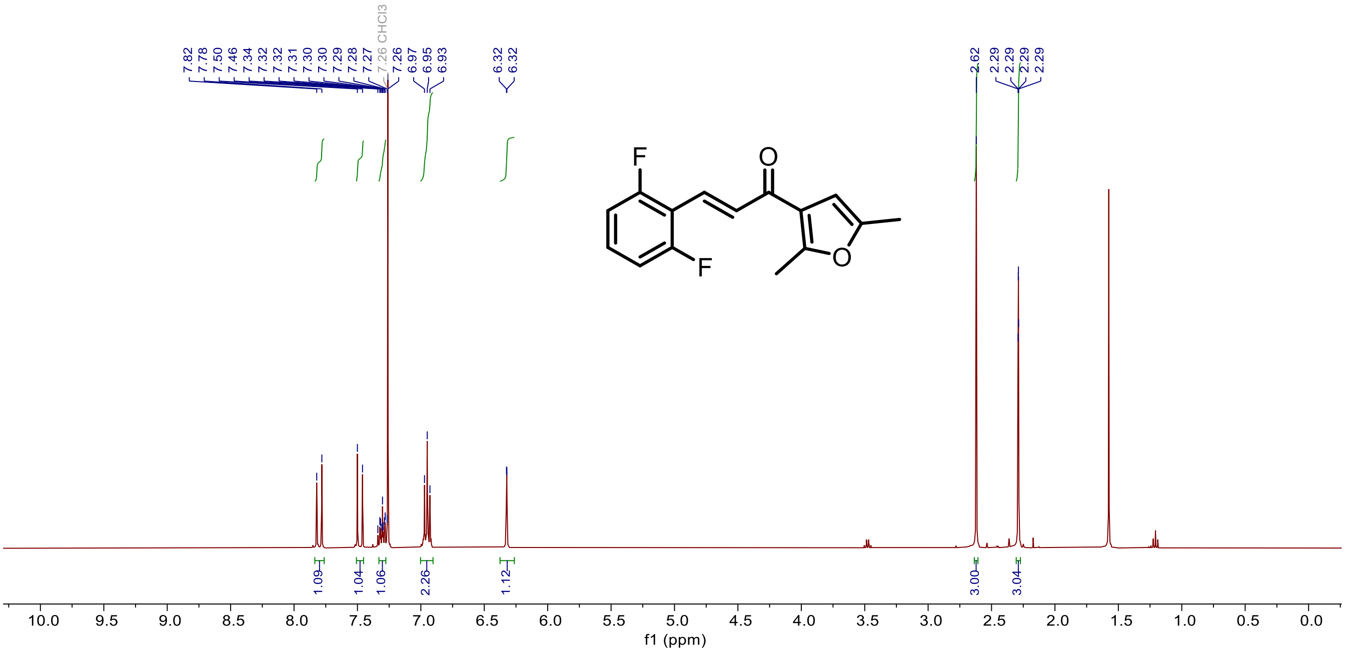

^{13}C NMR (101 MHz, CDCl$_3$)

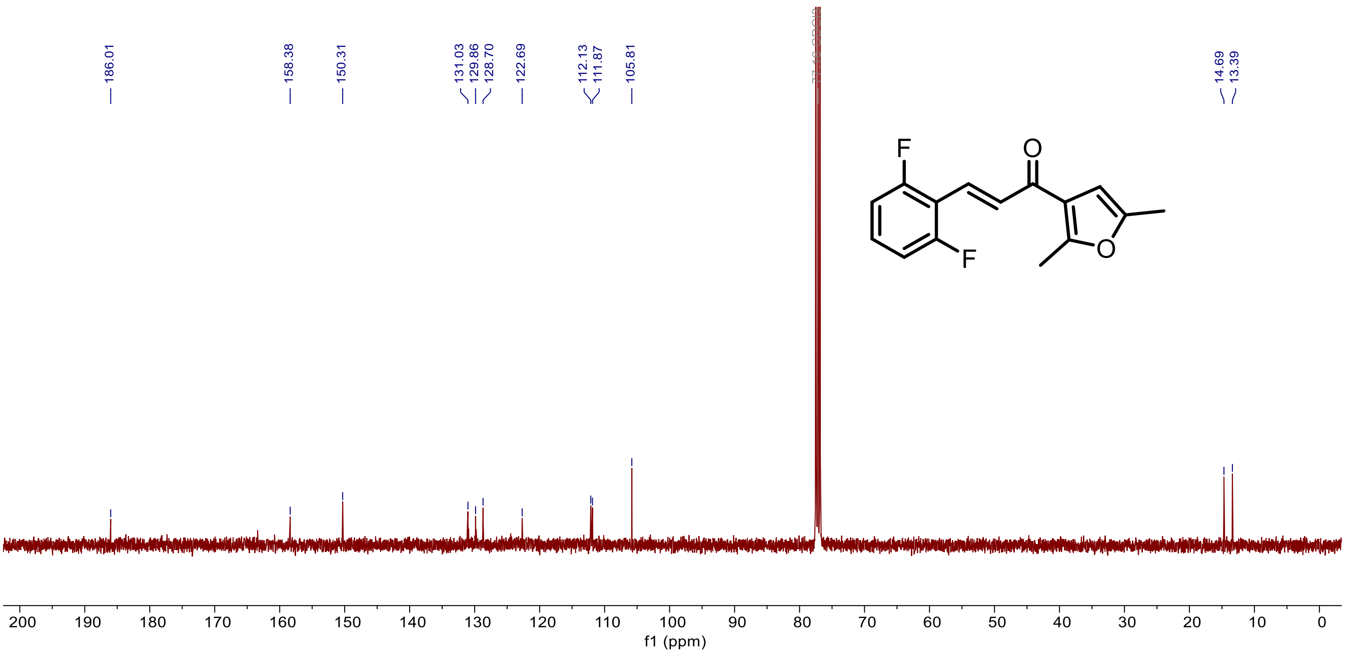

(E)-3-(4-(benzyloxy)phenyl)-1-(2,5-dimethylfuran-3-yl)prop-2-en-1-one (S12)

^{1}H NMR (400 MHz, CDCl$_3$)

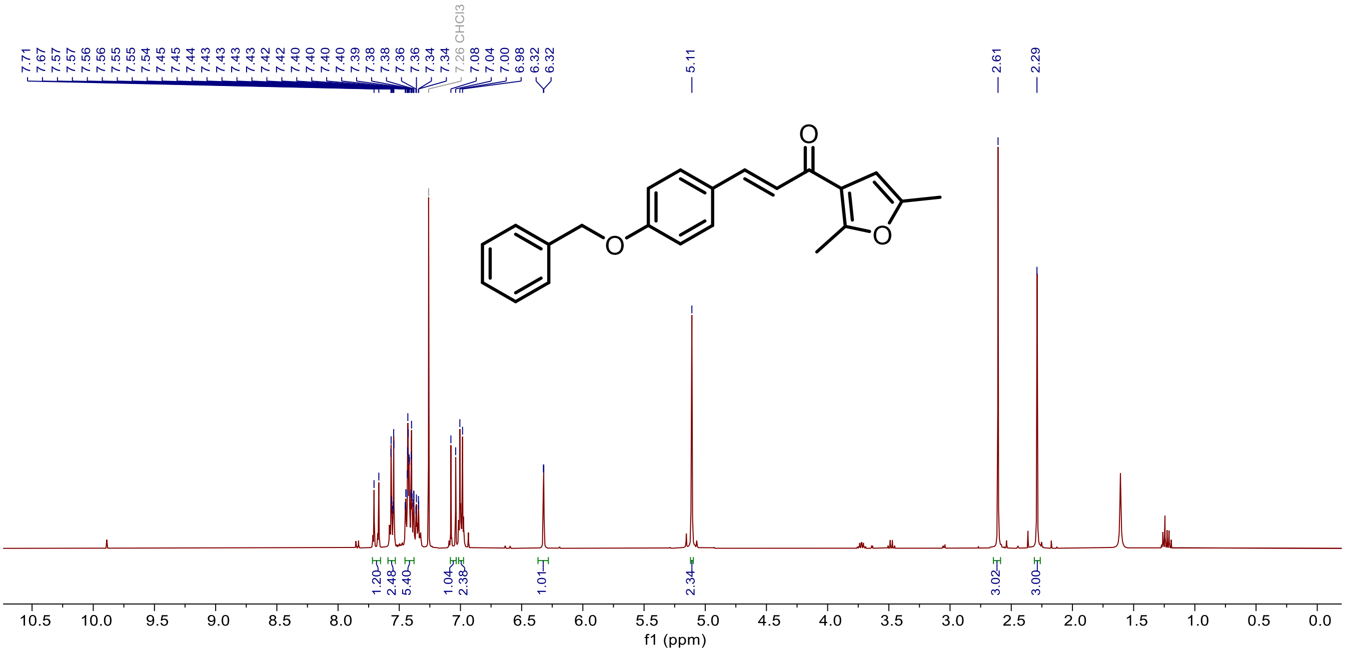

^{13}C NMR (101 MHz, $CDCl_3$)

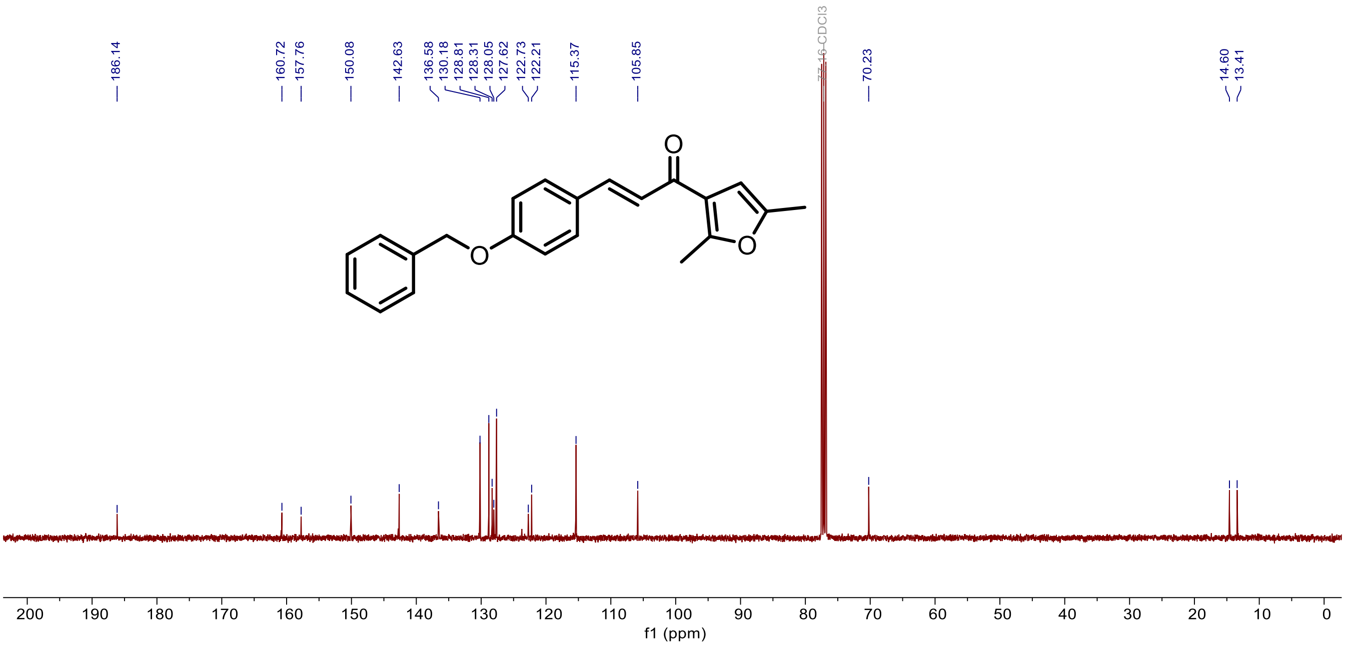

(E)-1-(2,5-dimethylfuran-3-yl)-3-phenylprop-2-en-1-one (S13)

^{1}H NMR (400 MHz, $CDCl_3$)

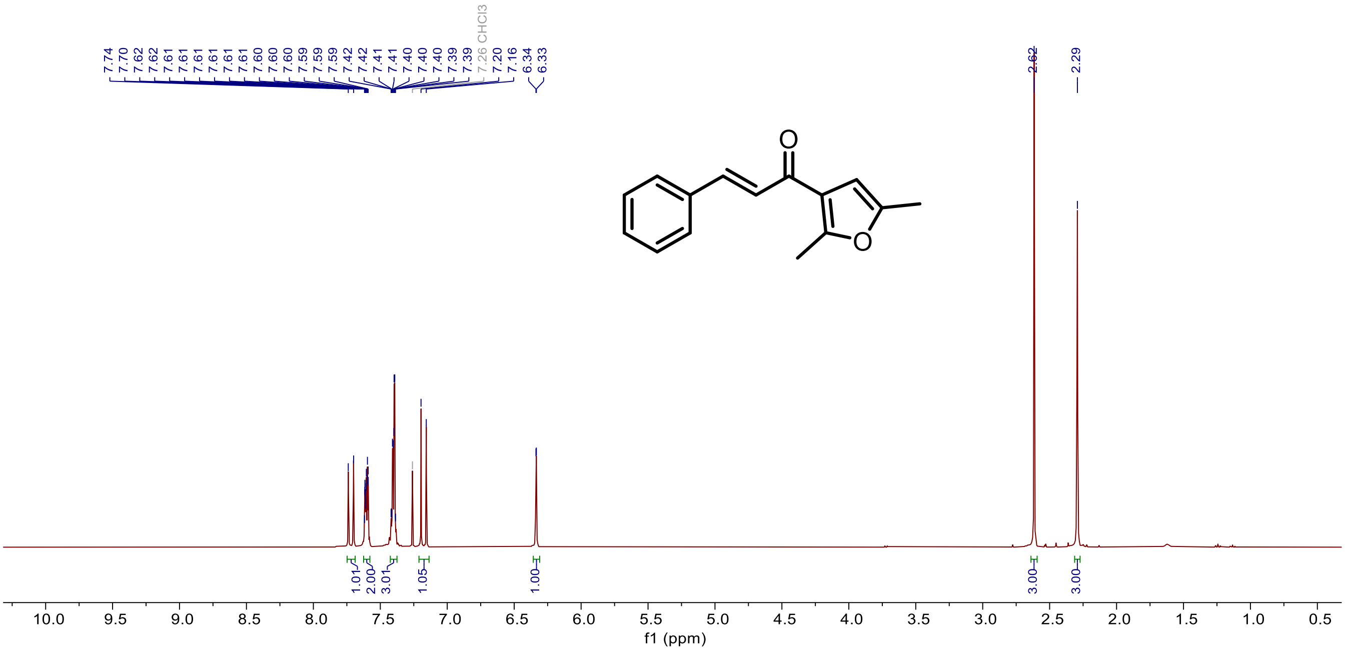

^{13}C NMR (101 MHz, $CDCl_3$)

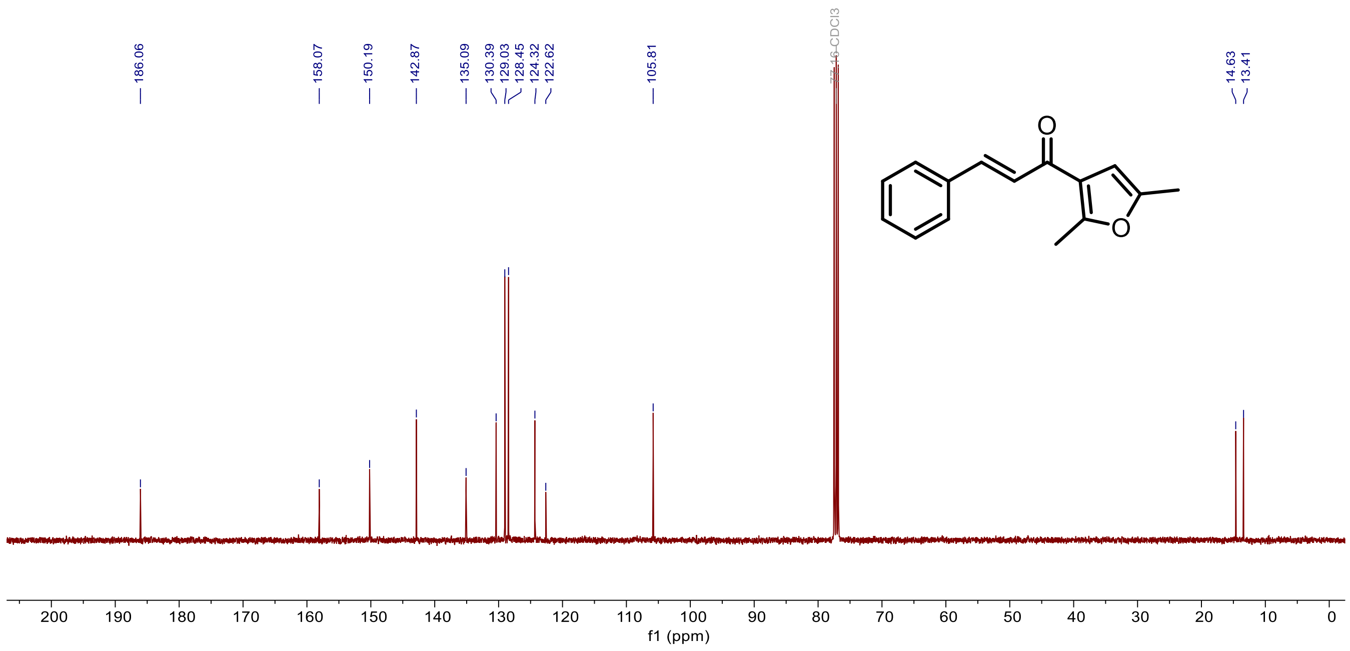

(E)-3-(4-chlorophenyl)-1-phenylprop-2-en-1-one (S14)

^1H NMR (500 MHz, $CDCl_3$)

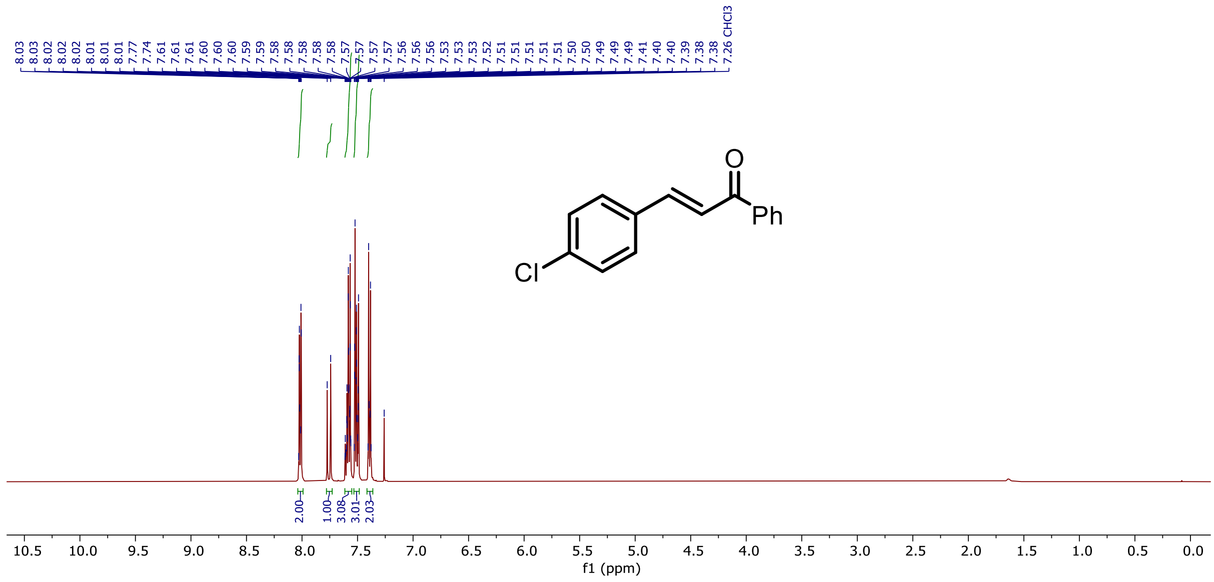

^{13}C NMR (126 MHz, $CDCl_3$)

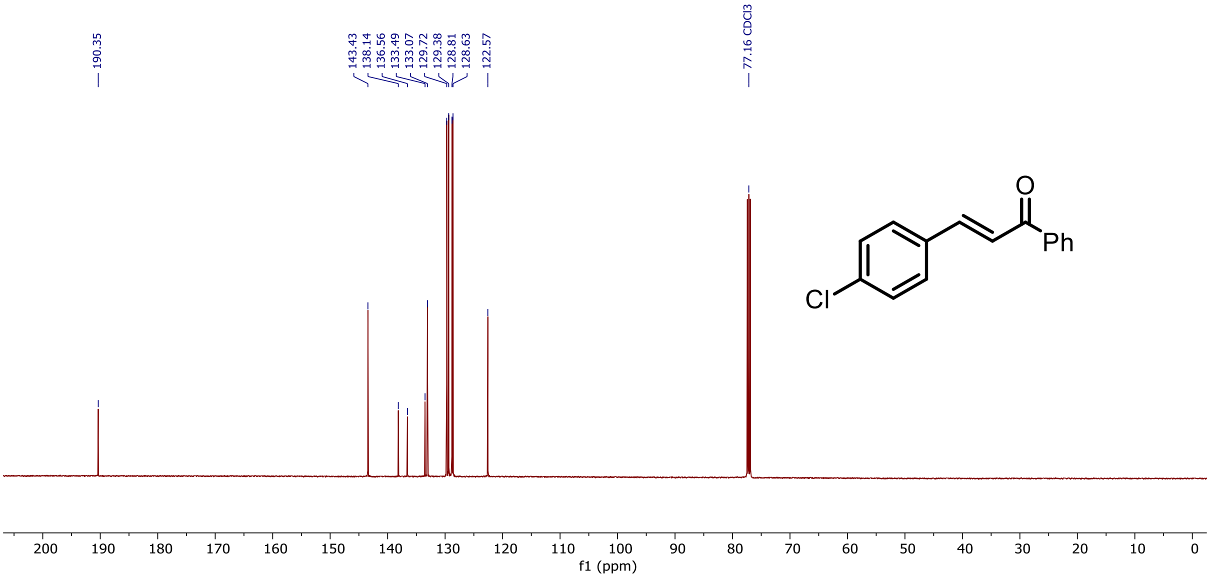

1,3-diphenylpropan-1-one (2a)

^{1}H NMR (500 MHz, $CDCl_3$)

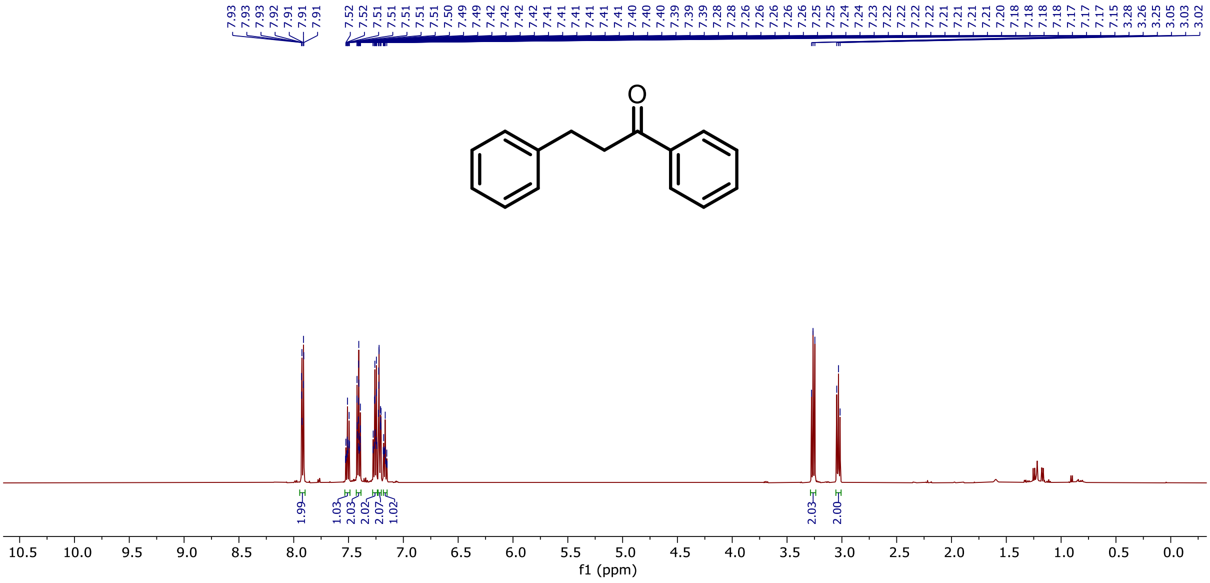

^{13}C NMR (126 MHz, $CDCl_3$)

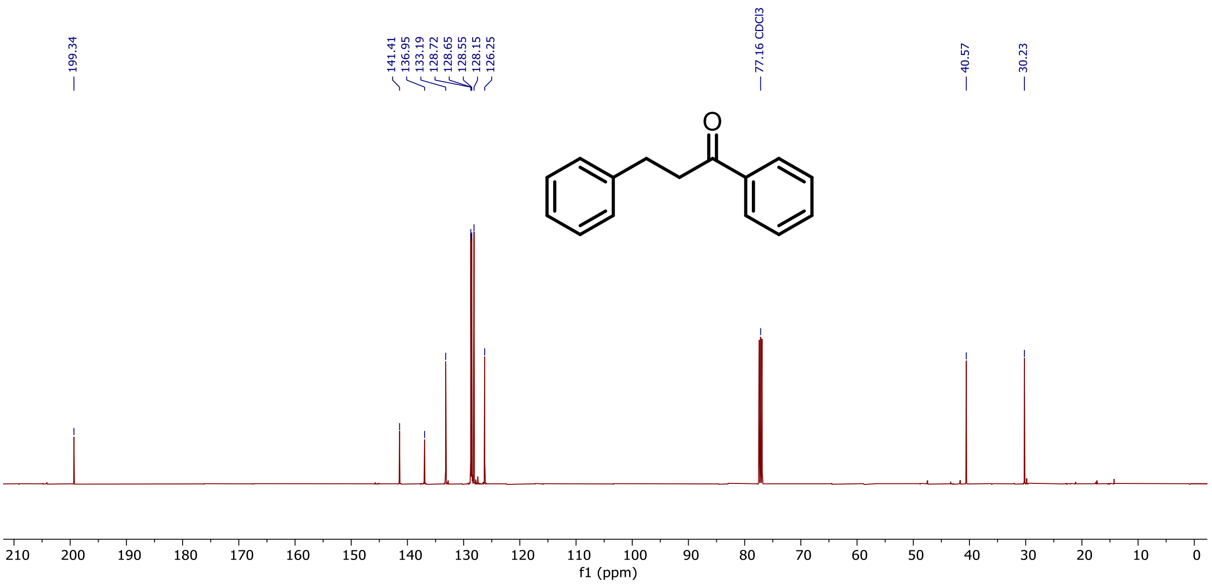

3-phenyl-1-(p-tolyl)propan-1-one (2b)

^{1}H NMR (400 MHz, $CDCl_3$)

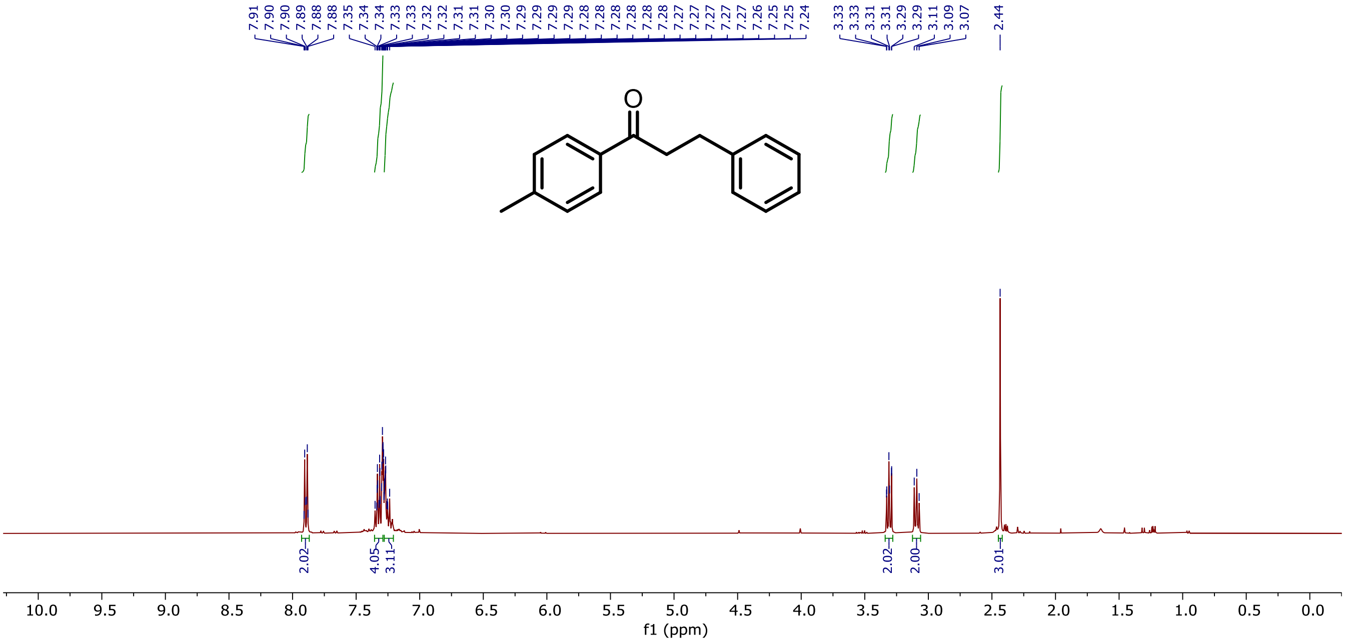

^{13}C NMR (101 MHz, CDCl$_3$)

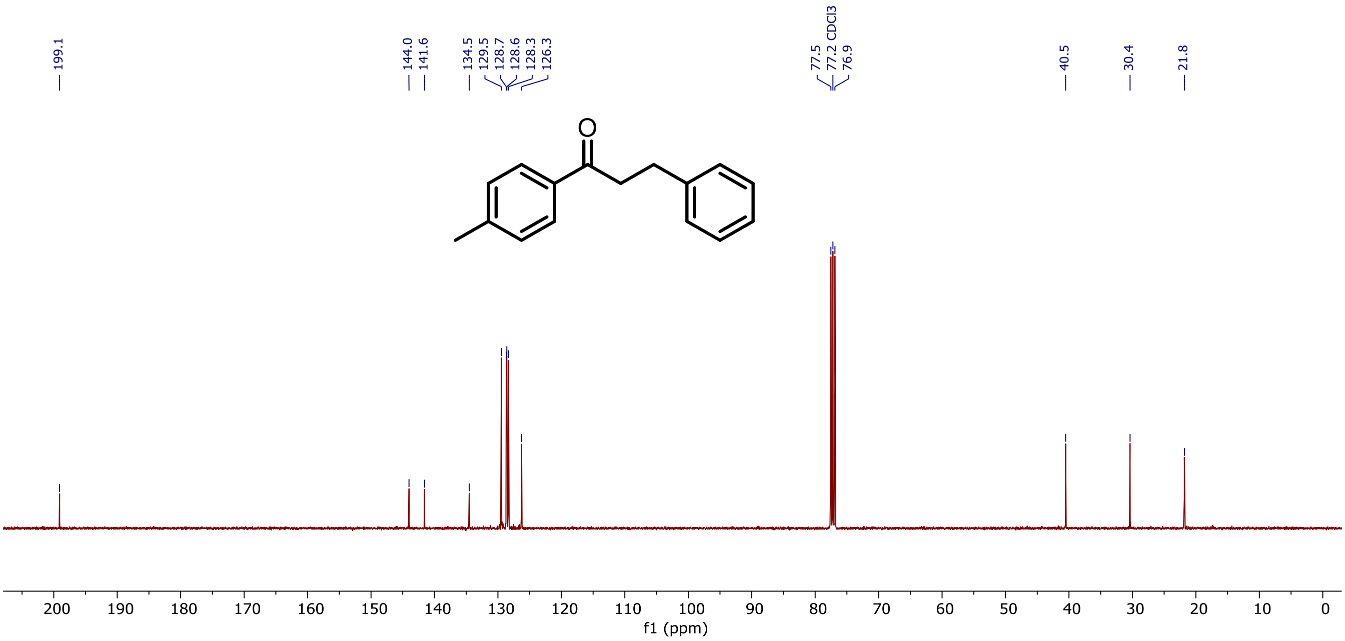

1-(4-(benzyloxy)phenyl)-3-phenylpropan-1-one (2c)

^{1}H NMR (400 MHz, CDCl$_3$)

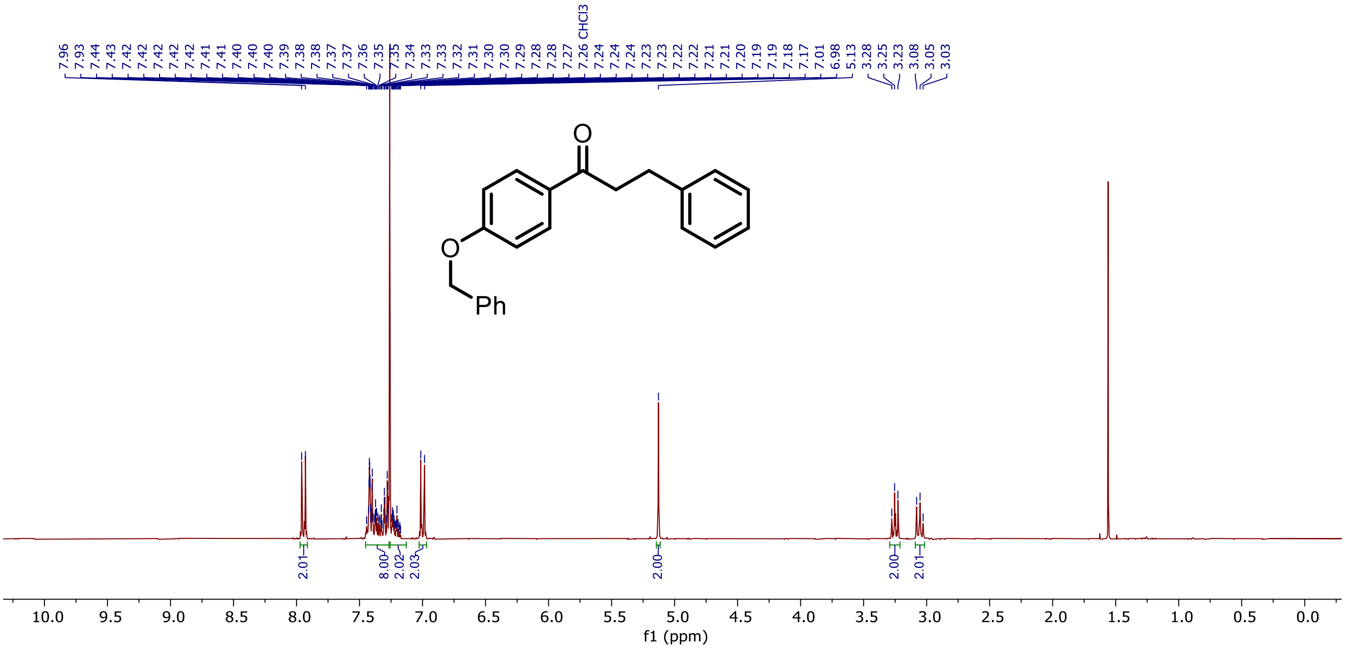

^{13}C NMR (101 MHz, CDCl$_3$)

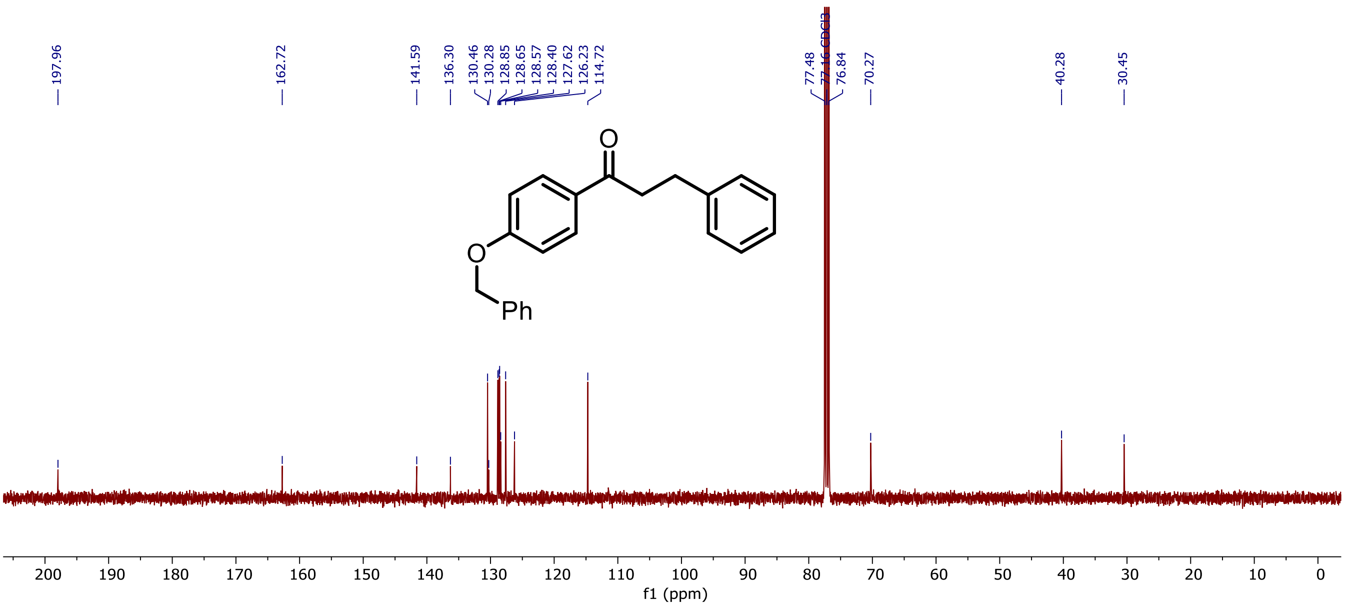

1-([1,1'-biphenyl]-4-yl)-3-phenylpropan-1-one (2d)

^{1}H NMR (400 MHz, CDCl$_3$)

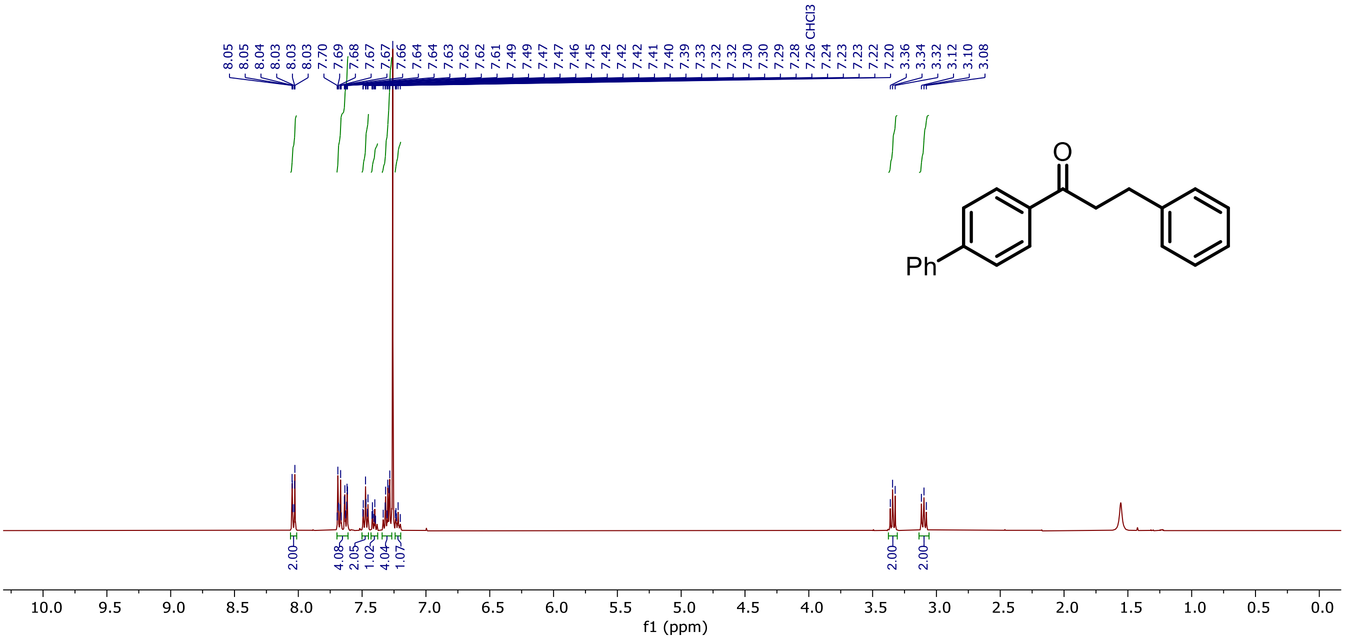

^{13}C NMR (101 MHz, $CDCl_3$)

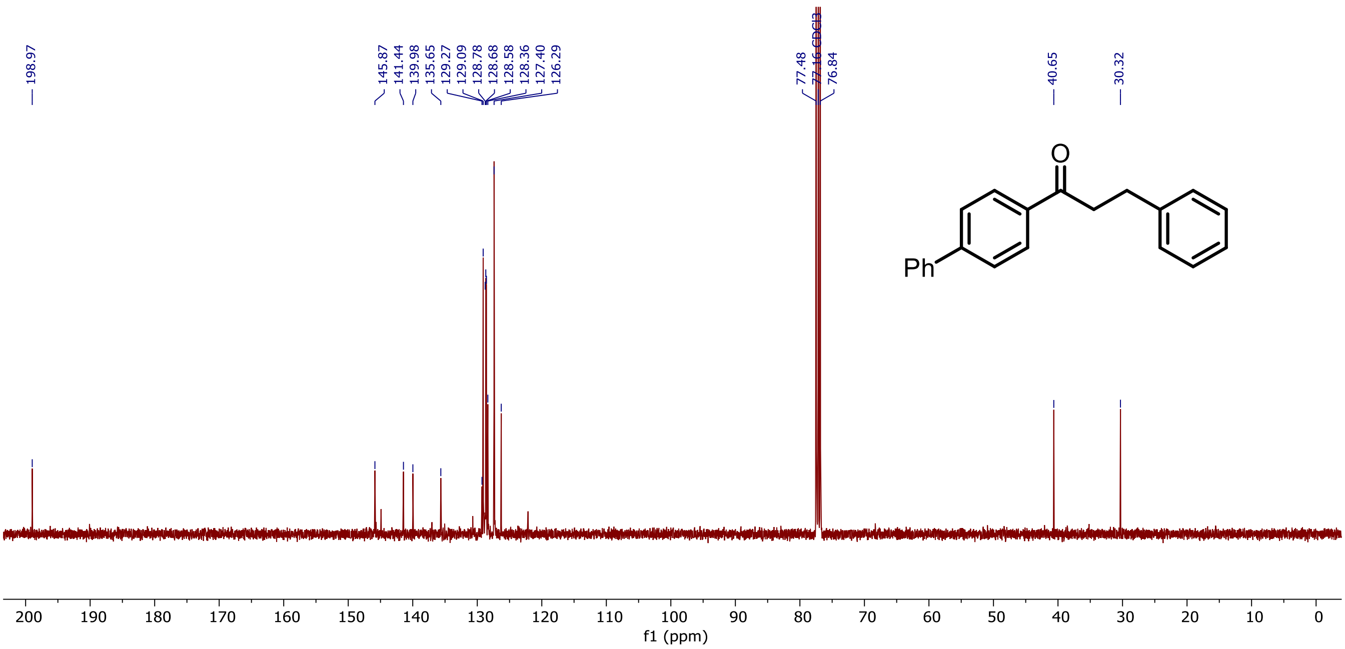

3-(benzo[d][1,3]dioxol-5-yl)-1-(p-tolyl)propan-1-one (2e)

^{1}H NMR (400 MHz, $CDCl_3$)

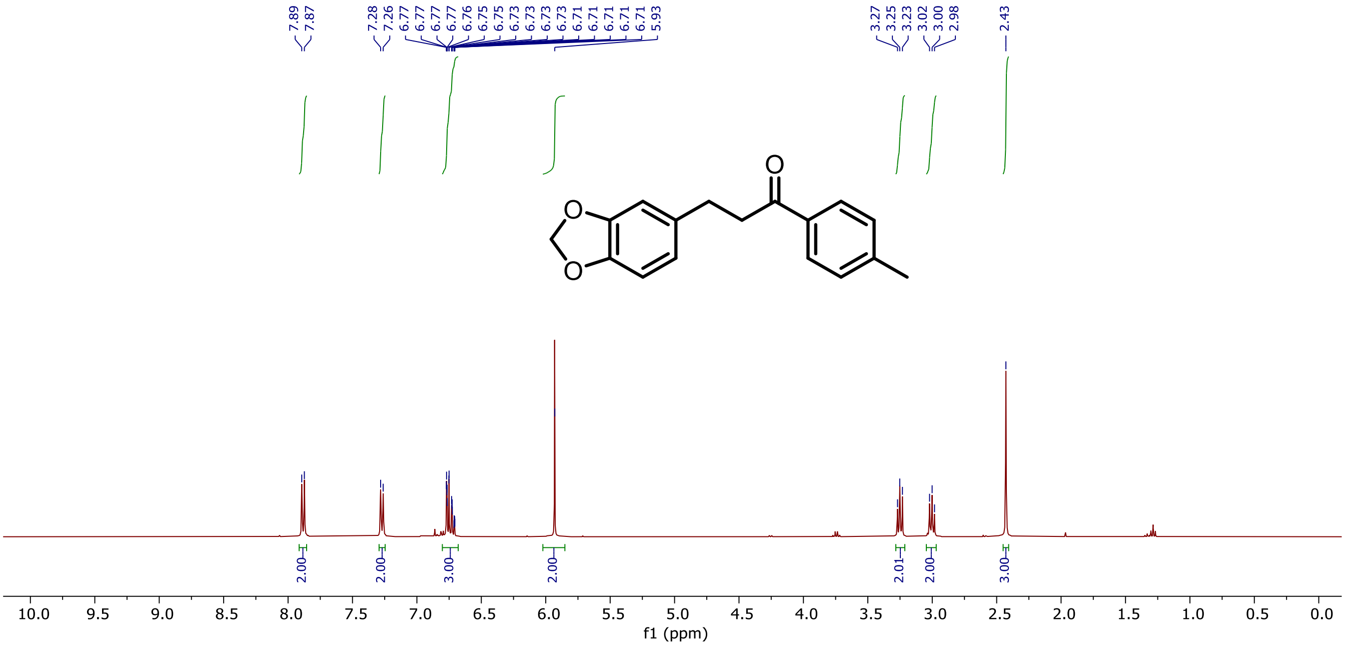

^{13}C NMR (101 MHz, $CDCl_3$)

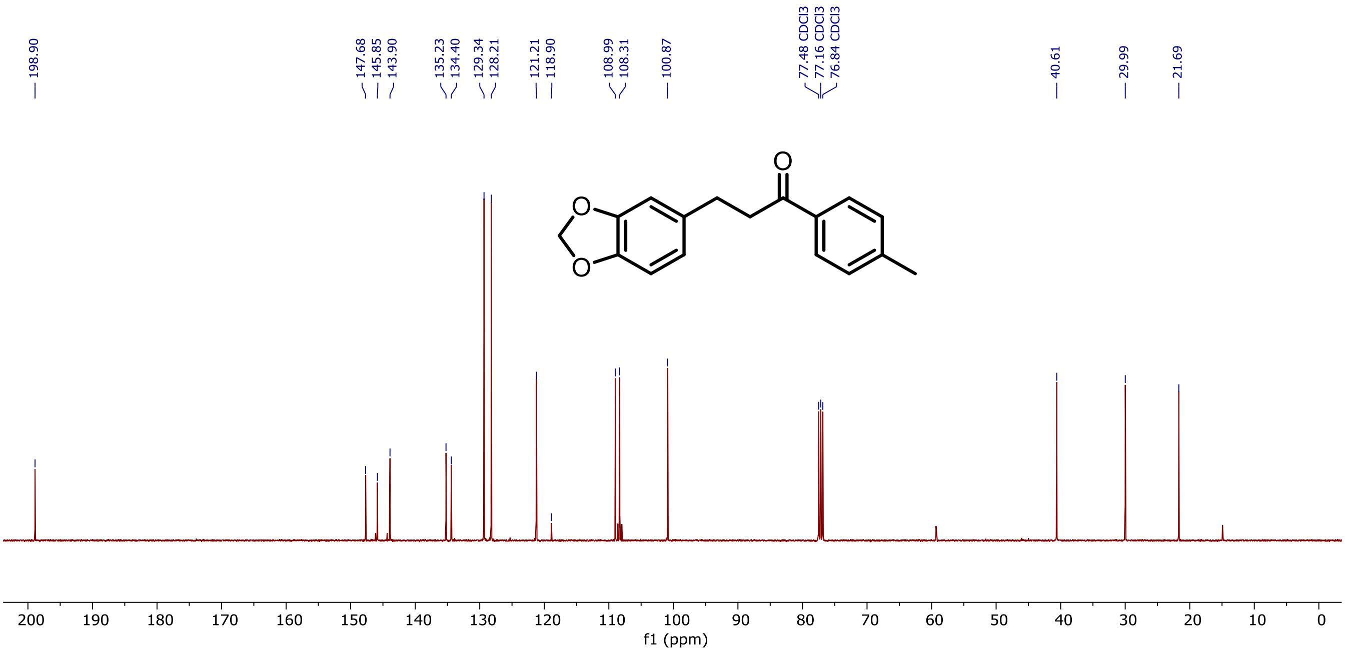

1-(2-fluorophenyl)-3-(naphthalen-2-yl)propan-1-one (2f)

^{1}H NMR (400 MHz, $CDCl_3$)

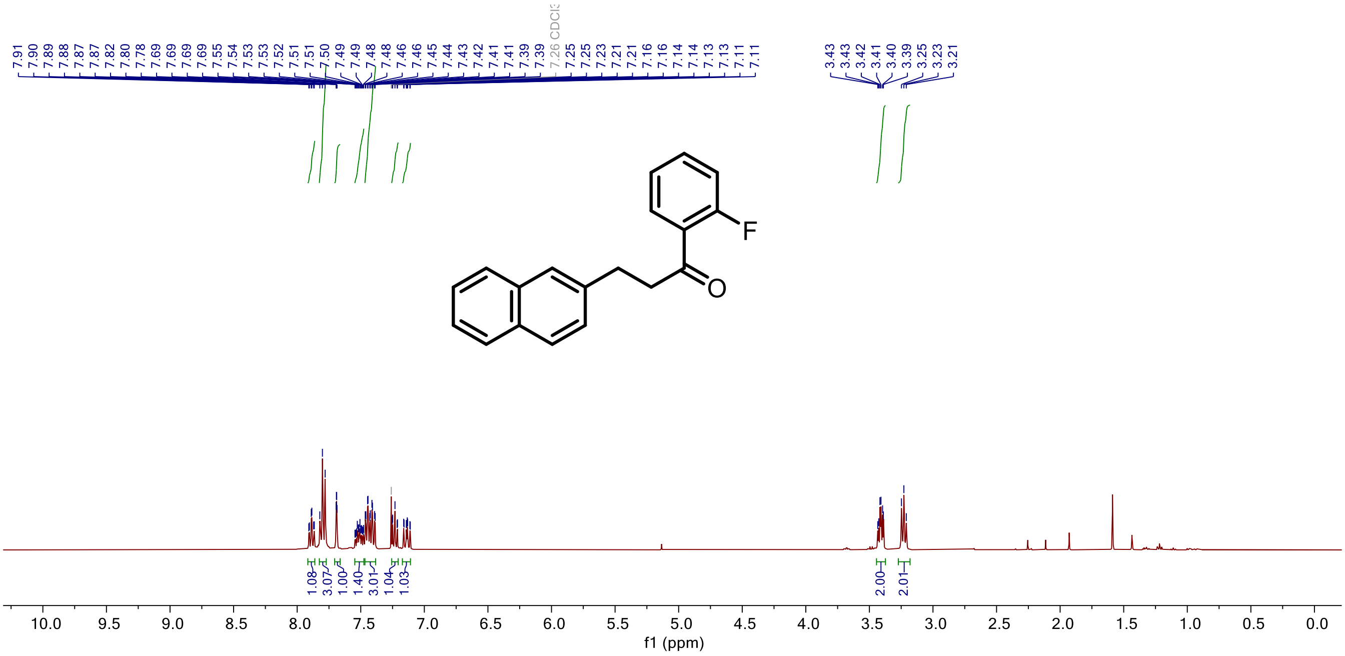

^{13}C NMR (101 MHz, $CDCl_3$)

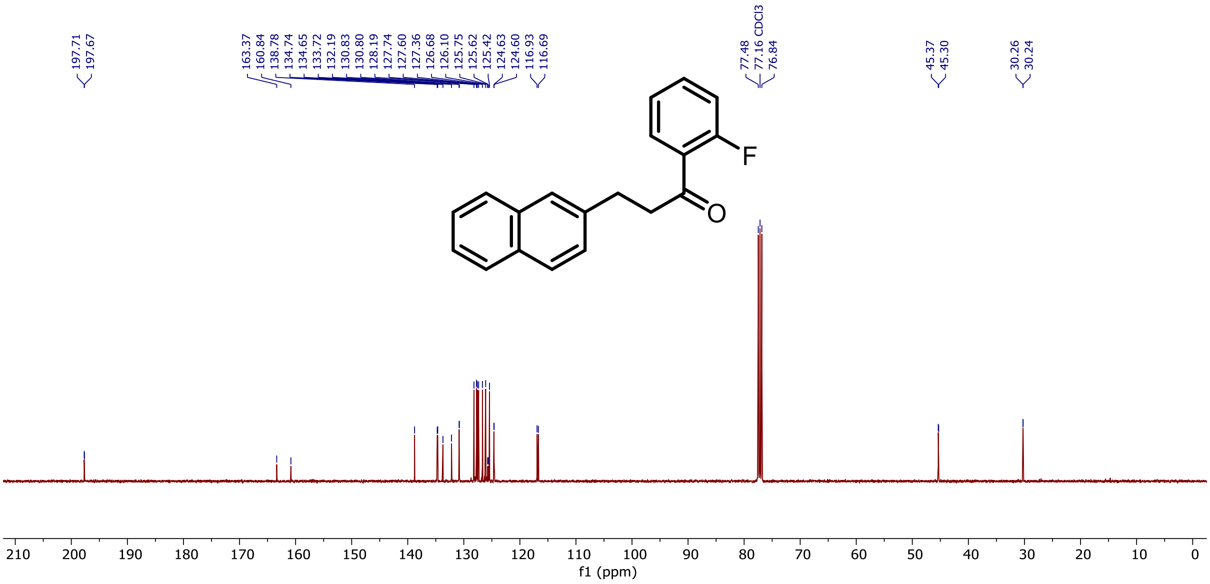

^{19}F NMR (376 MHz, $CDCl_3$)

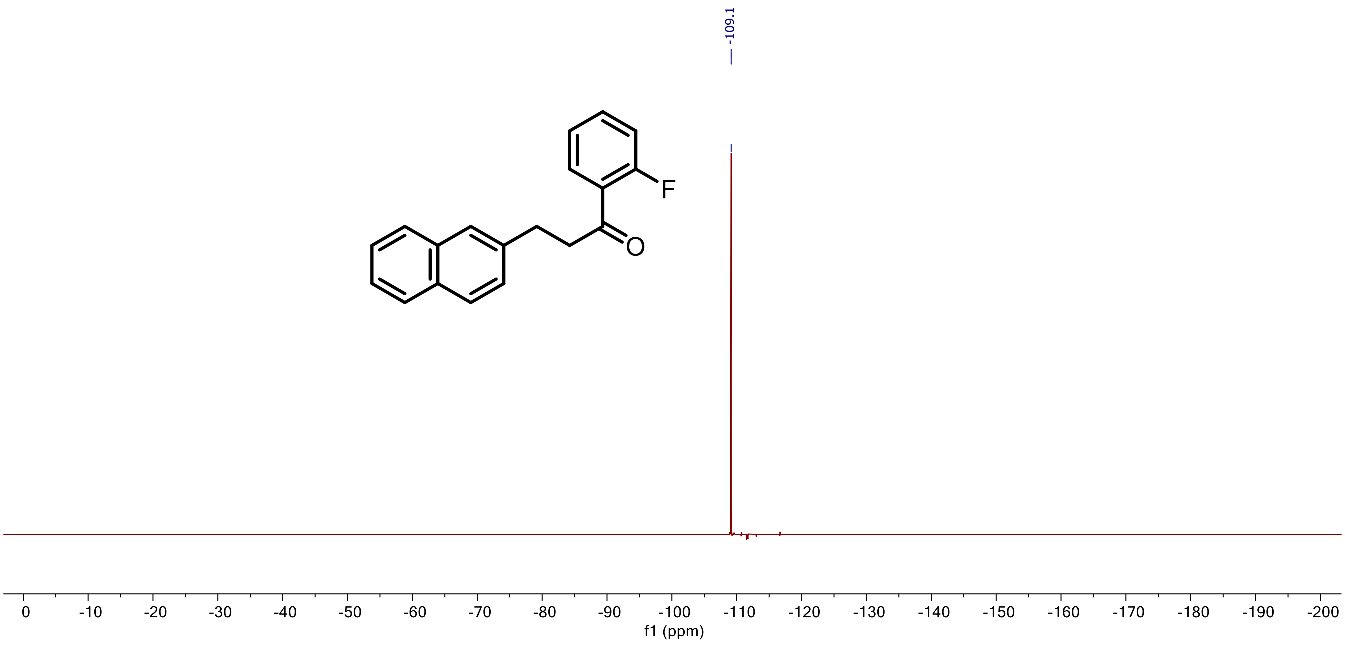

3-(2-bromo-4,5-dimethoxyphenyl)-1-(p-tolyl)propan-1-one (2g)

^{1}H NMR (400 MHz, $CDCl_3$)

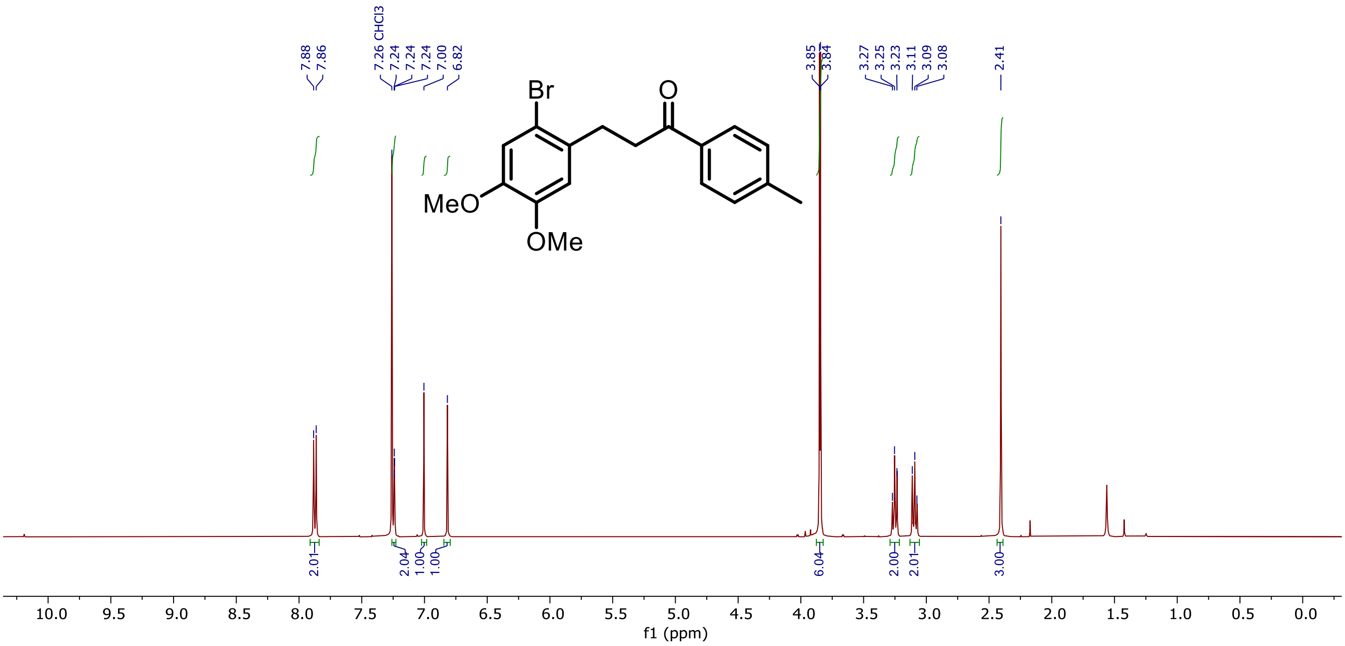

^{13}C NMR (101 MHz, $CDCl_3$)

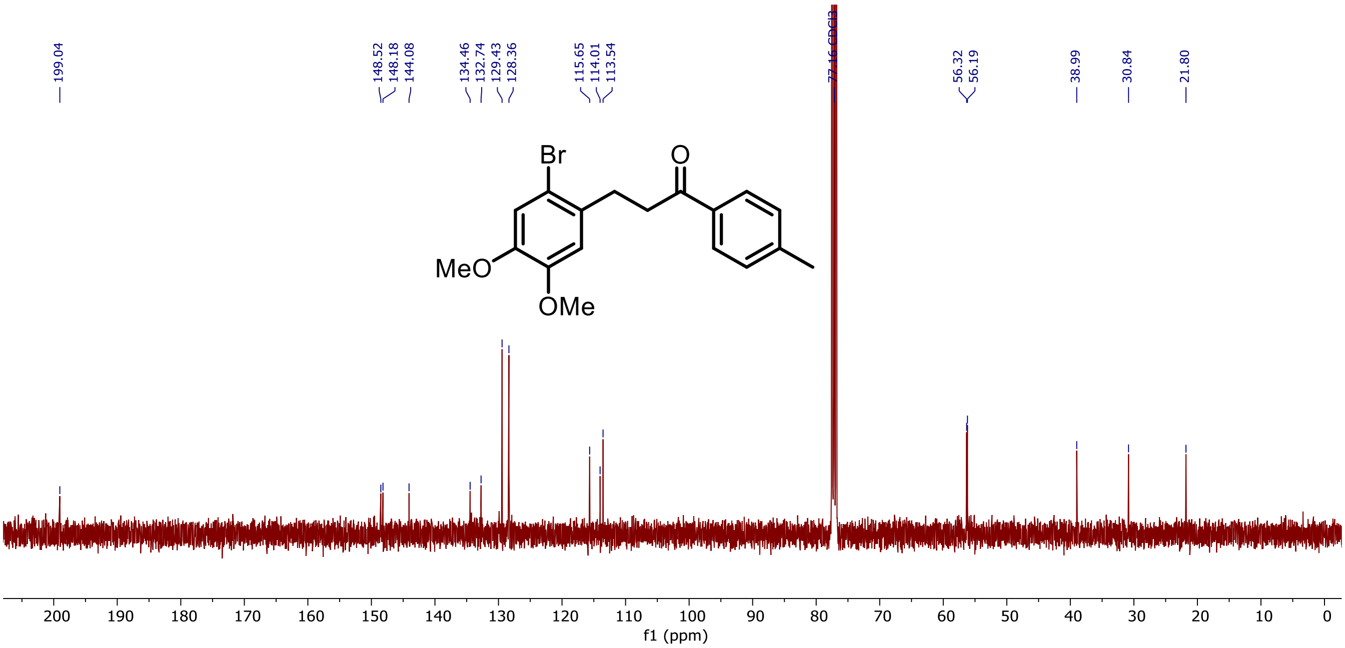

3-(2-ethynylphenyl)-1-(p-tolyl)propan-1-one (2h)

^{1}H NMR (400 MHz, $CDCl_3$)

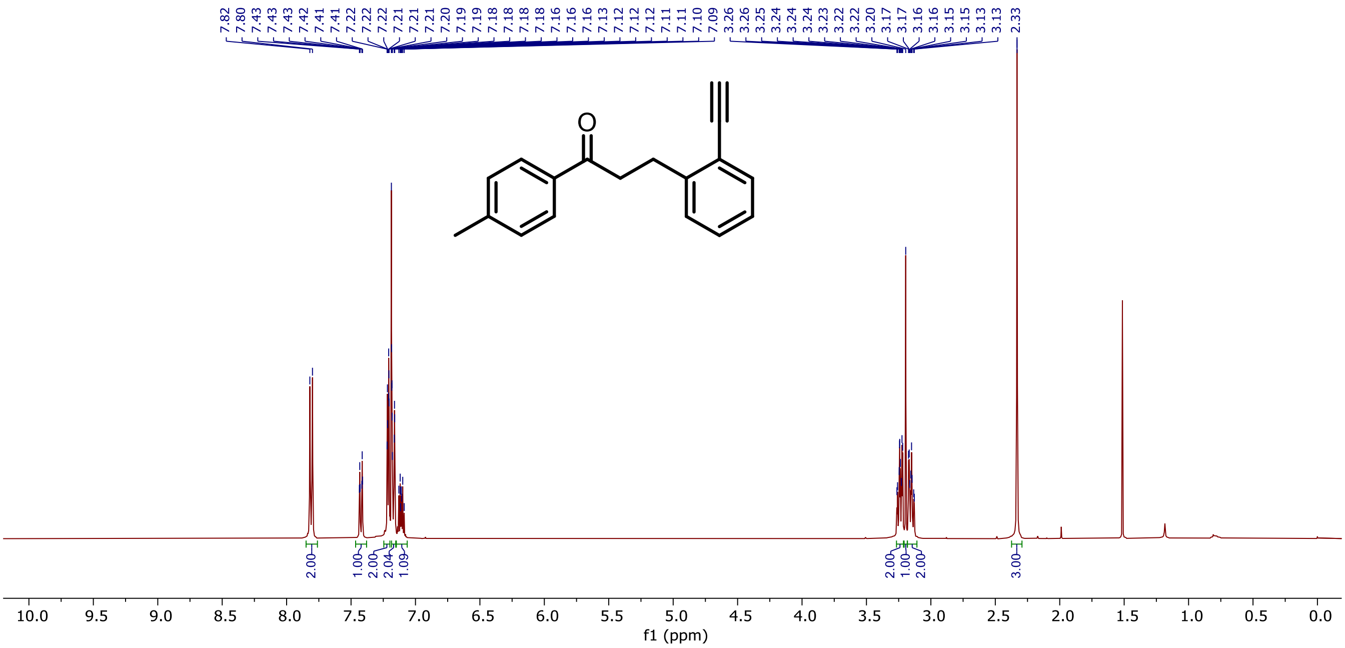

^{13}C NMR (101 MHz, $CDCl_3$)

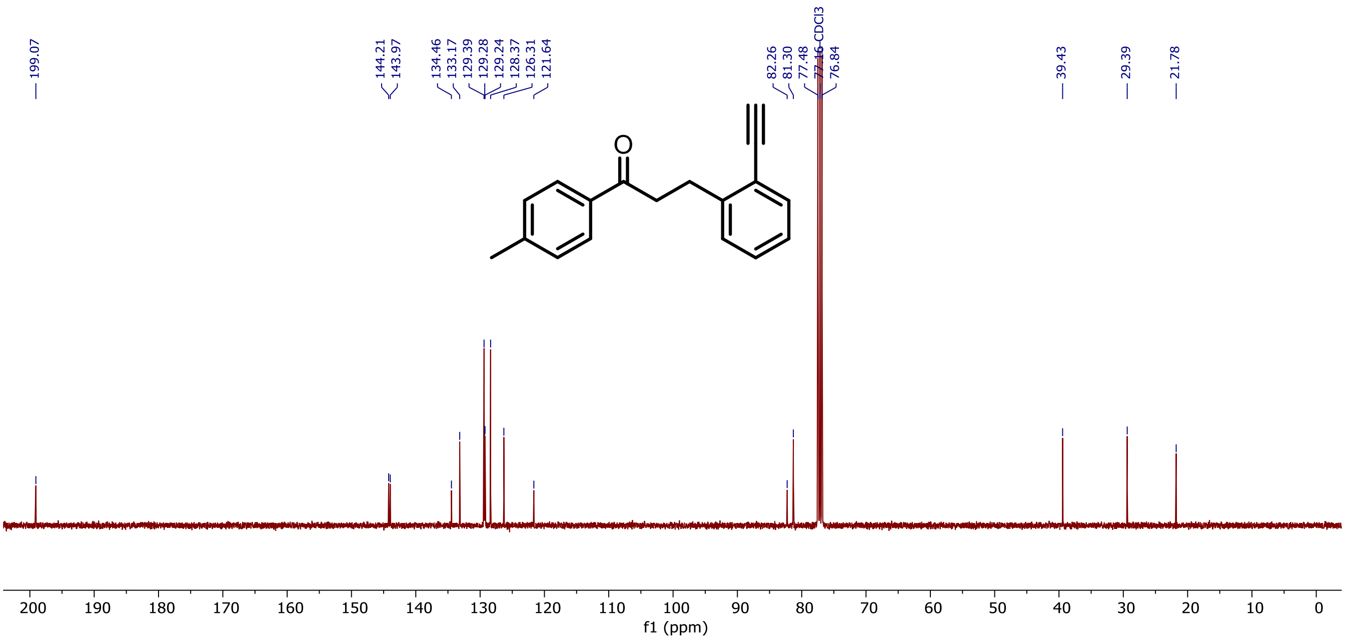

3-phenyl-1-(thiophen-3-yl)propan-1-one (2i)

^{1}H NMR (500 MHz, $CDCl_3$)

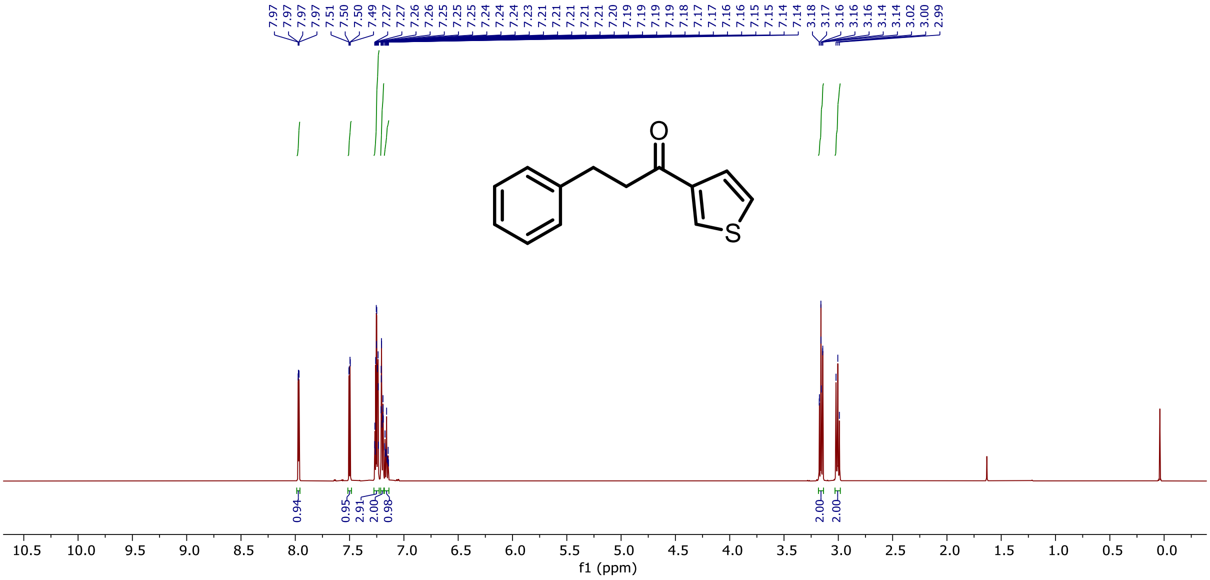

^{13}C NMR (126 MHz, $CDCl_3$)

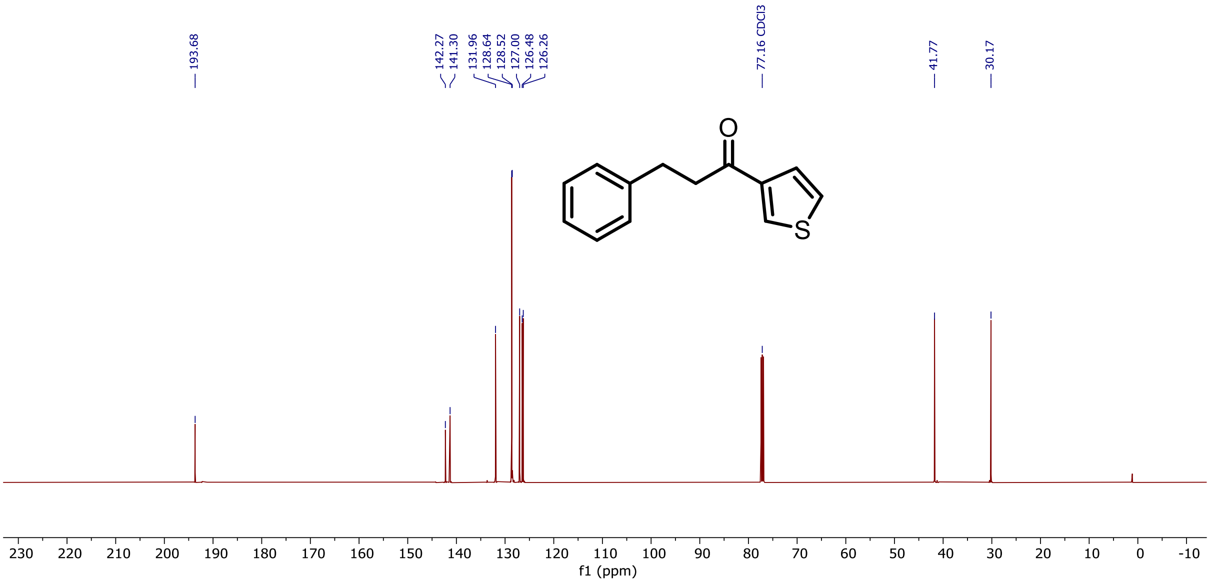

3-phenyl-1-(thiophen-2-yl)propan-1-one (2j)

^{1}H NMR (400 MHz, $CDCl_3$)

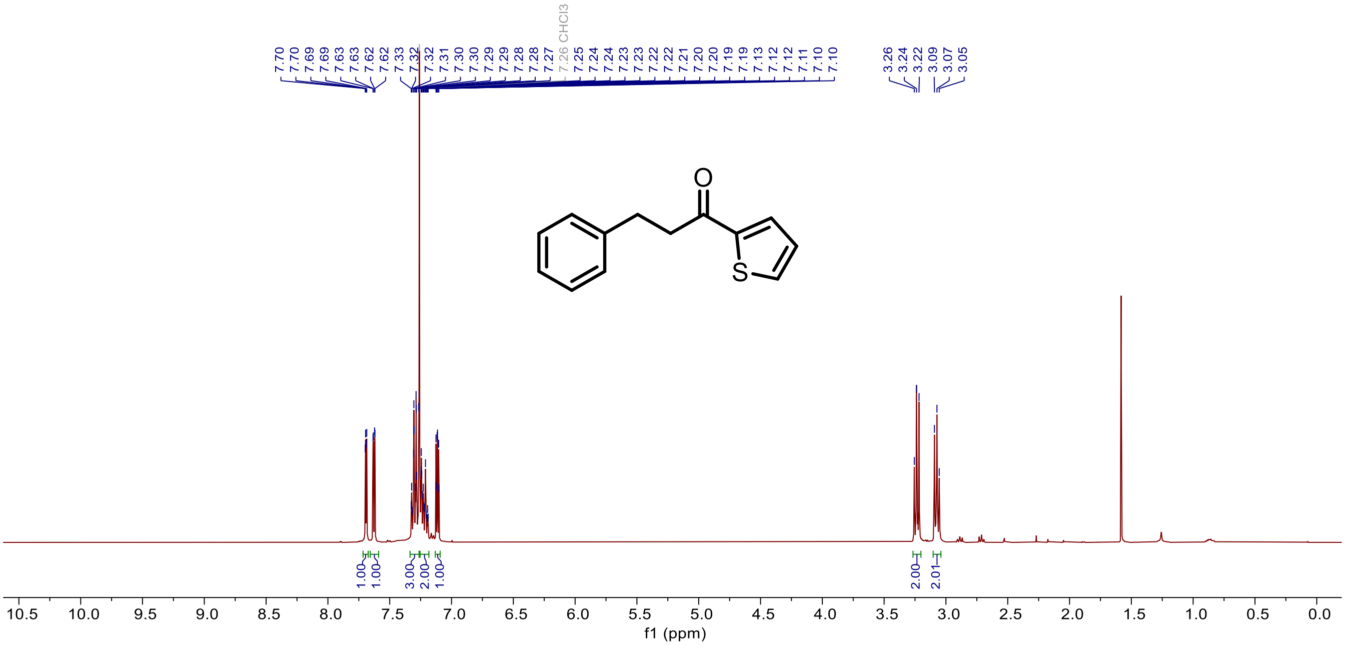

^{13}C NMR (101 MHz, $CDCl_3$)

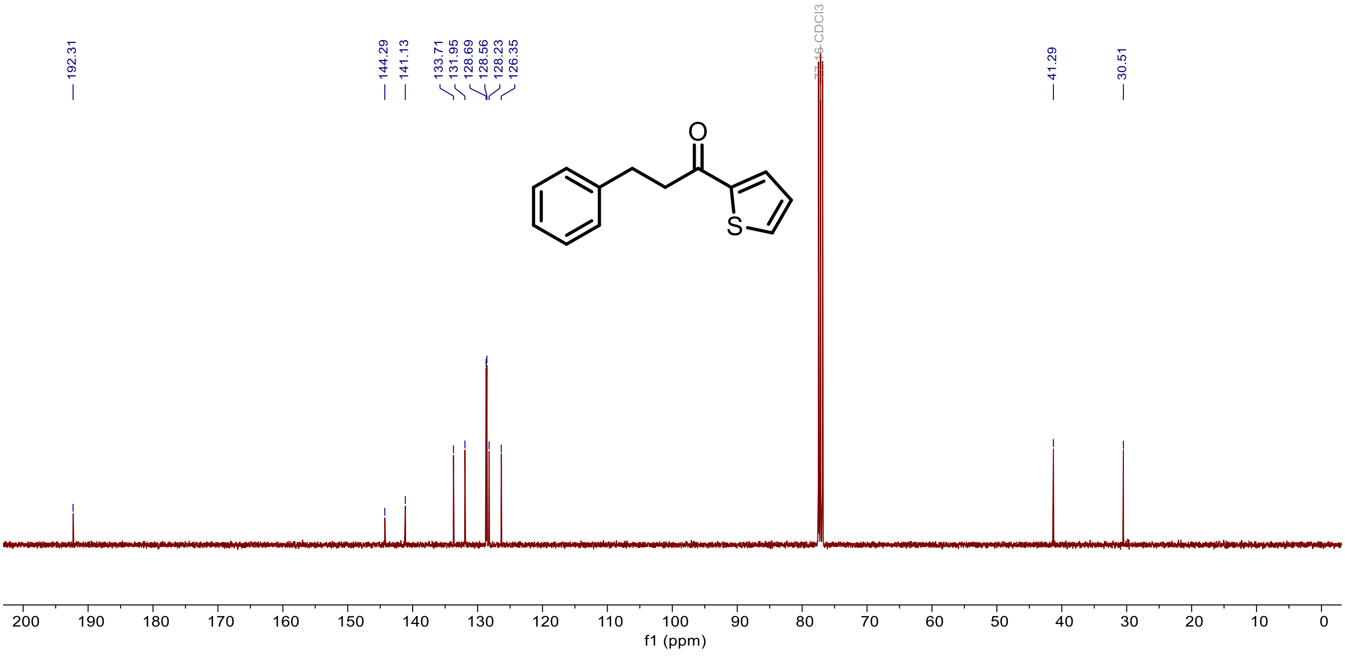

3-(2,6-difluorophenyl)-1-(2,5-dimethylfuran-3-yl)propan-1-one (2l)

^{1}H NMR (400 MHz, CDCl3)

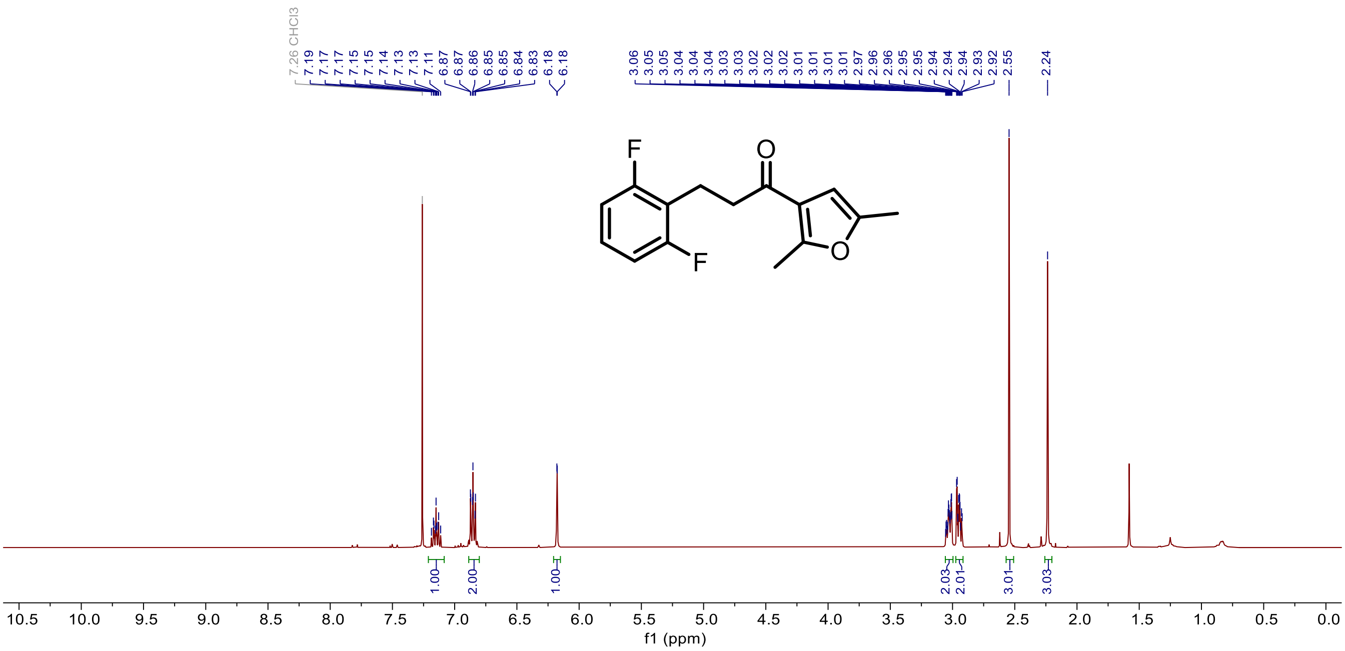

^{13}C NMR (101 MHz, CDCl3)

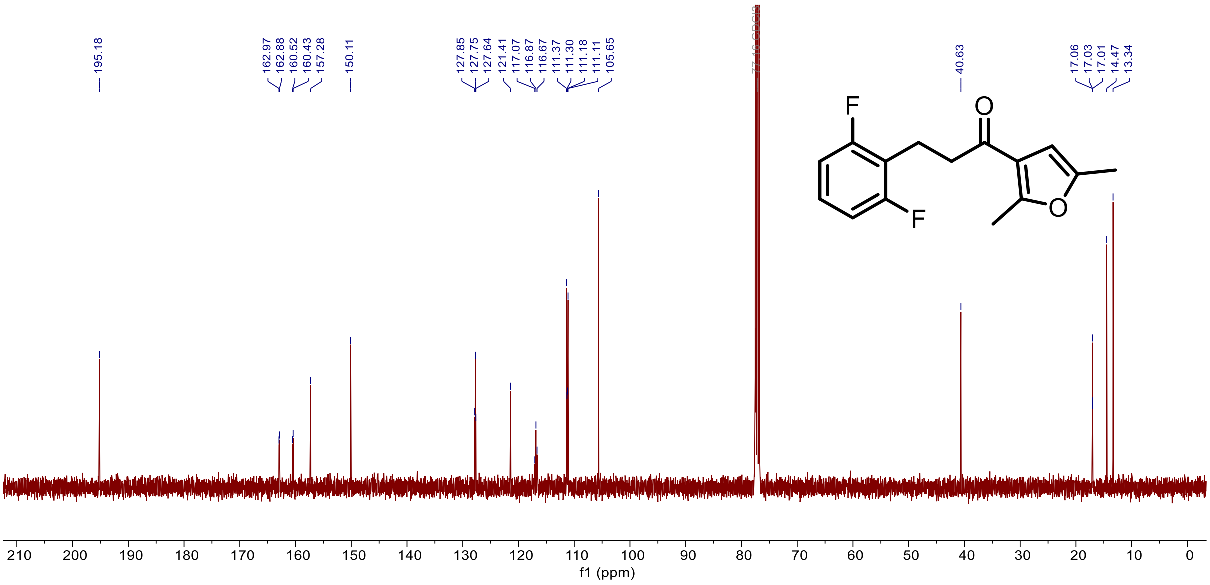

^{19}F NMR (376 MHz, CDCl₃)

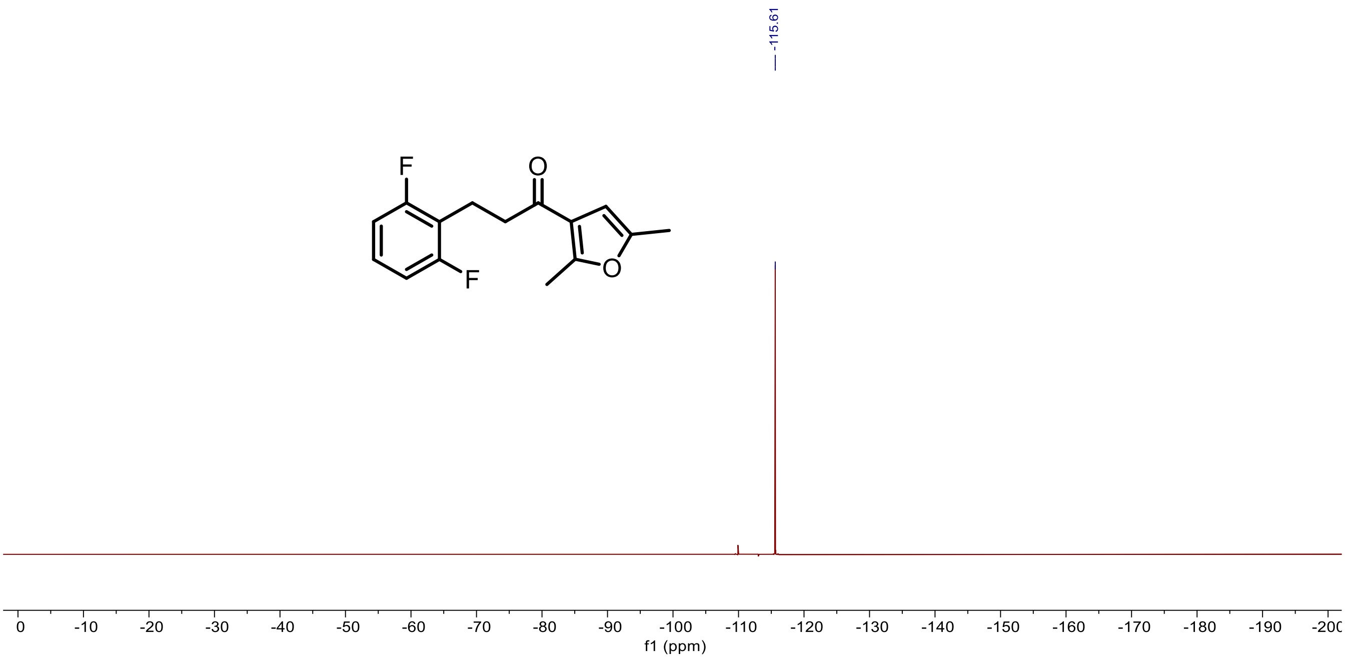

1-(2,5-dimethylfuran-3-yl)-3-phenylpropan-1-one (2k)

^{1}H NMR (400 MHz, CDCl₃)

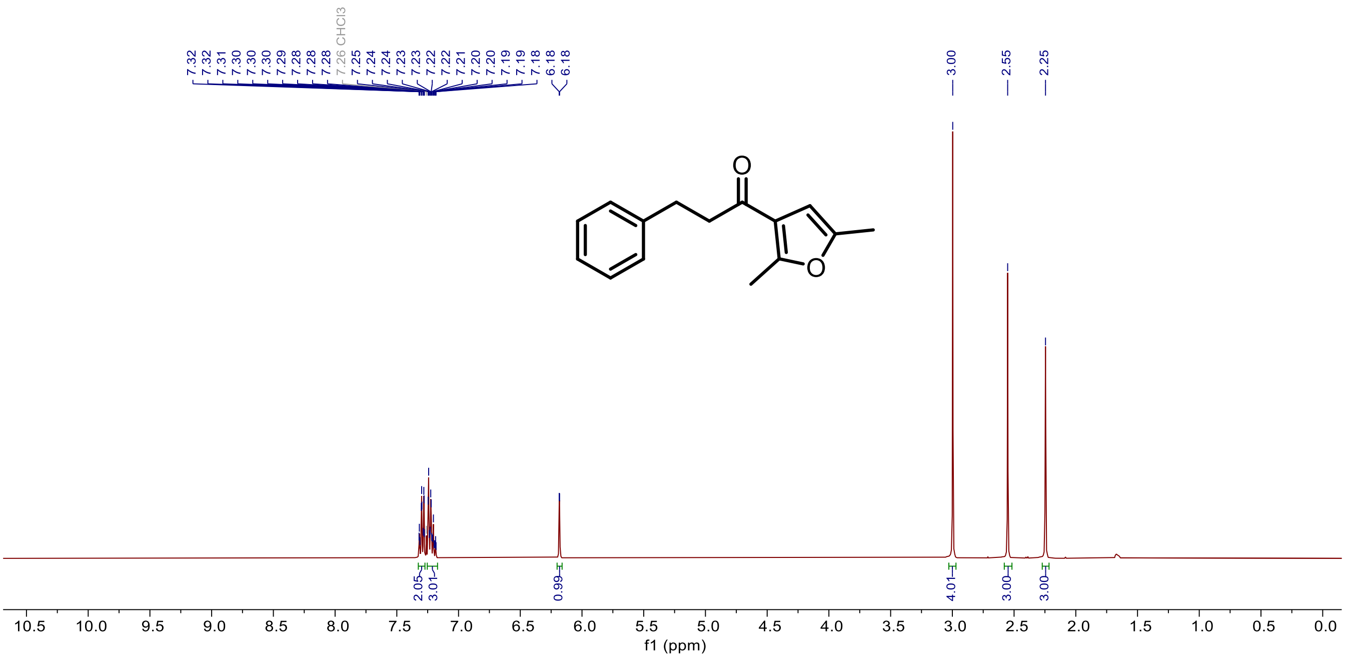

^{13}C NMR (101 MHz, CDCl$_3$)

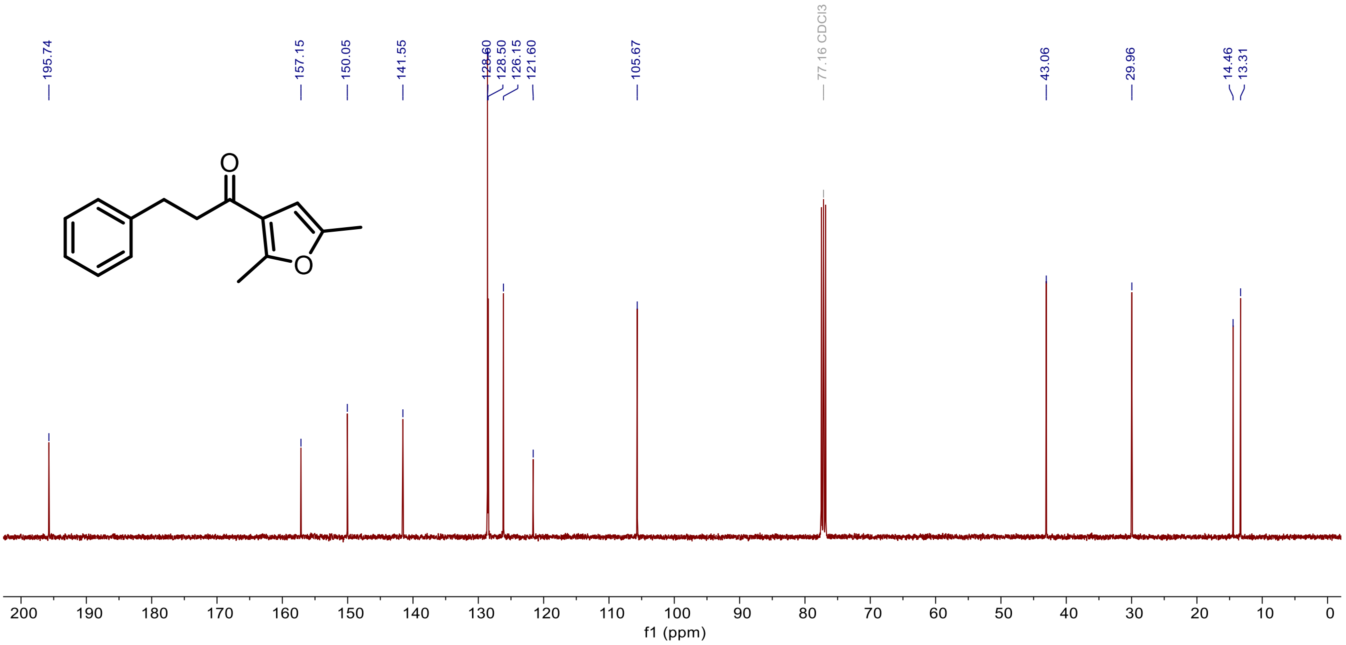

3-(4-(benzyloxy)phenyl)-1-(2,5-dimethylfuran-3-yl)propan-1-one (2m)

^{1}H NMR (400 MHz, CDCl$_3$)

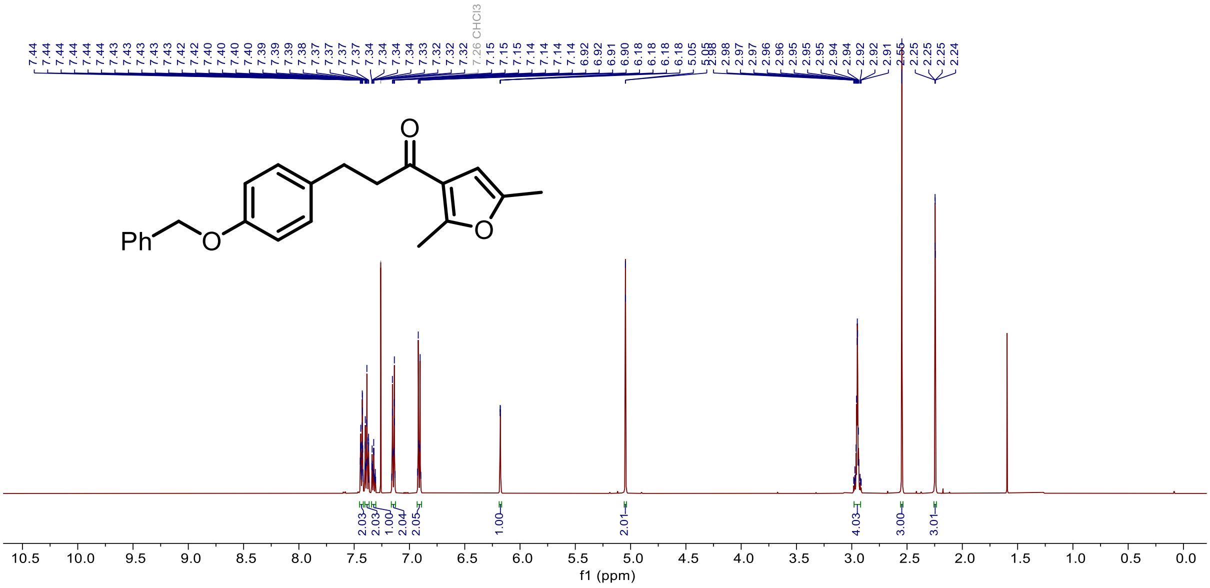

^{13}C NMR (101 MHz, CDCl$_3$)

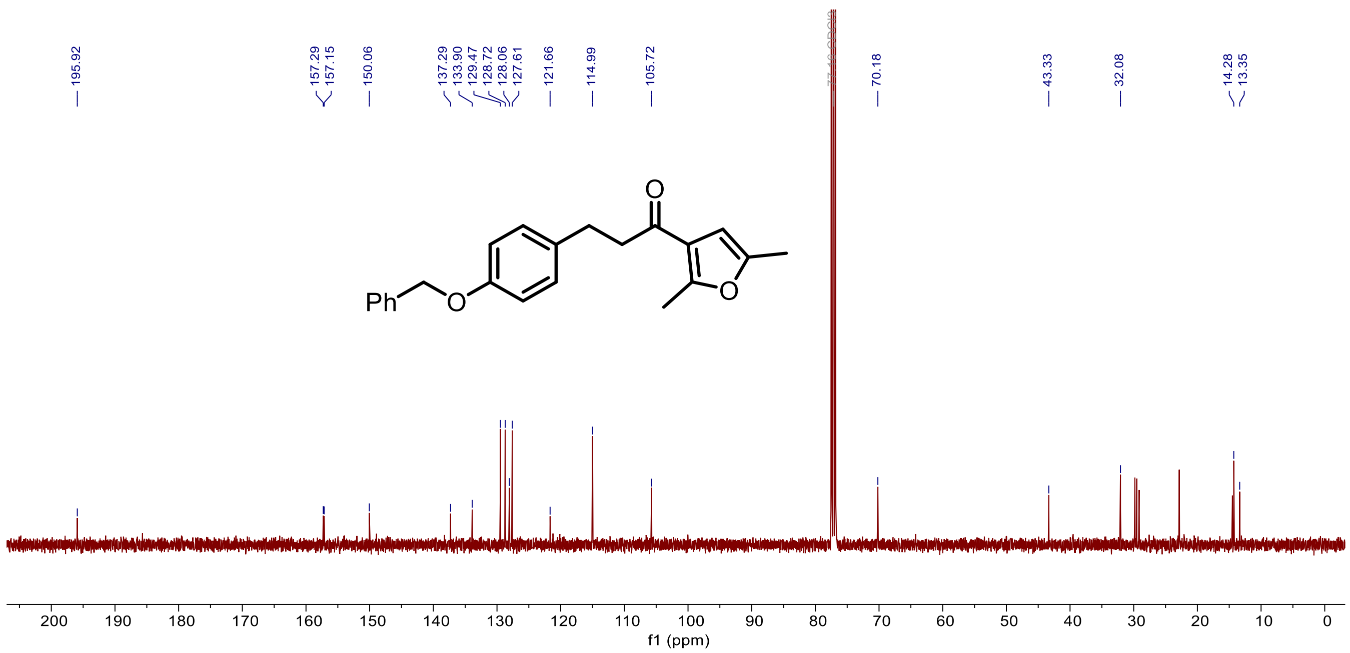

1-(2,5-dimethylfuran-3-yl)-3-(naphthalen-1-yl)propan-1-one (2n)

^{1}H NMR (400 MHz, CDCl$_3$)

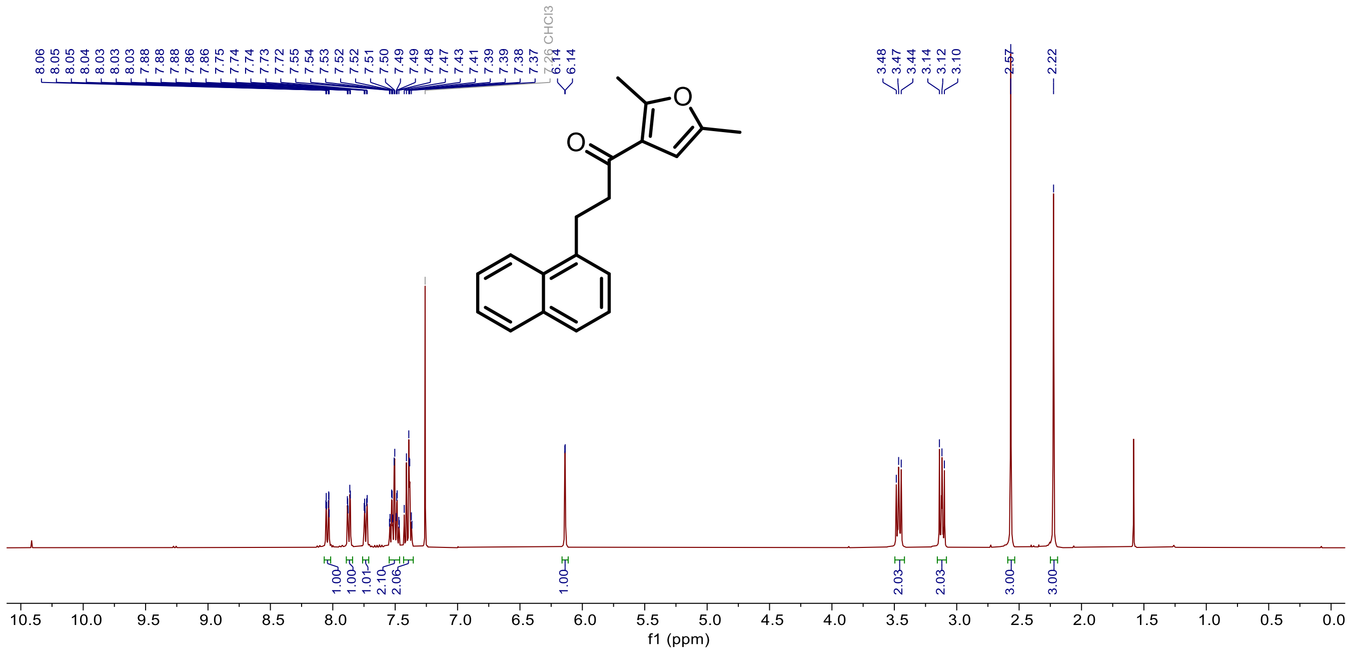

^{13}C NMR (101 MHz, $CDCl_3$)

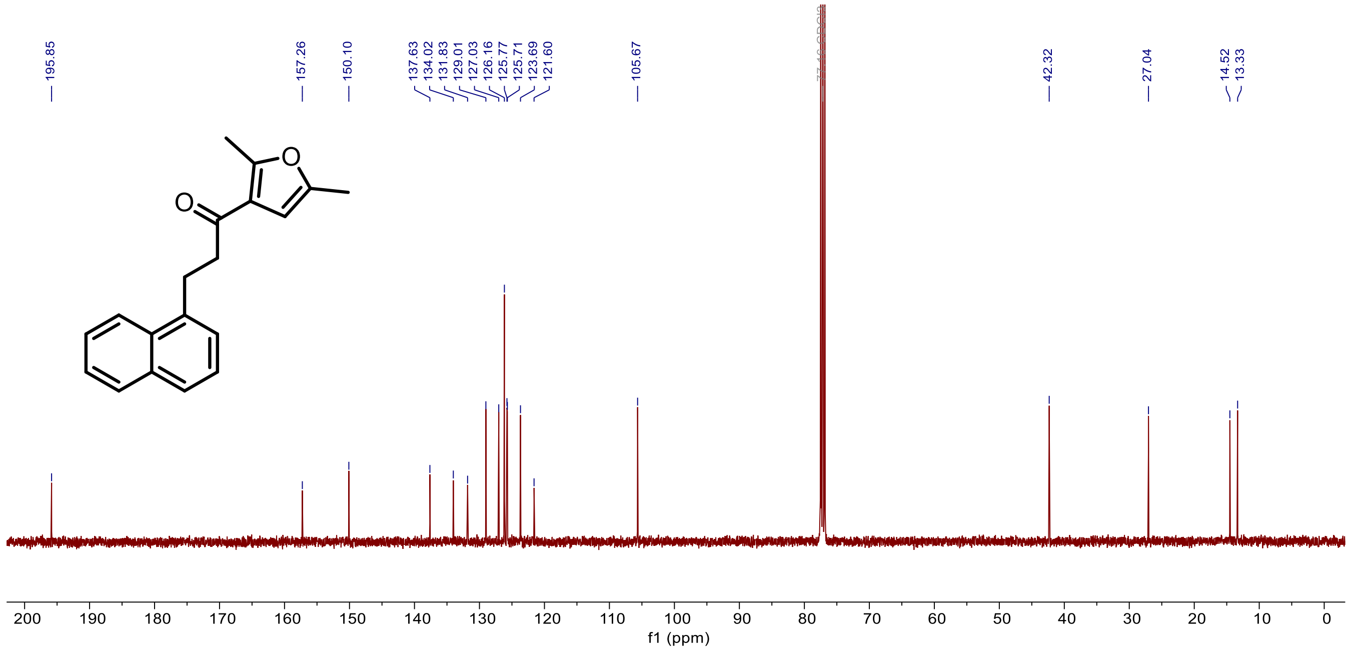

(E)-3-(4-chlorophenyl)-1-phenylprop-2-en-1-one (2o)

^{1}H NMR (500 MHz, $CDCl_3$)

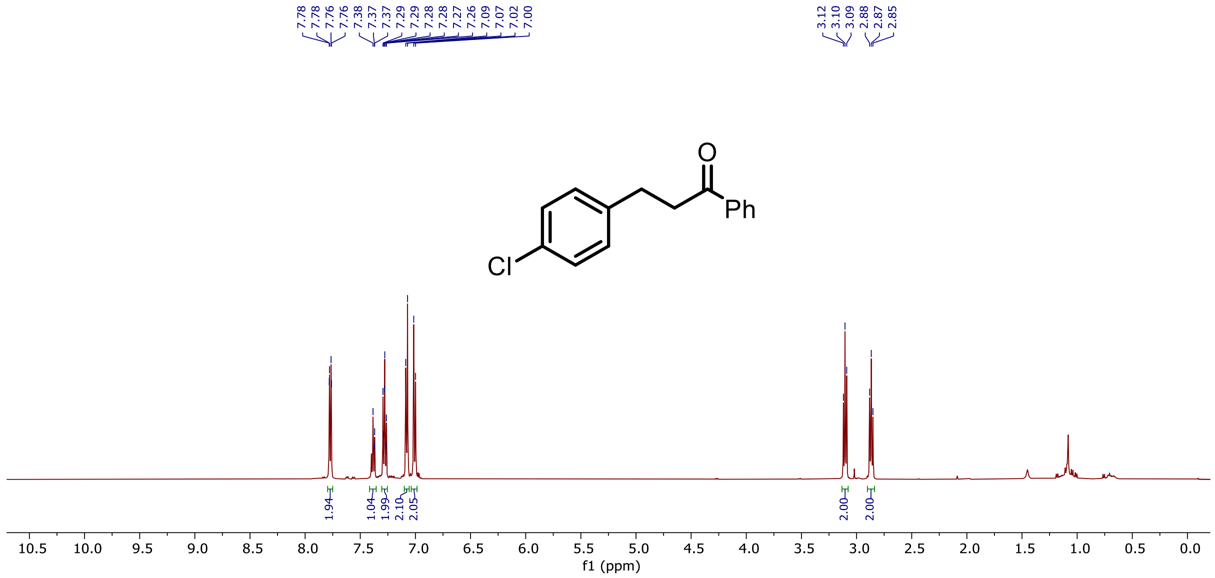

^{13}C NMR (101 MHz, $CDCl_3$)

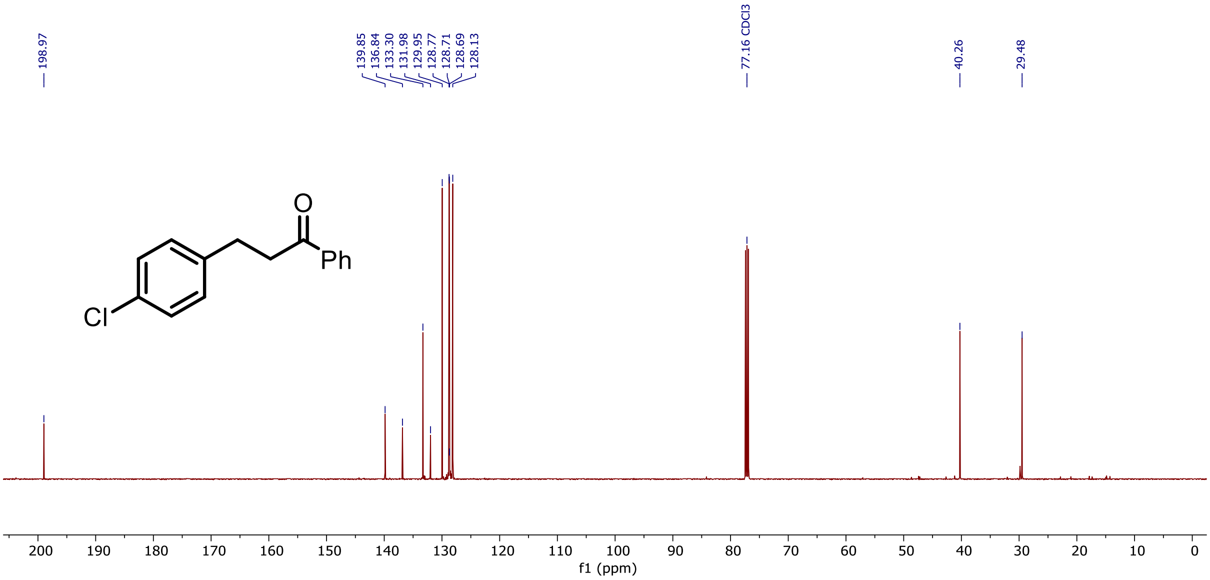

ethyl 3-phenylpropanoate (2r)

^{1}H NMR (400 MHz, $CDCl_3$)

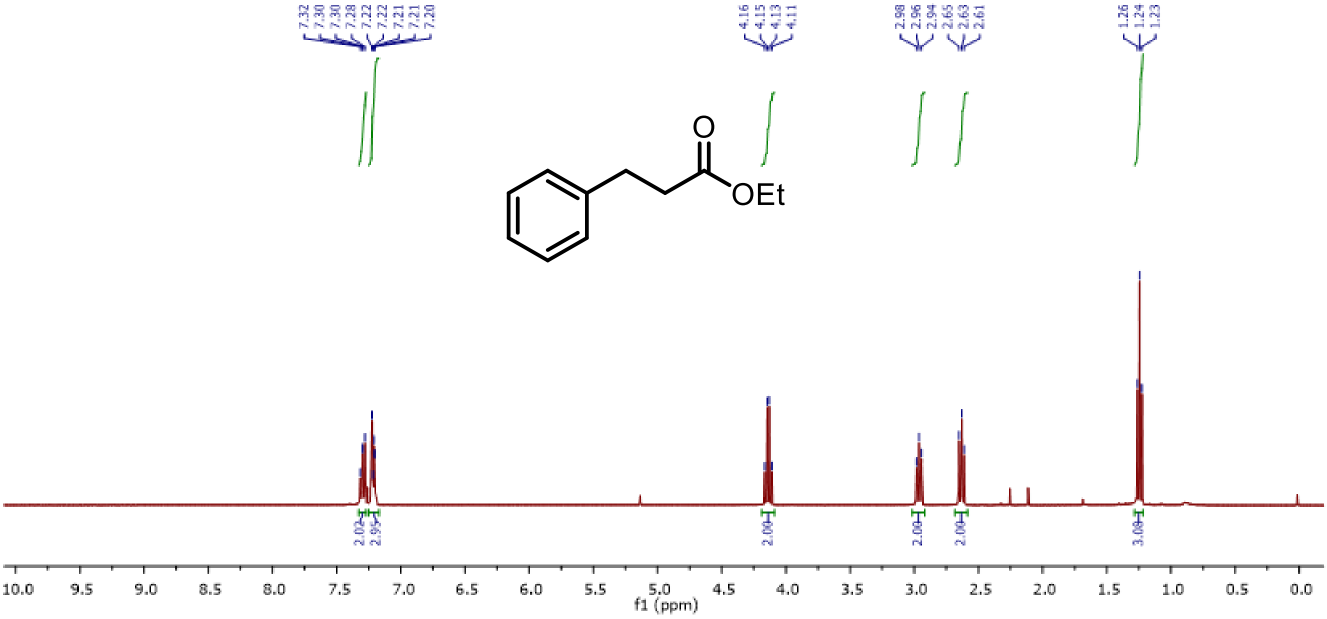

^{13}C NMR (101 MHz, CDCl$_3$)

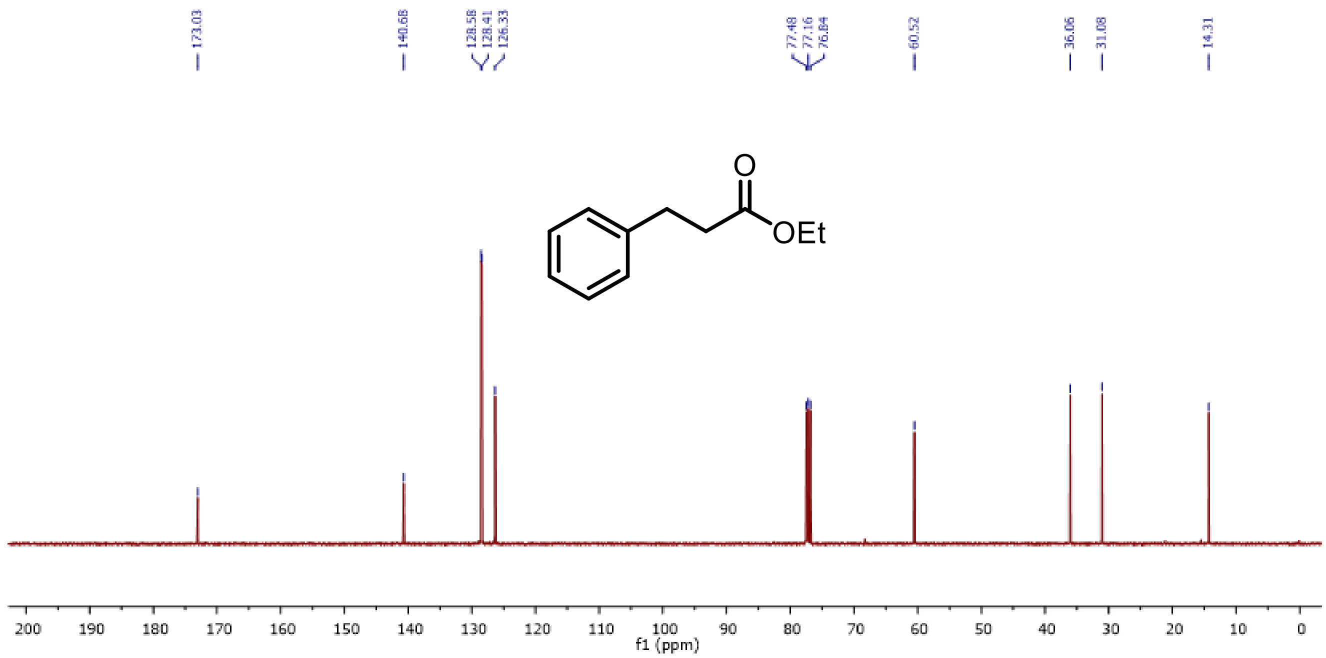

Methyl 3-phenylpropanoate (2s)

^{1}H NMR (400 MHz, CDCl$_3$)

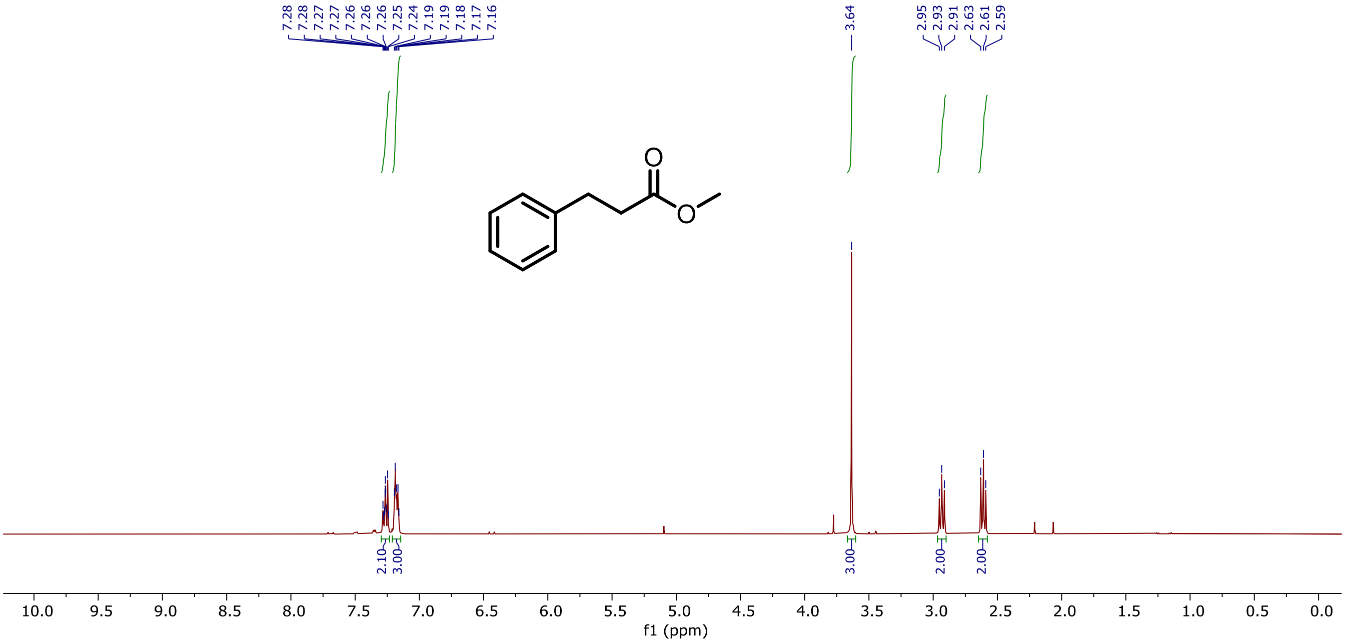

^{13}C NMR (101 MHz, $CDCl_3$)

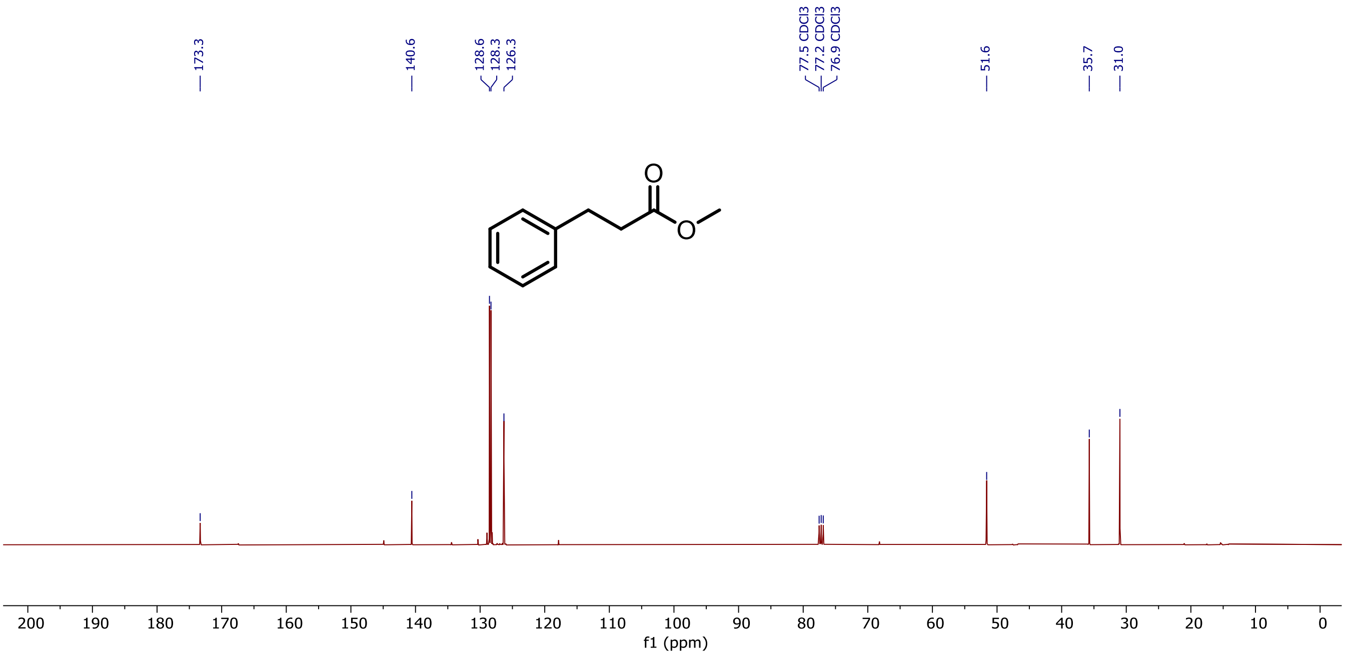

4-phenylbutan-2-one (2t)

^{1}H NMR (400 MHz, $CDCl_3$)

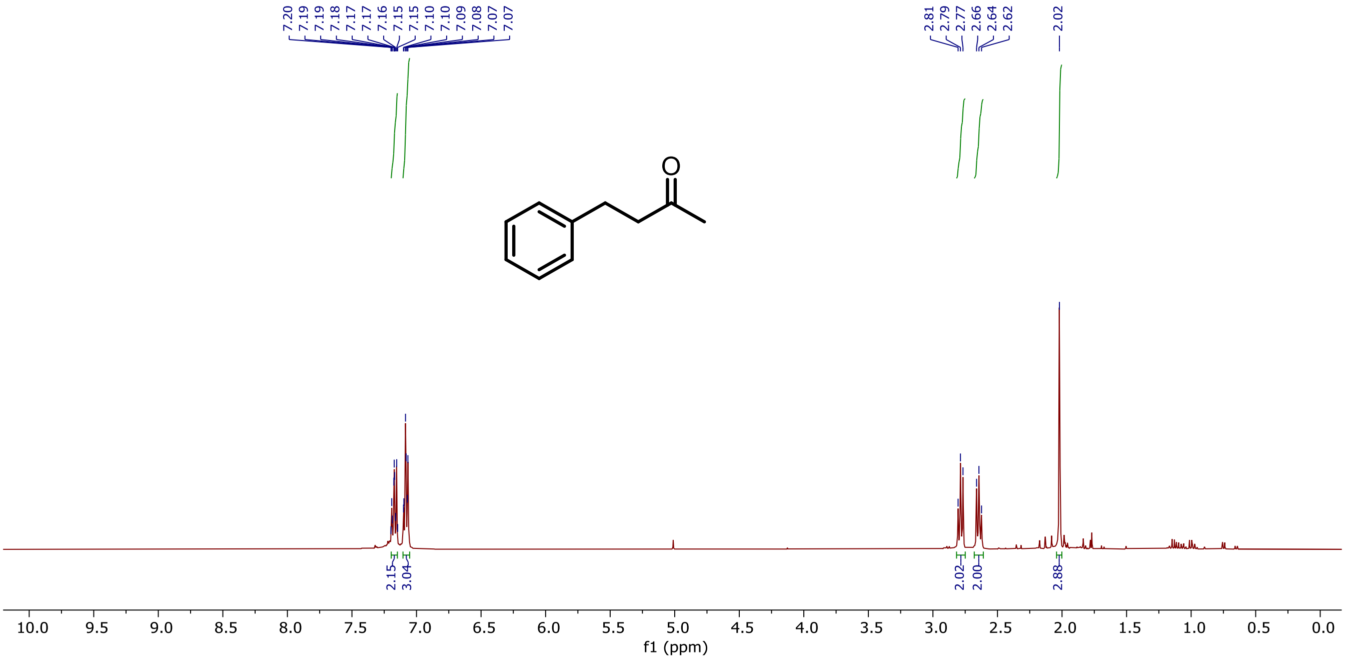

^{13}C NMR (101 MHz, CDCl$_3$)

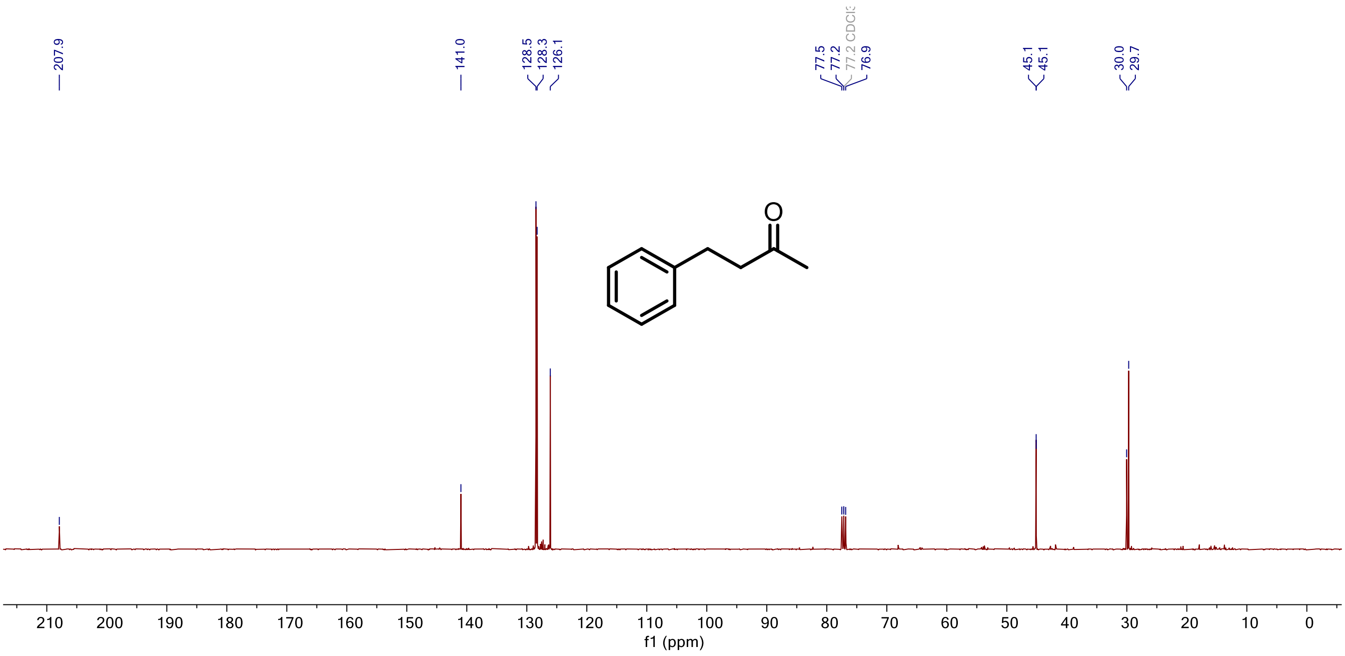

3-phenylpropanoic acid (2u)

^{1}H NMR (400 MHz, CDCl$_3$)

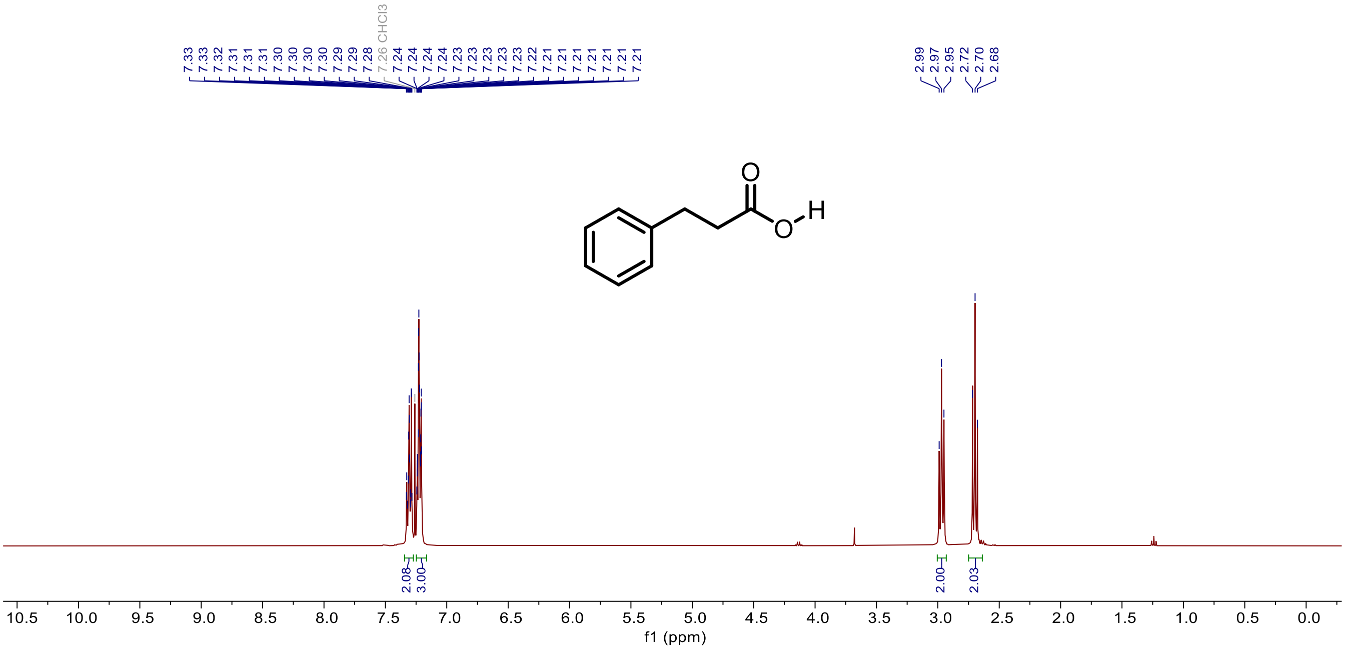

^{13}C NMR (101 MHz, $CDCl_3$)

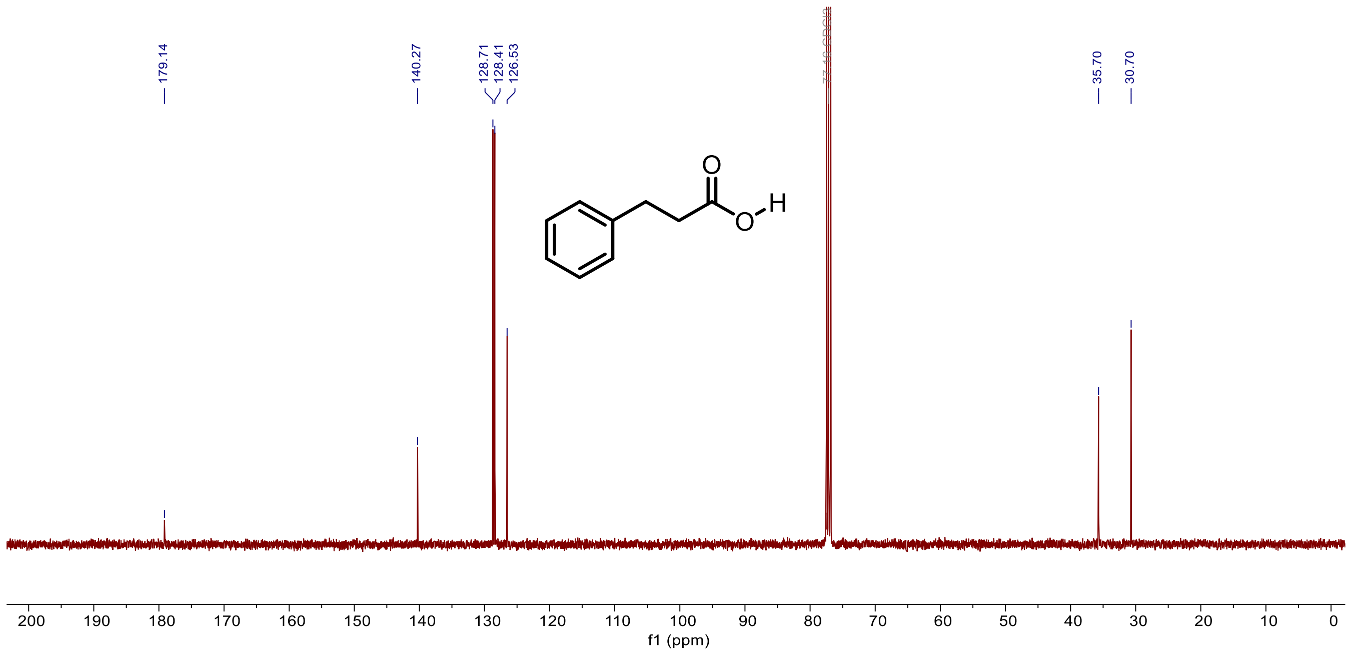

Supplement: Supplementary file 1 [file op6c00175_si_001.pdf]
